# Structure-Based Design of Novel Benzimidazole Derivatives as Pin1 Inhibitors

**DOI:** 10.3390/molecules24071198

**Published:** 2019-03-27

**Authors:** Shuxiang Wang, Lihong Guan, Jie Zang, Kun Xing, Jian Zhang, Dan Liu, Linxiang Zhao

**Affiliations:** Key Laboratory of Structure-Based Drug Design & Discovery of Ministry of Education, Shenyang Pharmaceutical University, Shenyang 110016, China; wangshuxiang10@163.com (S.W.); guanguan0919@163.com (L.G.); 15275312863@163.com (J.Z.); 15041472352@163.com (K.X.); ZJ2470703425@163.com (J.Z.)

**Keywords:** Pin1 inhibitors, benzimidazole derivatives, antiproliferative activity

## Abstract

Peptidyl-prolyl *cis/trans* isomerase Pin1 plays a key role in amplifying and translating multiple oncogenic signaling pathways during oncogenesis. The blockade of Pin1 provided a unique way of disrupting multiple oncogenic pathways and inducing apoptosis. Aiming to develop potent Pin1 inhibitors, a series of benzimidazole derivatives were designed and synthesized. Among the derivatives, compounds **6h** and **13g** showed the most potent Pin1 inhibitory activity with IC_50_ values of 0.64 and 0.37 μM, respectively. In vitro antiproliferative assay demonstrated that compounds **6d**, **6g**, **6h**, **6n**, **6o** and **7c** exhibited moderate antiproliferative activity against human prostate cancer PC-3 cells. Taken together, these unique benzimidazole derivatives exhibited great potential to be further explored as potent Pin1 inhibitors with improved potency.

## 1. Introduction

Protein interacting with Never in mitosis A1 (Pin1) is a peptidyl-prolyl isomerase, which catalyzes the *cis*-*trans* isomerization of prolyl amide bonds (pSer/Thr-Pro) in its substrate proteins to modulate their function and/or stability [1,2]. Proline-directed phosphorylation on serine or threonine is a common regulation mechanism in cells, it has been demonstrated that Pin1 can regulate diverse signaling processes in cell by functioning as a molecular switch and participate in cell cycle progression, apoptosis and immune responses [3,4,5]. In particular, many research suggested that Pin1 played a critical role in oncogenesis by upregulation of oncogenes and downregulation of tumor suppressors [6]. Therefore, it was speculated that inhibiting Pin1 might be an effective way to conquer the aggressive cancers by simultaneously impacting on multiple oncogenic signaling pathways. It was found that Pin1 is overexpressed in many human cancers, including prostate, breast, lung and colon cancer, and the overexpression of Pin1 is associated with aggressive tumor progression and poor prognosis in cancer [7,8,9]. Therefore, inhibiting Pin1 is expected to be an effective way for fighting against tumors.

**Figure 1:**
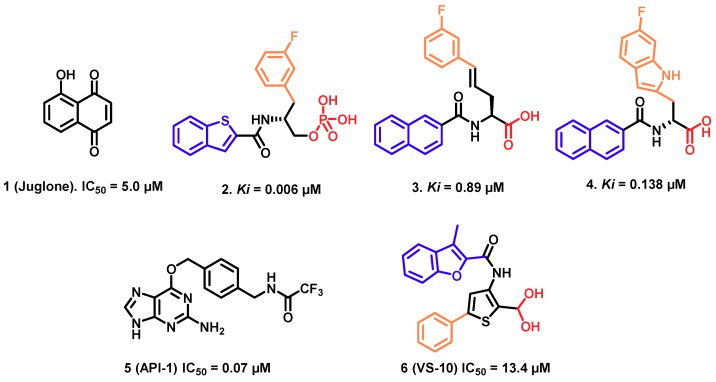
Chemical structures of known Pin1 inhibitors.

To date, a number of structurally distinct small molecule inhibitors of Pin1 have been reported (Figure 1). Juglone (**1**), a naturally occurring naphthoquinone compound [10], was found to be the first Pin1 inhibitor, which could inactivate Pin1 in an irreversible manner by covalently binding to active cysteine through Michael addition and has been widely used for the exploration of Pin1 biology in cells [11]. Researchers at Pfizer designed and synthesized several Pin1 inhibitors **2**–**4** by structure-based drug design, among which, compound **2** displayed the best Pin1 inhibitory activity [12,13,14]. However, compound **2** did not exhibit antiproliferative activities against tumor cells, the phosphate group conferring the compound poor permeability was the main reason for that. Pu et al. developed a specific, 6-*O*-benzylguanine skeleton-containing Pin1 inhibitor, API-1 (**5**), based on computer-aided virtual screening, API-1 directly and specifically binds to the Pin1 PPIase domain and potently inhibits the catalytic activity of Pin1 [15]. Virtual screening was used to identify potent Pin1 inhibitors by Spena et al., a potent Pin1 inhibitor VS10 (**6**) was identified and VS10 treatment reduced the viability of ovarian cancer cell lines by inducing proteasomal Pin1 degradation, without effects on Pin1 transcription [16]. Although tremendous works have been done, there have been no desirable inhibitors with potent activity in cellular level reported due to either poor physicochemical properties or low potency in enzymatic activity. Thus, it is challenging to discover highly potent Pin1 inhibitors with drug-like properties. Spena et al. encapsulated compound **4** efficiently in modified cyclodextrins and loaded them into pegylated liposomes, this liposomal fromulation accumulates preferentially in tumor and has a desirable pharmacokinetic profile [17]. Recently, it was reported that all-trans retinoic acid (ATRA), a therapeutic agent for acute promyelocytic leukemia, could inhibit and degrade Pin1 in cancer cells, which demonstrated a viable way for using Pin1 inhibitors as anti-cancer agents [18]. However, the efficacy of ATRA against solid tumors is limited due to its short half-life of 45 min in humans, to overcame the poor pharmacokenitic profiles of ATRA, a novel controlled release fromulation of ATRA-PLLA (poly L-lactic acid) microparticles was developed, which exhibited good biocompatibility, and could significantly enhance the inhibition of ATRA on HCC cell growth and dramatically decrease the dose of ATRA in both in vitro and in vivo systems [19].

The crystal structures of small molecule inhibitors complexes with Pin1 indicated that the unique substrate binding site of Pin1 consisting of the following subpockets, including the prolyl pocket fromed by His59, His157, Met130 and Phe13, a slightly shallow hydrophobic shelf lined fromed by Ala118 and Leu22, and a unique phosphate binding pocket embracing Lys63, Arg68 and Arg69. The aryl scaffold, hydrophobic side chains and phosphate group or carboxylic group of known Pin1 inhibitors such as compounds **2**–**4** occupied the corresponding pocket respectively and inhibited the activity of Pin1 [12,13,14,20,21,22].

**Figure 2:**
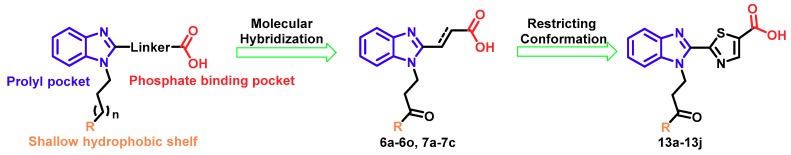
Design strategies of novel Pin1 inhibitors.

In our design of Pin1 inhibitors, a bioactive benzimidazole was selected as the scaffold to occupy the prolyl pocket and a flexible hydrophobic side chain was connected to the 1-N of benzimidazole scaffold to occupy the slightly shallow hydrophobic-lined shelf (Figure 2). A docking study indicated that the carboxyl can be linked to the 2-C position of benzimidazole by a suitable linker; this carboxyl was expected to from H-bonds with basic amino acid residues in the phosphate binding pocket, and the linker needed to possess a suitable length and spatial conformation to ensure that the carboxyl group extends into the phosphate binding pocket accurately. It is reported that cinnamic acid derivatives displayed inhibitory activity on Pin1 [23], so selecting the vinyl group as the linker was expected to meet the conditions mentioned. Three other compounds with -CH_2_CH_2_- as the linker were designed to verify the design strategy. At the same time, replacement of the double bond by a thiazole ring could further restrict the conformation of the compounds and ensure establishment of strong interactions between the carboxyl and basic amino acid residues of the phosphate binding pocket, which should be helpful to improve the activity of these compounds. Based on these ideas, a series of novel (*E*)-3-(1*H*-benzo[*d*]imidazol-2-yl)acrylic acids and 5-(1*H*-benzo[*d*]imidazol-2-yl) thiazole-2-carboxylic acid derivatives featured with various 1-N substituents were designed and synthesized.

## 2. Results and Discussion

### 2.1. Chemistry

**Scheme 1:**
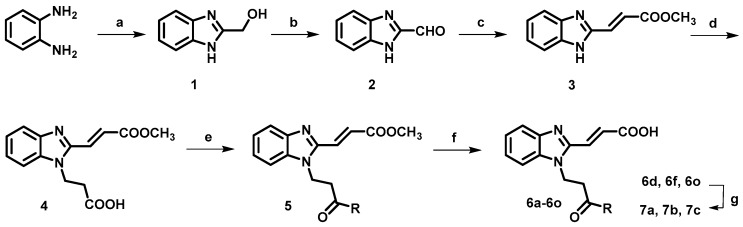
Synthesis of **6a**–**6o** and **7a**–**7c**. Reagents and Conditions: (a) CH_2_(OH)COOH, 4N HCl/H_2_O, 100 °C; (b) MnO_2_, CH_2_Cl_2_, reflux; (c) methyl diethylphosphonoacetate, K_2_CO_3_, dry THF, reflux; (d) 3-bromopropionic acid, K_2_CO_3_, acetone/DMF/H_2_O=5/1/0.1, reflux; (e) piperidine or morpholine or RNH_2_, EDCI, HOBt, 4-methylmorpholine, CH_2_Cl_2_, r.t.; (f) LiOH, THF/H_2_O, r.t.; (g) H_2_, Pd/C, r.t.

The synthesis route of **6a**–**6o** and **7a**–**7c** is described in Scheme 1. The commercial material *o*-phenylenediamine was selected as starting material, and compound **1** was obtained by condensation and cyclization of *o*-phenylenediamine and glycolic acid. Oxidation of the C2-hydroxymethyl of **1** with manganese dioxide yielded aldehyde product **2**. Intermediate **3** was generated in good yield by a Wittig-Horner reaction with methyl diethylphosphonoacetate, and then alkylation with 3-bromopropionic acid produced key intermediate **4**. Reaction of carboxylic acid **4** with amines afforded the corresponding amides, and treatment of these amides with lithium hydroxide afforded **6a**–**6o**. Compounds **7a**–**7c** were obtained by reduction of **6d**, **6f** and **6o**, respectively.

**Scheme 2:**
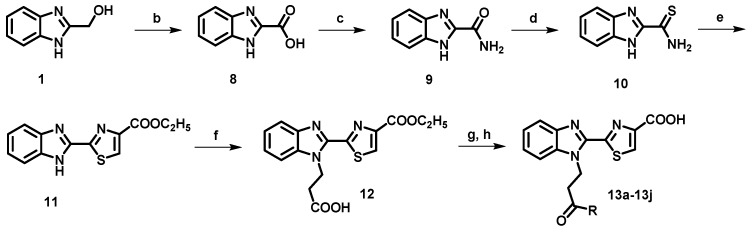
Synthesis of **13a**–**13j**. Reagents and Conditions: (a) CH_2_(OH)COOH, 4N HCl/H_2_O, 100 °C; (b) KMnO_4_, H_2_O, reflux; (c) SOCl_2_, NH_3_·H_2_O, 70 °C; (d) Lawsson’s reagent, THF, 66 °C; (e) ethyl 3-bromo-2-oxopropanoate, ethanol, 78 °C; (f) 3-bromopropionic acid, K_2_CO_3_, acetone/DMF/H_2_O, 70 °C; (g) RNH_2_, EDCI, HOBt, 4-methylmorpholine, CH_2_Cl_2_, r.t.; (h) LiOH, THF/H_2_O, r.t.

The synthesis of **13a**–**13j** is described in Scheme 2. Oxidization of **1** with potassium permanganate afforded 1*H*-benzo[*d*]imidazole-2-carboxylic acid (**8**). Conversion of the carboxylic acid group of **8** to a carbamoyl group was achieved by first froming the acyl chloride and subsequently reacting it with ammonium hydroxide to yield compound **9**. Thionation of the carbonyl of compound **9** with Lawsson’s reagents gave **10**. Condensation and cyclization of compound **10** using ethyl 3-bromo-2-oxopropanoate gave 2-(1*H*-benzo[*d*]imidazol-2-yl)thiazole intermediate **11**. Alkylation of **11** with 3-bromopropionic acid afforded the desired product **12**. Coupling of the carboxylic acid **12** with amines yielded the corresponding amides, which were finally reacted with lithium hydroxide to afford final products **13a**–**13j**. 

### 2.2. Biological Activity

To investigate the Pin1 inhibitory potency of all synthesized compounds, the inhibition ratio at 10 μM or IC_50_ of all synthesized compounds was determined using protease-coupled assay. The results for all target compounds are shown in Table 1 and Table 2. 

The results indicate that a portion of the target compounds exhibited promising activities towards Pin1, among which, most of the compounds displayed an inhibition ratio >50% at 10 μM. Compounds **6a** and **6b** with piperidyl or morpholinyl substituents exhibited little potency (28% and 27% at 10 μM, respectively); replacing piperidyl or morpholinyl with phenyl (compound **6c**) led to increased inhibitory activity (46%), indicating that aromatic ring is beneficial to the Pin1 inhibition, so the following compounds employed phenyl as substituent, and a more systematic study of the substituents on the phenyl moiety was carried out. 4-Methyl-substituent compound **6d** exhibited elevated inhibitory activity, while compounds **6e** or **6f** with 4-methoxy or 4-chloro substituents demonstrated decreased activities, which indicated that introducing electron-donating substituents on the phenyl is helpful to Pin1 inhibition. What’s more, it is worth noting that compound **6h** with a 3-Br substituent showed the best Pin1 inhibitory activity among **6a**–**6o**, with an IC_50_ value of 0.64 μM, suggesting that the bromine atom is preferable, presumably to interact with the amino acid residues via forming an H-halogen bond. The effect of extending the substituent (compounds **6g**, **6j** and **6k** or **6e**, **6l** and **6m**) on activity is weak. Increasing the number of substituents on the phenyl may improve the activity of the compounds to different degrees, but trimethyl-substituent compound **6o** did not show a prominent advantage over compound **6d**. Compounds **7a**, **7b** and **7c** were obtained by reduction of **6d**, **6f** and **6o**, respectively. Compared to compounds **6d**, **6f** and **6o**, the activity of compounds **7a**–**7c** showed different degrees of decline, proving that the double bond is a necessary group for activity, which confirmed our design idea. 

In order to further restrict the conformation of the compound, the double bond was replaced by a thiazole ring (compounds **13a**–**13j**). The results indicated that most of the compounds with thiazole rings as the linker displayed better inhibitory activity than double bond compounds. Compound **13a** with a phenyl substituent exhibited significantly improved activity compared to **6a**, and a more systematic study of the substituents on the phenyl ring was carried out. Compounds **13b** and **13c** with 3-Br or 2-Br substituents showed similar activity. Replacing the 3-Br with 3-OCH_3_ or 3,5-diOCH_3_ afforded **13d** and **13e**, which displayed improved activity compared to **13a**, but there was no significant difference in the activity of the two compounds. Introducing a NO_2_ into the C3 position (compound **13f**) reduced the activity. Replacing the NO_2_ with CF_3_ afforded **13g**, which showed the best Pin1 inhibitory activity with an IC_50_ value of 0.37 μM. Compounds **13h** and **13i** with 4-CF_3_ or 3,5-diCl substituents displayed reduced activity, respectively. 

The structure-activity relationship analysis indicated that compared with the number of substituents, the kinds of substituents influenced the Pin1 inhibitory activity more. As mentioned above, most of the Pin1 small molecule inhibitors did not exhibit promising antiproliferative activities due to Pin1 specifically recognizing a phosphorylated Ser/Thr motif in its substrate binding site, and consequently potent Pin1 inhibitors commonly contain a negatively double-charged phosphate group or carboxyl group, and the phosphate or carboxyl group can be detrimental to inhibitor cell permeability which hinders activity in a whole cell assay. It is still challenging to discover highly potent Pin1 inhibitors with drug-like properties. As for Pin1 was overexpressed in human prostate cancer cells, the antiproliferative activity of these benzimidazole derivatives against PC-3 cells were further evaluated by MTT assay. Meanwhile, the polar surface area (PSA) of these compounds was calculated under the optimal conformation since PSA is the key indicator of membrane permeability. As shown in Table 3, most of the compounds showed hardly any antiproliferative activity against PC-3 cells (GI_50_ > 50 μM), except compounds **6d**, **6g**, **6h**, **6n**, **6o** and **7c**, which demonstrated moderate antiproliferative activity, with GI_50_ values between 9.89 to 28.57 μM. Compounds **6a**–**6o** and **7a**–**7c** exhibited a polar surface area between 150 and 200, while compound **13a**–**13j** showed a polar surface area > 200, indicating that the membrane permeability of **13a**–**13j** is worse than that of **6a**–**6o** and **7a**–**7c**, which is probably the reason why compounds **13a**–**13j** exhibited stronger Pin1 inhibitory activities but worse antiproliferative activities than **6a**–**6o** and **7a**–**7c**.

## 3. Materials and Methods

### 3.1. Chemistry

Melting points were measured with an X-4 digital micro melting point apparatus (Shanghai Jingke Co. Ltd, Shanghai, China) and are uncirrected. ^1^H-NMR spectra were measured with a Bruker ARX (400 MHz) spectrometer and ^13^C-NMR with a Bruker AV (150 MHz) instrument (Bruker, Karlsruhe, Germany). Chemical shifts are recorded in δ units using tetramethylsilane as the standard (NMR peak description: s, singlet; d, doublet; t, triplet; q, quartet; m, multiplet; br, broad peak). Low resolution mass spectrometry (MS) were recorded with an Agilent 1100 ion trap liquid chromatography-mass spectrometer (Agilent, Santa Clara, CA, USA). Organic solutions were dried over anhydrous sodium sulfate during workup. Column chromatography was carried out on a Combiflash Rf+ preparative liquid chromatograph (Teledyne ISCO, NE, USA). Silica gel 60 (200–300 mesh) and TLC were purchased from Qingdao Haiyang Chemical Co. Ltd. (Qingdao, China) All commercial reagents and solvents were used without further purification unless otherwise noted. The purity of all compounds screened in biological assays was >95% pure as judged by HPLC. HPLC analysis were obtained on an L-2400 system (Hitachi, Kyoto, Japan) using a AichromBond-AQ C18 column (150 mm × 4.6 mm, 5 μm) with a 1.5 mL/min flow rate using acetonitrile and water solution (*v*/*v* = 70:30) as the eluent over 30 min.

The original figures of ^1^H-NMR, ^13^C-NMR and MS of all the target compounds as the Supplementary Materials are available online.

#### 3.1.1. General Procedure for the Synthesis of **6a**–**6o**

*(1H-Benzo[d]imidazol-2-yl) methanol* (**1**): To a solution of 1,2-diaminobenzene (10.0 g, 92.6 mmol), in 4 N HCl (80 mL), glycolic acid (20.0 g, 263 mmol) was added and stirred for 4 h at 100 °C and monitored by TLC. After complete conversion of starting material, cooled the solution to room temperature, the pH of the solution was adjusted to 8 with a 2 mol/L aqueous sodium hydroxide solution, the precipitate was filtered and dried to afford **1** as white solid in 88.0% yield. LC-MS *m*/*z*: 149.2 [M + H]^+^.

*1H-Benzo[d]imidazole-2-carbaldehyde* (**2**): To a solution of **1** (5.0 g, 34 mmol) in DCM was added MnO_2_ (1.3 g, 6.8 mmol). The resulting solution was stirred at 40 °C for 2 h and monitored by TLC. After complete conversion of starting material, reaction mixture was cooled to room temperature, filtered and concentrated in vacuo to obtain **2** as white solid in 85.0% yield. LC-MS *m*/*z*: 147.1 [M + H]^+^.

*Methyl (E)-3-(1H-benzo[d]imidazol-2-yl) acrylate* (**3**): To a solution of methyl diethylphosphonoacetate (3.17g, 15.0 mmol) in dry THF (40 mL) was added sodium carbonate (3.79 g, 27.4 mmol), the mixture was stirred for 30 min at room temperature prior to the addition of compound **2** (2.0 g, 13.7 mmol). The mixture was stirred and refluxed for 24 h under an argon atmosphere and monitored by TLC. After complete conversion of starting material, the reaction mixture was filtered, the filtrate was concentrated and re-dissolved by ethyl acetate, and then washed with saturated NaCl solution and dried over anhydrous sodium sulfate, concentrated in vacuo and purified by flash silica gel column (PE/EA = 4/1, *v*/*v*) to obtain **3** as white solid in 87.5% yield. LC-MS *m*/*z*: 203.1 [M + H]^+^.

*(E)-3-(2-(3-Methoxy-3-oxoprop-1-en-1-yl)-1H-benzo[d]imidazol-1-yl) propanoic acid* (**4**): To a solution of **3** (0.4 g, 2.0 mmol) in DMF (10 mL) and acetone (40 mL) was added 3-bromopropionic acid (1.2 g, 8.0 mmol) and a solution of K_2_CO_3_ (5.5 g, 40 mmol) in water (0.8 mL), the mixture was stirred at 70 °C for 4 h. After complete conversion of starting material, the reaction mixture was cooled to room temperature, concentrated in vacuo and redissolved in water (50 mL). The resulting solution was adjusted to pH 5 with a 6 N HCl at 0 °C, and then extracted with ethyl acetate, the organic layers combined and washed with saturated NaCl, dried over anhydrous sodium sulfate, filtered, and concentrated in vacuo to obtain **4** as a white solid in 82.5% yield. LC-MS *m*/*z*: 273.1 [M − H] ^−^.

*(E)-3-(1-(3-oxo-3-(piperidin-1-yl)propyl)-1H-benzo[d]imidazol-2-yl)acrylic acid* (**6a**): To a mixture of compound **4** (0.47 g, 1.73 mmol) and piperidine (0.16 g, 1.9 mmol) in dried DCM (50 mL) was added 4-methylmorpholine (0.97 mL) followed by HOBt (0.47 g 3.46 mmol) at 0 °C, and EDCI (0.66 g, 3.46 mmol) was added in batches. After stirring for 30 min, the mixture was warmed to room temperature and stirred overnight. The reaction mixture was washed by saturated NaCl solution three times and dried over anhydrous sodium sulfate, concentrated and purified by flash silica gel column (PE/EA = 1/3, *v*/*v*), the residue was dissolved in THF (6 mL), then lithium hydroxide solution (3.5 mmol/mL, 1.6 mL) was added dropwise. The resulting solution was stirred at room temperature for 6 h then concentrated in vacuo, and the residue re-dissolved by water and acidified with 2N HCl to pH 5 at 0 °C. Filtered and dried to obtain the desired compounds **6a** as white solid in 58.5% yield. m.p.: 128–130 °C. ^1^H-NMR (DMSO-*d*_6_) δ: 12.74 (s, 1H), 7.79 (d, *J* = 15.4 Hz, 1H), 7.67 (m, 1H), 7.66 (m, 1H), 7.33 (m, *J* = 9.04 Hz, 2H), 6.90 (d, *J* = 15.4 Hz, 1H), 4.60 (t, *J* = 6.6 Hz, 2H), 3.34 (t, *J* = 5.44 Hz, 2H), 3.21 (t, *J* = 5.4 Hz, 2H), 2.84 (t, *J* = 6.7 Hz, 2H), 1.47 (m, *J* = 5.32 Hz, 2H), 1.32 (m, *J* = 3.9 Hz, 2H), 1.26 (m, *J* = 7.4 Hz, 2H). LC-MS *m*/*z*: 328.2 [M + H]^+^.

*(E)-3-(1-(3-Morpholino-3-oxopropyl)-1H-benzo[d]imidazol-2-yl) acrylic acid* (**6b**): Compound **6b** was prepared from **4** and morpholine in a similar manner as compound **6a**. White solid. Yield 51.2%. m.p.: 207–209 °C. ^1^H-NMR (DMSO-*d*_6_) δ: 12.81 (s, 1H), 7.79 (d, *J* = 15.4 Hz, 1H), 7.66 (t, *J* = 7.36 Hz, 2H), 7.28 (m, *J* = 7.2, 3.6 Hz, 2H), 6.91 (d, *J* = 15.4 Hz, 1H), 4.61 (t, *J* = 6.7 Hz, 2H), 3.44 (t, *J* = 9.36 Hz, 2H), 3.37 (t, *J* = 4.16Hz, 4H), 3.26 (t, *J* = 9.36 Hz, 2H), 2.86 (t, *J* = 6.7 Hz, 2H). LC-MS *m*/*z*: 330.1 [M + H]^+^.

*(E)-3-(1-(3-Oxo-3-(phenylamino)propyl)-1H-benzo[d]imidazol-2-yl)acrylic acid* (**6c**): Compound **6c** was prepared from **4** and aniline in a similar manner as compound **6a**. White solid. Yield 59.5%. m.p.: 228–230 °C. ^1^H-NMR (DMSO-*d*_6_) δ: 12.89 (s, 1H), 9.95 (s, 1H), 7.81 (d, *J* = 15.4 Hz, 1H), 7.67 (t, *J* = 8.9 Hz, 2H), 7.46 (d, *J* = 8.0 Hz, 2H), 7.30 (t, *J* = 7.5 Hz, 1H), 7.24 (t, *J* = 7.8 Hz, 3H), 7.00 (t, *J* = 7.4 Hz, 1H), 6.91 (d, *J* = 15.4 Hz, 1H), 4.71 (t, *J* = 6.3 Hz, 2H), 2.85 (t, *J* = 6.4 Hz, 2H). LC-MS *m*/*z*: 336.1 [M + H]^+^.

*(E)-3-(1-(3-Oxo-3-(p-tolylamino)propyl)-1H-benzo[d]imidazol-2-yl)acrylic acid* (**6d**): Compound **6d** was prepared from **4** and *p*-toluidine in a similar manner as compound **6a**. White solid. Yield 54.1%. m.p.: 221–222 °C. ^1^H-NMR (DMSO-*d*_6_) δ: 12.93 (s, 1H), 9.88 (s, 1H), 7.80 (d, *J* = 15.4 Hz, 1H), 7.67 (t, *J* = 7.8 Hz, 2H), 7.35 (d, *J* = 8.4 Hz, 2H), 7.30 (t, *J* = 7.5 Hz, 1H), 7.24 (t, *J* = 7.5 Hz, 1H), 7.04 (d, *J* = 8.3 Hz, 2H), 6.91 (d, *J* = 15.4 Hz, 1H), 4.70 (t, *J* = 6.4 Hz, 2H), 2.83 (t, *J* = 6.4 Hz, 2H), 2.21 (s, 3H). LC-MS *m*/*z*: 350.1 [M + H]^+^.

*(E)-3-(1-(3-((4-Methoxyphenyl)amino)-3-oxopropyl)-1H-benzo[d]imidazol-2-yl)acrylic acid* (**6e**): This compound was prepared from **4** and 4-methoxyaniline in a similar manner as compound **6a**. White solid. Yield 53.6%. m.p.: 215–217 °C. ^1^H-NMR (DMSO-*d*_6_) δ: 12.80 (s, 1H), 9.80 (s, 1H), 7.81 (d, *J* = 15.4 Hz, 1H), 7.67 (t, *J* = 7.3 Hz, 2H), 7.36 (d, *J* = 8.9 Hz, 2H), 7.32 (s, 1H), 7.25 (t, *J* = 7.6 Hz, 1H), 6.90 (d, *J* = 15.4 Hz, 1H), 6.82 (d, *J* = 8.9 Hz, 2H), 4.70 (t, *J* = 6.2 Hz, 2H), 3.69 (s, 3H), 2.80 (t, *J* = 6.3 Hz, 2H). LC-MS *m*/*z*: 366.1 [M + H]^+^.

*(E)-3-(1-(3-((4-Chlorophenyl)amino)-3-oxopropyl)-1H-benzo[d]imidazol-2-yl)acrylic acid (***6f**): Compound **6f** was prepared from **4** and 4-chloroaniline in a similar manner as compound **6a**. White solid. Yield 54.8%. m.p.: 158–160 °C. ^1^H-NMR (DMSO-*d*_6_) δ: 10.23 (s, 1H), 7.67 (d, *J* = 15.3 Hz, 1H), 7.64 (t, *J* = 8.2 Hz, 2H), 7.52 (d, *J* = 8.8 Hz, 2H), 7.30 (d, *J* = 8.8 Hz, 2H), 7.25 (t, 1H), 7.22(t, *J* = 7.1 Hz, 1H), 6.95 (d, *J* = 15.3 Hz, 1H), 4.68 (t, *J* = 6.2 Hz, 2H), 2.86 (t, *J* = 6.3 Hz, 2H). LC-MS *m*/*z*: 370.1 [M + H]^+^.

*(E)-3-(1-(3-((4-Bromophenyl)amino)-3-oxopropyl)-1H-benzo[d]imidazol-2-yl)acrylic acid (***6g**): Compound **6g** was prepared from **4** and 4-bromoaniline in a similar manner as compound **6a**. White solid. Yield 48.0%. m.p.: 192–194 °C. ^1^H-NMR (DMSO-*d*_6_) δ: 10.18 (s, 1H), 7.66 (d, *J* = 15.6 Hz, 1H),7.64 (t, *J* = 5.5 Hz, 2H), 7.46 (m, *J* = 6.7 Hz, 4H), 7.26 (t, *J* = 7.2 Hz, 1H), 7.22 (t, *J* = 7.2 Hz, 1H), 6.94 (d, *J* = 15.6 Hz, 1H), 4.68 (t, *J* = 5.8 Hz, 2H), 2.86 (t, *J* = 5.8 Hz, 2H). LC-MS *m*/*z*: 414.0 [M + H]^+^.

*(E)-3-(1-(3-((3-Bromophenyl)amino)-3-oxopropyl)-1H-benzo[d]imidazol-2-yl)acrylic acid* (**6h**): Compound **6h** was prepared from **4** and 3-bromoaniline in a similar manner as compound **6a**. White solid. Yield 58.9%. m.p.: 209–210 °C. ^1^H-NMR (DMSO-*d*_6_) δ: 12.46 (s, 1H), 10.15 (s, 1H), 7.80 (s, 1H), 7.78 (d, *J* = 15.4 Hz, 1H), 7.67 (t, *J* = 6.9 Hz, 2H), 7.36 (d, *J* = 6.56 Hz, 1H), 7.30 (t, *J* = 7.24 Hz, 1H), 7.24 (t, *J* = 7.68 Hz, 1H), 7.21 (d, *J* = 6.28 Hz 1H), 7.20 (s, 1H), 6.92 (d, *J* = 15.4 Hz, 1H), 4.71 (t, *J* = 6.3 Hz, 2H), 2.86 (t, *J* = 6.3 Hz, 2H). LC-MS *m*/*z*: 416.1 [M + H]^+^.

*(E)-3-(1-(3-((3,5-dimethoxyphenyl)amino)-3-oxopropyl)-1H-benzo[d]imidazol-2-yl)acrylic acid* (**6i**): Compound **6i** was prepared from **4** and 3,5-dimethoxyaniline in a similar manner as compound **6a**. White solid. Yield 53.1%. m.p.: 203–204 °C. ^1^H-NMR (DMSO-*d*_6_) δ: 12.81(s, 1H), 9.90 (s, 1H), 7.81 (d, *J* = 15.4 Hz, 1H), 7.67 (t, *J* = 7.5 Hz, 2H), 7.31 (t, *J* = 7.5 Hz, 1H), 7.25 (t, *J* = 7.8 Hz, 1H), 6.91 (d, *J* = 15.4 Hz, 1H), 6.87 (s, 1H), 6.71 (s, 2H), 4.70 (t, *J* = 6.4 Hz, 2H), 3.67 (s, 6H), 2.82 (t, *J* = 6.4 Hz, 2H). LC-MS *m*/*z*: 396.1 [M + H]^+^.

*(E)-3-(1-(3-((4-Bromobenzyl)amino)-3-oxopropyl)-1H-benzo[d]imidazol-2-yl)acrylic acid* (**6j**): Compound **6j** was prepared from **4** and (4-bromophenyl)methanamine in a similar manner as compound **6a**. White solid. Yield 55.8%. m.p.: 210–212 °C. ^1^H-NMR (DMSO-*d*_6_) δ: 12.78 (s, 1H), 8.42 (t, *J* = 5.9 Hz, 1H), 7.77 (d, *J* = 15.4 Hz, 1H), 7.72–7.66 (m, 1H), 7.66–7.58 (m, 1H), 7.35 (d, *J* = 8.4 Hz, 2H), 7.33–7.22 (m, 2H), 6.89 (d, *J* = 15.4 Hz, 3H), 4.65 (t, *J* = 6.3 Hz, 2H), 4.11 (d, *J* = 5.8 Hz, 2H), 2.68 (t, *J* = 6.3 Hz, 2H). LC-MS *m*/*z*: 430.1 [M + H]^+^.

*(E)-3-(1-(3-((4-Bromophenethyl)amino)-3-oxopropyl)-1H-benzo[d]imidazol-2-yl)acrylic acid (***6k**): Compound **6k** was prepared from **4** and 2-(4-bromophenyl)ethan-1-amine in a similar manner as compound **6a**. White solid. Yield 59.9%. m.p.: 203–205 °C. ^1^H-NMR (DMSO-*d*_6_) δ: 12.82(s, 1H), 8.00 (t, *J* = 5.4 Hz, 1H), 7.71–7.59 (m, 3H), 7.34 (d, *J* = 8.3 Hz, 2H), 7.31–7.21 (m, 2H), 6.93 (d, *J* = 15.5 Hz, 1H), 6.89 (d, *J* = 8.3 Hz, 2H), 4.59 (t, *J* = 6.2 Hz, 2H), 3.12 (dd, *J* = 12.7, 6.7 Hz, 2H), 2.56 (t, *J* = 6.3 Hz, 2H), 2.46 (t, *J* = 7.1 Hz, 2H). ^13^C-NMR (DMSO-*d*_6_) δ 169.3, 167.2, 148.1, 142.7, 138.8, 135.3, 131.0, 130.9, 128.5, 127.1, 123.1, 122.7, 119.4, 119.1, 111.1, 40.1, 36.0, 34.1, 30.5. LC-MS *m*/*z*: 442.0 [M + H]^+^.

*(E)-3-(1-(3-((4-Methoxybenzyl)amino)-3-oxopropyl)-1H-benzo[d]imidazol-2-yl)acrylic acid* (**6l**): Compound **6l** was prepared from **4** and (4-methoxyphenyl)methanamine in a similar manner as compound **6a**. White solid. Yield 54.3%. m.p.: 203–205 °C. ^1^H-NMR (DMSO-*d*_6_) δ: 12.85 (s, 1H), 8.32 (t, *J* = 5.7 Hz, 1H), 7.78 (d, *J* = 15.4 Hz, 1H), 7.69 (d, *J* = 7.16 Hz, 1H), 7.63 (d, *J* = 7.16 Hz, 1H), 7.29 (m, *J* = 7.1 Hz, 2H), 6.89 (d, *J* = 8.6 Hz, 3H), 6.75 (d, *J* = 9.1 Hz, 2H), 4.65 (t, *J* = 6.3 Hz, 2H), 4.08 (d, *J* = 5.7 Hz, 2H), 3.70 (s, 3H), 2.66 (t, *J* = 6.3 Hz, 2H). ^13^C-NMR (DMSO-*d*_6_) δ 169.2, 166.9, 158.1, 147.7, 142.7, 135.4, 130.9, 129.6, 128.5, 125.4, 123.3, 122.8, 119.5, 113.6, 111.2, 55.0, 41.6, 40.1, 36.0. LC-MS *m*/*z*: 380.1 [M + H]^+^.

*(E)-3-(1-(3-((4-Methoxyphenethyl)amino)-3-oxopropyl)-1H-benzo[d]imidazol-2-yl)acrylic acid* (**6m**)*:* Compound **6m** was prepared from **4** and 2-(4-methoxyphenyl)ethan-1-amine in a similar manner as compound **6a**. White solid. Yield 51.9%. m.p.: 219–220 °C. ^1^H-NMR (DMSO-*d*_6_) δ: 12.85 (s, 1H), 8.32 (t, *J* = 5.7 Hz, 1H), 7.78 (d, *J* = 15.4 Hz, 1H), 7.69 (d, *J* = 7.48,1H), 7.62 (d, *J* = 7.64 Hz, 1H), 7.31 (t, *J* = 7.12 Hz, 2H), 7.26 (t, *J* = 7.12 Hz, 2H), 6.91 (d, *J* = 15.4 Hz, 1H), 6.87 (d, *J* = 8.56 Hz, 2H), 6.73 (d, *J* = 8.64 Hz, 2H), 4.60 (t, *J* = 6.3 Hz, 2H), 3.68 (s, 3H), 3.08(q, *J* = 6.76 Hz, 2H), 2.57 (t, *J* = 6.24 Hz, 2H), 2.42 (t, *J* = 7.24 Hz, 2H). LC-MS *m*/*z*: 394.1 [M + H]^+^.

*(E)-3-(1-(3-((3,5-Dichlorophenyl)amino)-3-oxopropyl)-1H-benzo[d]imidazol-2-yl)acrylic acid* (**6n**): Compound **6n** was prepared from **4** and 3,5-dichloroaniline in a similar manner as compound **6a**. White solid. Yield 51.7%. m.p.: 152–154 °C. ^1^H-NMR (DMSO-*d*_6_) δ: 10.48 (s, 1H), 7.63 (d, *J* = 7.88 Hz, 2H), 7.60 (d, *J* = 15.3 Hz, 1H), 7.54 (d, *J* = 1.5 Hz, 2H), 7.26 (t, *J* = 7.0 Hz, 1H), 7.23 (s, 1H), 7.20 (t, *J* = 7.3 Hz, 1H), 6.92 (d, *J* = 15.3 Hz, 1H), 4.67 (t, *J* = 6.1 Hz, 2H), 2.86 (t, *J* = 6.3 Hz, 2H). LC-MS *m*/*z*: 404.0 [M + H]^+^.

*(E)-3-(1-(3-((2,4,6-Trimethylphenyl)amino)-3-oxopropyl)-1H-benzo[d]imidazol-2-yl)acrylic acid* (**6o**): Compound **6o** was prepared from **4** and 2,4,6-trimethylaniline in a similar manner as compound **6a**. White solid. Yield 55.5%. m.p.: 275–278 °C. ^1^H-NMR (DMSO-*d*_6_) δ: 9.17 (s, 1H), 7.73(d, *J* = 15.2 Hz, 1H), 7.65 (t, *J* = 8.3 Hz, 2H), 7.26 (dq, *J* = 7.2, 6.0 Hz, 2H), 6.93(d, *J* = 15.2 Hz, 1H), 6.76(s, 2H), 4.68 (t, *J* = 6.1 Hz, 2H), 2.88 (t, *J* = 6.2 Hz, 2H), 2.16 (s, 3H), 1.73 (s, 6H). ^13^C-NMR (DMSO-*d*_6_) δ 168.0, 167.2, 148.0, 142.8, 135.3, 135.3, 134.7, 132.1, 129.1, 128.1, 126.6, 123.2, 122.7, 119.4, 111.3, 40.1, 35.6, 20.4, 17.6. LC-MS *m*/*z*: 378.1 [M + H]^+^.

#### 3.1.2. General Procedure for the Synthesis of **7a**–**7c**

To a solution of compounds **6d**, **6f** or **6o** (0.29 mmol) in methanol (5 mL) was added Pd/C (5%, 31 mg, 0.14 mmol). The mixture was stirred and subjected to an atmosphere of hydrogen for 3 h at room temperature. After the start material was completed, the mixture was filtered and the filtrate was concentrated in vacuo to obtain the desired compounds **7a**–**7c**.

*3-(1-(3-Oxo-3-(p-tolylamino)propyl)-1H-benzo[d]imidazol-2-yl)propanoic acid* (**7a**): Compound **7a** was prepared from **6d** using the general procedure as a white solid. Yield 98.0%. m.p.: 66–68 °C. ^1^H-NMR (DMSO-*d*_6_) δ: 12.28 (s, 1H), 9.95 (s, 1H), 7.54 (dd, *J* = 11.4, 7.8 Hz, 2H), 7.38 (t, *J* = 11.6 Hz, 2H), 7.18 (t, *J* = 7.1 Hz, 1H), 7.13 (t, *J* = 7.5 Hz, 1H), 7.07 (d, *J* = 8.3 Hz, 2H), 4.51 (t, *J* = 6.8 Hz, 2H), 3.13 (t, *J* = 7.0 Hz, 2H), 2.82 (t, *J* = 6.9 Hz, 4H), 2.22 (s, 3H). LC-MS *m*/*z*: 352.1 [M + H]^+^.

*3-(1-(3-((4-Chlorophenyl)amino)-3-oxopropyl)-1H-benzo[d]imidazol-2-yl)propanoic acid* (**7b**): Compound **7b** was prepared from **6f** using the general procedure as a white solid. Yield 95.0%. m.p.: 66–68 °C. ^1^H-NMR (DMSO-*d*_6_) δ: 12.28 (s, 1H), 10.15 (s, 1H), 7.54 (m, 4H), 7.33 (d, *J* = 7.9 Hz, 2H), 7.18 (t, *J* = 7.5 Hz, 1H), 7.13 (t, *J* = 7.4 Hz, 1H), 4.51 (d, *J* = 6.0 Hz, 2H), 3.60 (d, *J* = 6.1Hz, 2H), 3.12 (d, *J* = 6.2 Hz, 2H), 2.84 (d, *J* = 6.7 Hz, 2H). LC-MS *m*/*z*: 372.1 [M + H] ^+^.

*3-(1-(3-(Mesitylamino)-3-oxopropyl)-1H-benzo[d]imidazol-2-yl)propanoic acid* (**7c**): Compound **7c** was prepared from **6o** using the general procedure as a white solid. Yield 94.0%. m.p.: 222–224 °C. ^1^H-NMR (DMSO-*d*_6_) δ: 12.25 (s, 1H), 9.19 (s, *J* = 8.1 Hz, 1H), 7.54 (dd, *J* = 7.3, 4.0 Hz, 2H), 7.21–7.10 (m, 2H), 6.77 (s, 2H), 4.50 (t, *J* = 6.6 Hz, 2H), 3.14 (t, *J* = 7.1 Hz, 2H), 2.86 (t, *J* = 6.6 Hz, 2H), 2.81 (t, *J* = 7.1 Hz, 2H), 2.17 (t, *J* = 4.3 Hz, 3H), 1.81 (s, 6H). LC-MS *m*/*z*: 380.2 [M + H]^+^.

#### 3.1.3. General Procedure for the Synthesis of **13a**–**13j**

*1H-Benzo[d]imidazole-2-carboxylic acid* (**8**): To a mixture of **1** (6.0 g, 40.5 mmol) and K_2_CO_3_ (4.3 g, 31.2 mmol) in 100 mL water was added KMnO_4_ (6.4 g, 40.5 mmol) in batches, the mixture was refluxed for 1h. After complete conversion of starting material, the reaction mixture was filtered; the pH of the filtrate was adjusted to 5 with 6N HCl at 0 °C. Filter and dried to obtain **8** as white solid in 45.0% yield. LC-MS *m*/*z*: 161.5 [M − H]^−^.

*1H-Benzo[d]imidazole-2-carboxamide* (**9**): A solution of **8** (3.0 g, 18.6 mmol) in SOCl_2_ (30 mL) was stirred at 70 °C for 4 h. The mixture was cooled to room temperature, concentrated in vacuo and aqueous ammonia (150 mL) was added. The resulting solution was stirred at 70 °C for 5 h, then cooled and water (50 mL) was added and stirred at room temperature for additional 1 h. Filtration and drying gave **9** as a yellow solid in 51.4% yield. LC-MS *m*/*z*: 162.1 [M + H]^+^.

*1H-Benzo[d]imidazole-2-carbothioamide* (**10**): To a solution of **9** (3.0 g, 18.6 mmol) in THF (70 mL) was added Lawesson’s reagent (7.5 g, 18.6 mmol). The resulting solution was stirred at 66 °C for 5 h. After complete conversion of the starting material, the reaction mixture was concentrated in vacuo and the residue was redissolved in DCM (500 mL), the solution was washed with saturated NaCl solution three times, dried over anhydrous sodium sulfate, concentrated and purified by flash silica gel column chromatography (DCM:MeOH = 100:1) to obtain **10** as yellow solid in 61.2% yield. LC-MS *m*/*z*: 178.2 [M + H]^+^.

*Ethyl 2-(1H-benzo[d]imidazol-2-yl)thiazole-4-carboxylate* (**11**): A solution of ethyl 3-bromo-2-oxopropanoate (3.0 g, 16.9 mmol) and **10** (3.0 g 16.9 mmol) in ethanol (150 mL) was stirred at 66 °C for 5 h. After complete conversion of the starting material, the reaction mixture was concentrated in vacuo, and then purified by silica gel column chromatography (DCM:MeOH= 250:1) to obtain **11** as white solid in 95.0% yield. LC-MS *m*/*z*: 274.3 [M + H]^+^.

*3-(2-(4-(Ethoxycarbonyl)thiazol-2-yl)-1H-benzo[d]imidazol-1-yl)propanoic acid* (**12**): To a solution of **11** (1.0 g, 3.70 mmol) in mixture of DMF (20 mL) and acetone (100 mL), 3-bromopropionic acid (2.2 g, 14.8 mmol) and a solution of K_2_CO_3_ (10.0 g, 72.4 mmol) in water (2 mL) was added, the mixture was stirred at 70 °C for 4 h. After complete conversion of the starting material, the reaction mixture was concentrated in vacuo and redissolved in water (80 mL). The resulting solution was acidified to pH 5 with 6 N HCl at 0 °C, and then extracted with ethyl acetate, the organic layers combined and washed with saturated NaCl and dried over anhydrous sodium sulfate, filtered, and concentrated in vacuo to give **12** as white solid in 65.0% yield. LC-MS *m*/*z*: 344.1 [M − H]^−^.

*2-(1-(3-Oxo-3-(phenylamino)propyl)-1H-benzo[d]imidazol-2-yl)thiazole-4-carboxylic acid* (**13a**): To a mixture of compounds **12** (500 mg, 1.45 mmol), 4-methylmorpholine (0.8 mL, 4.25 mmol) and aniline (161 mg, 1.74 mmol) in dried DCM was added HOBt (389 mg, 2.90 mmol) at 0 °C, and then EDCI (557 mg, 2.90 mmol) was added in batches. The resulting solution was warmed to room temperature after stirring for 30 min at 0 °C. The reaction mixture was washed with water three times and saturated NaCl solution once. The organic layer was dried over anhydrous sodium sulfate, concentrated and purified by flash silica gel column chromatography (PE/EA = 4/1, *v*/*v*), and then the residue was re-dissolved in THF (15 mL), and lithium hydroxide solution (3.5 mmol/mL, 5 mL) was added dropwise. The resulting solution was stirred at room temperature for 6 h. After the reaction was completed, the reaction mixture was concentrated in vacuo, and the residue dissolved in water and acidified with 2 N HCl to pH 5 at 0 °C, filtered and dried to obtain the desired compound **13a** as a white solid in 56.1% yield. m.p.: 226–228 °C. ^1^H-NMR (DMSO-*d*_6_) δ: 13.23 (s, 1H), 9.88 (s, 1H), 8.64 (s, 1H), 7.77 (d, *J* = 8.2 Hz, 1H), 7.73 (d, *J* = 8.0 Hz, 1H), 7.43 (d, *J* = 7.9 Hz, 2H), 7.36 (t, *J* = 7.5 Hz, 1H), 7.29 (t, *J* = 7.5 Hz, 1H), 7.22 (t, *J* = 7.8 Hz, 2H), 6.99 (t, *J* = 7.3 Hz, 1H), 5.15 (t, *J* = 6.6 Hz, 2H), 2.96 (t, *J* = 6.6 Hz, 2H). ^13^C-NMR (DMSO-*d*_6_) δ 168.7, 161.9, 159.3, 148.6, 144.1, 142.0, 138.8, 136.1, 130.8, 128.6, 124.1, 123.2, 123.1, 119. 6, 119.2, 111.7, 41.3, 36.8. LC-MS *m*/*z*: 391.0 [M − H]^−^.

*2-(1-(3-((3-Bromophenyl)amino)-3-oxopropyl)-1H-benzo[d]imidazol-2-yl)thiazole-4-carboxylic acid* (**13b**): Compound **13b** was prepared from **12** and 3-bromoaniline in a similar manner as compound **13a**. White solid. Yield 53.0%. m.p.: 210–212 °C. ^1^H-NMR (DMSO-*d*_6_) δ: 9.59 (s, 1H), 8.62 (s, 1H), 7.74 (d, *J* = 8.1 Hz, 3H), 7.58 (d, *J* = 7.9 Hz, 1H), 7.37 (t, *J* = 7.4 Hz, 1H), 7.31 (t, *J* = 7.5 Hz, 2H), 7.27 (s, 1H), 7.09 (t, *J* = 7.1 Hz, 1H), 5.15 (t, *J* = 6.2 Hz, 2H), 3.02 (s, 2H). LC-MS *m*/*z*: 469.4 [M − H]^−^.

*2-(1-(3-((2-Bbromophenyl)amino)-3-oxopropyl)-1H-benzo[d]imidazol-2-yl)thiazole-4-carboxylic acid* (**13c**): Compound **13c** was prepared from **12** and 2-bromoaniline in a similar manner as compound **13a**. White solid. Yield 48.3%. m.p.: 186–188 °C. ^1^H-NMR (DMSO-*d*_6_) δ: 9.59 (s, 1H), 8.62 (s, 1H), 7.74 (d, *J* = 8.1 Hz, 3H), 7.58 (d, *J* = 7.9 Hz, 1H), 7.37 (t, *J* = 7.4 Hz, 1H), 7.31 (t, *J* = 7.5 Hz, 2H), 7.27 (s, 1H), 7.09 (t, *J* = 7.1 Hz, 1H), 5.15 (t, *J* = 6.2 Hz, 2H), 3.02 (s, 2H). LC-MS *m*/*z*: 469.4 [M − H]^−^.

*2-(1-(3-((3-Methoxyphenyl)amino)-3-oxopropyl)-1H-benzo[d]imidazol-2-yl)thiazole-4-carboxylic acid* (**13d**): Compound **13d** was prepared from **12** and 3-methoxyaniline in a similar manner as compound **13a**. White solid. Yield 46.8%. m.p.: 223–225 °C. ^1^H-NMR (DMSO-*d*_6_) δ: 9.92 (s, 1H), 8.62 (s, 1H), 7.77 (d, *J* = 8.2 Hz, 1H), 7.73 (d, *J* = 8.0 Hz, 1H), 7.36 (t, *J* = 7.7 Hz, 1H), 7.29 (t, *J* = 7.6 Hz, 1H), 7.13 (dd, *J* = 9.6, 6.6 Hz, 2H), 6.99 (d, *J* = 8.0 Hz, 1H), 6.58 (d, *J* = 8.2 Hz, 1H), 6.58 (d, *J* = 8.2 Hz, 1H), 5.14 (t, *J* = 6.7 Hz, 2H), 3.68 (s, 3H), 2.95 (t, *J* = 6.7 Hz, 2H). ^13^C-NMR (DMSO-*d*_6_) δ 169.0, 164.1, 159.4, 159.3, 156.1, 145.2, 142.1, 140.6, 135.9, 129.1, 123.8, 123.6, 122.8, 119.5, 112.5, 110.9, 108.7, 106.1, 55.0, 42.6, 38.2. LC-MS *m*/*z*: 421.3 [M − H]^−^.

*2-(1-(3-((3,5-Dimethoxyphenyl)amino)-3-oxopropyl)-1H-benzo[d]imidazol-2-yl)thiazole-4-carboxylic acid* (**13e**): Compound **13e** was prepared from **12** and 3,5-dimethoxyaniline in a similar manner as compound **13a**. White solid. Yield 43.9%. m.p.: 208–210 °C. ^1^H-NMR (DMSO-*d*_6_) δ: 10.68 (s, 1H), 8.02 (s, 1H), 7.72 (d, *J* = 10.5 Hz, 2H), 7.35 (t, *J* = 7.5 Hz, 1H), 7.28 (t, *J* = 7.6 Hz, 1H), 7.10 (s, 2H), 6.19 (s, 1H), 5.04–4.87 (m, 2H), 3.70 (s, 6H), 3.13–2.97 (m, 2H). ^13^C-NMR (DMSO-*d*_6_) δ 169.3, 164.3, 160.4, 159.7, 156.3, 145.4, 142.3, 141.2, 136.1, 124.0, 123.8, 123.1, 119.7, 111.2, 98.8, 95.5, 55.3, 42.7, 38.4. LC-MS *m*/*z*: 451.0 [M − H]^−^.

*2-(1-(3-((3-Nitrophenyl)amino)-3-oxopropyl)-1H-benzo[d]imidazol-2-yl)thiazole-4-carboxylic acid* (**13f**): Compound **13f** was prepared from **12** and 3-nitroaniline in a similar manner as compound **13a**. White solid. Yield 44.7%. m.p.: 234–236 °C. ^1^H-NMR (DMSO-*d*_6_) δ: 11.64 (s, 1H), 8.92 (s, 1H), 8.30 (d, *J* = 8.0 Hz, 1H), 8.06 (s, 1H), 7.91 (d, *J* = 8.1 Hz, 1H), 7.73 (d, *J* = 8.0 Hz, 2H), 7.59 (t, *J* = 8.1 Hz, 1H), 7.37 (t, *J* = 7.6 Hz, 1H), 7.30 (t, *J* = 7.6 Hz, 1H), 5.01–4.93 (m, 2H), 3.18–3.12 (m, 2H). LC-MS *m*/*z*: 436.4 [M − H]^−^.

*2-(1-(3-Oxo-3-((3-(trifluoromethyl)phenyl)amino)propyl)-1H-benzo[d]imidazol-2-yl)thiazole-4-carboxylic acid (***13g**): Compound **13g** was prepared from **12** and 3-(trifluoromethyl)aniline in a similar manner as compound **13a**. White solid. Yield 47.6%. m.p.: 178–180 °C. ^1^H-NMR (400 MHz, DMSO-*d*_6_) δ: 11.35 (s, 1H), 8.36 (s, 1H), 8.11 (d, *J* = 7.9 Hz, 1H), 8.05 (s, 1H), 7.72 (dd, *J* = 7.7, 4.1 Hz, 2H), 7.52 (t, *J* = 7.8 Hz, 1H), 7.38 (d, *J* = 7.9 Hz, 1H), 7.36 (d, *J* = 7.8 Hz, 1H), 7.29 (t, *J* = 7.5 Hz, 1H), 4.98–4.94 (m, 2H), 3.13 (t, *J* = 13.1 Hz, 2H). ^13^C-NMR (150 MHz, DMSO) δ 169.4, 162.1, 159.2, 144.3, 142.1, 139.6, 136.1, 130.3, 129.9, 129.5, 129.2, 125.1, 124.2, 123.3, 123.2, 122.9, 119.7, 115.4, 115.4, 111.6, 41.4, 37.1. LC-MS *m*/*z*: 459.1 [M − H]^−^.

*2-(1-(3-Oxo-3-((4-(trifluoromethyl)phenyl)amino)propyl)-1H-benzo[d]imidazol-2-yl)thiazole-4-carboxylic acid* (**13h**): Compound **13h** was prepared from **12** and 4-(trifluoromethyl)aniline in a similar manner as compound **13a**. White solid. Yield 56.7%. m.p.: 176–178 °C. ^1^H-NMR (DMSO-*d*_6_) δ: 11.36 (s, 1H), 8.13 (d, *J* = 8.1 Hz, 2H), 8.04 (s, 1H), 7.72 (d, *J* = 8.3 Hz, 2H), 7.64 (d, *J* = 8.5 Hz, 2H), 7.36 (t, *J* = 7.6 Hz, 1H), 7.29 (t, *J* = 7.6 Hz, 1H), 4.98–4.92 (m, 2H), 3.23-3.09 (m, 2H). ^13^C-NMR (DMSO-*d*_6_) δ 168.8, 161.2, 158.7, 147.8, 143.5, 141.8, 141.4, 135.5, 130.2, 125.3, 124.7, 123.5, 122.9, 122.6, 122.5, 119.0, 118.3, 111.0, 40.5, 36.3. LC-MS *m*/*z*: 459.1 [M − H]^−^.

*2-(1-(3-((3,5-Dichlorophenyl)amino)-3-oxopropyl)-1H-benzo[d]imidazol-2-yl)thiazole-4-carboxylic acid* (**13i**): Compound **13i** was prepared from **12** and 3,5-dichloroaniline in a similar manner as compound **13a**. White solid. Yield 42.6%. m.p.: 192–194 °C. ^1^H-NMR (DMSO-*d*_6_) δ: 10.42 (s, 1H), 8.62 (s, 1H), 7.77 (d, *J* = 8.2 Hz, 1H), 7.74 (d, *J* = 8.0 Hz, 1H), 7.47 (d, *J* = 1.8 Hz, 2H), 7.37 (dd, *J* = 10.0, 5.3 Hz, 1H), 7.32–7.30 (m, 1H), 7.20 (t, *J* = 1.8 Hz, 1H), 5.18 (t, *J* = 6.6 Hz, 2H), 2.97 (t, *J* = 6.6 Hz, 2H). ^13^C-NMR (DMSO-*d*_6_) δ 168.9, 161.1, 158.7, 147.8, 143.5, 141.3, 140.5, 135.4, 133.3, 130.1, 123.5, 122.5, 121.7, 118.9, 116.6, 111.0, 40.5, 36.3. LC-MS *m*/*z*: 459.3 [M − H]^−^.

*2-(1-(3-((3-Fluoro-4-methylphenyl)amino)-3-oxopropyl)-1H-benzo[d]imidazol-2-yl)thiazole-4-carboxylic acid* (**13j**): Compound **13j** was prepared from **12** and 3-fluoro-4-methylaniline in a similar manner as compound **13a**. White solid. Yield 46.6%. m.p.: 204–206 °C. ^1^H-NMR (DMSO-d_6_) δ: 11.08 (s, 1H), 8.03 (s, 1H), 7.80 (d, *J* = 12.7 Hz, 1H), 7.74-7.65 (m, 2H), 7.61 (d, *J* = 8.3 Hz, 1H), 7.36 (t, *J* = 7.6 Hz, 1H), 7.29 (t, *J* = 7.6 Hz, 1H), 7.16 (t, *J* = 8.6 Hz, 1H), 5.05–4.87 (m, 2H), 3.14–2.96 (m, 2H), 2.18 (s, 3H). ^13^C-NMR (DMSO-d_6_) δ 168.9, 162.3, 160.9, 159.3, 144.3, 142.0, 138.4, 136.0, 131.2, 124.0, 123.0, 119.6, 118.4, 118.3, 115.0, 111.4, 106.2, 106.0, 41.6, 37.2, 13.7. LC-MS *m*/*z*: 423.4 [M − H]^−^.

### 3.2. Pin1 Inhibition Assay

Pin1 inhibitory activities were measured at 10 °C by protease-coupled assay as reported previous [24]. Suc-Ala-Glu-*cis*-Pro-Phe-4-nitroaniline (Bachem, Bubendorf, Switzerland) in 0.47 mol/L, LiCl/trifluoroethanol was used as the substrate. The assay buffer (860 μL of 35 mM HERES at pH 7.8), Pin1 (20 μL, Bought from Sino Biological Inc., Beijing, China) and target compounds (10 μL of varying comcentrations in DMSO) were pre-equilibtated in the cuvette at 10 °C for 30 min. Then, 150 uL of ice-cooled chymotrypsin (60 mg/mL in 0.001 M/HCl) was added and mixed immediately. Additional substrate (40 μL) was added to start the assay and the reaction was monitored by absorbance at 390 nm for 90 s. For each compound, four concentrations (10 μM, 3.3 μM, 1.1 μM, 0.37 μM) were chosen, and the assay was performed in duplicate. The data was analyzed by Graphpad Prism 6.0.

### 3.3. In Vitro Anti-Proliferative Assay

The tested cells were plated in 96-well microtiter plates with a density of 2,000 cells/well and incubated in a humidified atmosphere with 5% CO_2_ at 37 °C for 24 h. The cells were then exposed to tested compounds of different concentrations in triplicate. After incubated for 96 h, 50 μL of 3-(4,5-dimethylthiazol-2-yl)-2,5-diphenyltetrazolium bromide (MTT) solution (2 mg/mL) was added to each well and incubated for an additional 4 h. Then the suspernatant was discarded and the fromazan was dissolved in 200 μL of dimethyl sulfoxide (DMSO). The plate was oscillated subtly at room temperature for 10 min before the absorbance was measured at 570 nm by a microplate reader. Cell inhibitory ratio was calculated with the following formula:

Inhibitory Rate (%) = [(Acontrol − Atreated)/Acontrol] × 100%(1)

The GI_50_ was determined as the concentration that caused half inhibition of cell proliferation. All experiments were performed three times independently, and the results were reported presented as the mean.

## 4. Conclusions

We report herein the design and synthesis of some novel benzimidazole derivatives as potent Pin1 inhibitors. Among the 28 compounds, **6h** and **13g** were the most potent Pin1 inhibitors, with IC_50_ values of 0.64 and 0.37 μM, respectively. In vitro antiproliferative assays demonstrated that compounds **6d**, **6g**, **6h**, **6n**, **6o** and **7c** exhibited moderate antiproliferative activity against human prostate cancer PC-3 cells with GI_50_ values between 9.89 to 28.57 μM. This unique benzimidazole scaffold demonstrated great potential to be further explored as a source of potent Pin1 inhibitors with improved potency. Efforts are ongoing to further optimize and provide a systematic structure-activity analysis of the active positions and the results will be reported in due course.

## Figures and Tables

**Table 1:**
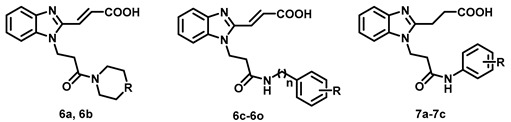
The structure and inhibitory activity against Pin1 of **6a**–**6o** and **7a**–**7c**.

Compd.	R	n	%Inhibition (10 μM) ^a^	IC_50_ (μM) ^b^	Compd.	R	n	%Inhibition (10 μM) ^a^	IC_50_ (μM) ^b^
**6a**	CH_2_	-	28	n.d. ^c^	**6j**	4-Br	1	59	n.d.
**6b**	O	-	27	n.d.	**6k**	4-Br	2	62	n.d.
**6c**	-H	0	46	n.d.	**6l**	4-OCH_3_	1	42	n.d.
**6d**	4-CH_3_	0	75	1.65	**6m**	4-OCH_3_	2	49	n.d.
**6e**	4-OCH_3_	0	48	n.d.	**6n**	3,5-diCl	0	71	1.87
**6f**	4-Cl	0	54	n.d.	**6o**	2,4,6-triCH_3_	0	84	0.97
**6g**	4-Br	0	66	1.73	**7a**	4-CH_3_	-	60	n.d.
**6h**	3-Br	0	90	0.64	**7b**	4-Cl	-	31	n.d.
**6i**	3,5-diOCH_3_	0	60	n.d.	**7c**	2,4,6-triCH_3_	-	60	n.d.
Juglone	-	-	-	10.01					

^a^ Values are means of triple independent experiments. ^b^ All results represent the mean of duplicate experiments. ^c^ not determined.

**Table 2:**
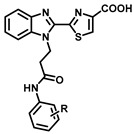
The structure and inhibitory activity against Pin1 of **13a**–**13j**.

Compd.	R	%Inhibition (10 μM) ^a^	IC_50_ (μM) ^b^	Compd.	R	%Inhibition (10 μM) ^a^	IC_50_ (μM) ^b^
**13a**	-H	73	n.d. ^c^	**13f**	3-NO_2_	40	n.d.
**13b**	3-Br	87	0.89	**13g**	3-CF_3_	99	0.37
**13c**	2-Br	79	n.d.	**13h**	4-CF_3_	81	1.15
**13d**	3-OCH_3_	75	n.d.	**13i**	3,5-diCl	80	1.35
**13e**	3,5-diOCH_3_	79	n.d.	**13j**	3-F-4-CH_3_	72	n.d.
Juglone	-	-	10.01				

^a^ Values are means of triple independent experiments. ^b^ All results represent the mean of duplicate experiments. ^c^ not determined.

**Table 3:** The antiproliferative activities against prostate cancer PC-3 cells and calculated PSA of target compounds.

Compound	PSA ^a^	GI_50_ (μM) ^b^	Compound	PSA ^a^	GI_50_ (μM) ^b^
**6a**	137.55	>50	**6o**	148.69	11.10
**6b**	153.66	>50	**7a**	141.44	>50
**6c**	151.52	>50	**7b**	141.06	>50
**6d**	156.07	17.42	**7c**	132.98	28.57
**6e**	168.82	>50	**13a**	212.54	>50
**6f**	157.60	>50	**13b**	212.13	>50
**6g**	155.77	14.10	**13c**	210.69	>50
**6h**	149.97	9.89	**13d**	231.02	>50
**6i**	180.56	>50	**13e**	225.21	>50
**6j**	149.90	>50	**13f**	296.88	>50
**6k**	153.04	>50	**13g**	212.06	>50
**6l**	161.52	>50	**13h**	212.13	>50
**6m**	173.11	>50	**13i**	211.37	>50
**6n**	152.21	20.32	**13j**	210.25	>50
Juglone	n.d. ^c^	4.89			

^a^ PSA was calculated with SYBYL software under the optimal conformation of the compounds. ^b^ value was the mean of triple experiments. ^c^ Not determined.

## Supplementary Materials

The following are available online at https://www.mdpi.com/1420-3049/24/7/1198/s1. ^1^H-NMR, ^13^C-NMR, MS of the target compounds.
