# Supplementary material for: Structure-Based Design of Novel Benzimidazole Derivatives as Pin1 Inhibitors
Source: Molecules. 2019 Mar 27;24(7):1198. doi: 10.3390/molecules24071198 (PMC6479814; doi:10.3390/molecules24071198)
Supplement: Supplementary file 1 [file molecules-24-01198-s001.pdf]

Supplementary Materials

Structure-Based Design of novel benzimidazole derivatives as Pin1 inhibitors

Shuxiang Wang, Lihong Guan, Jie Zang, Kun Xing, Jian Zhang, Dan Liu * and Linxiang Zhao *

Key Laboratory of Structure-Based Drug Design & Discovery of Ministry of Education, Shenyang Pharmaceutical University, Shenyang 110016, China; wangshuxiang10@163.com (S.W.); guanguan0919@163.com (L.G.); 15275312863@163.com (J.Z.); 15041472352@163.com (K.X.); ZJ2470703425@163.com (J.Z.)

* Correspondence: sammyld@163.com (D.L.); linxiang.zhao@vip.sina.com (L.Z.); Tel.: +024-4352-0221 (L.Z.)

1. Spectrum of target compounds

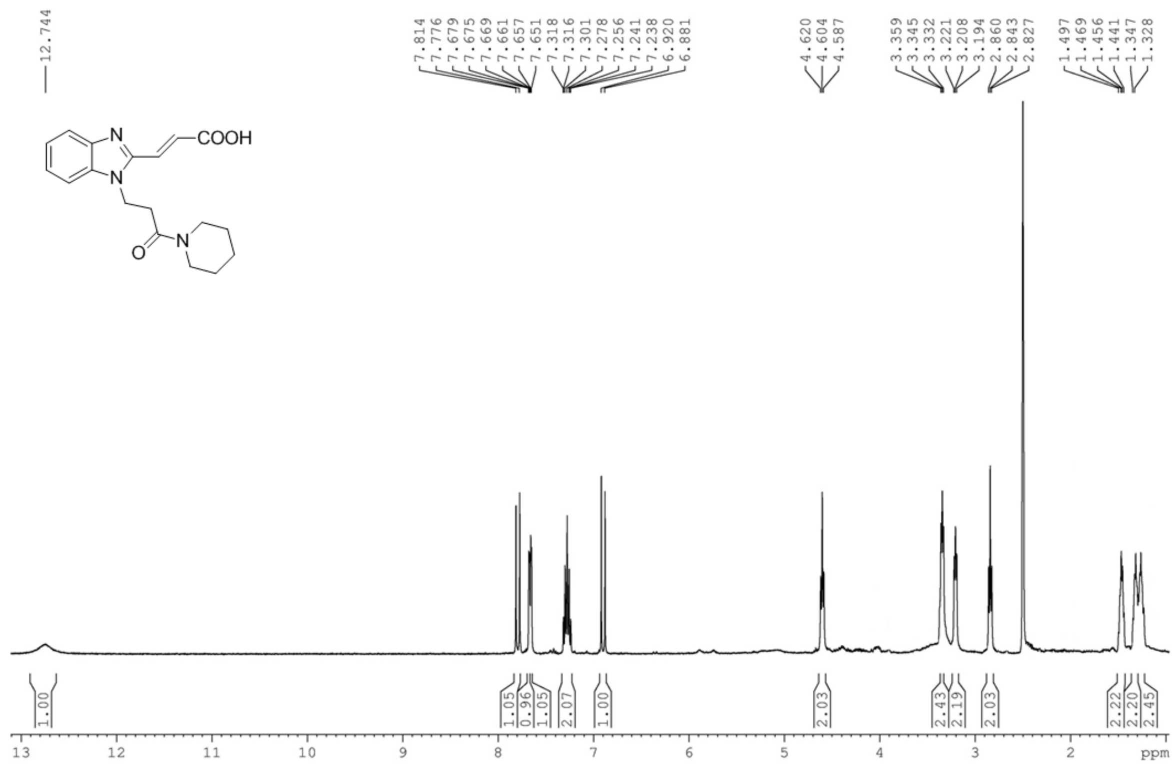

^{1}H-NMR spectra of 6a

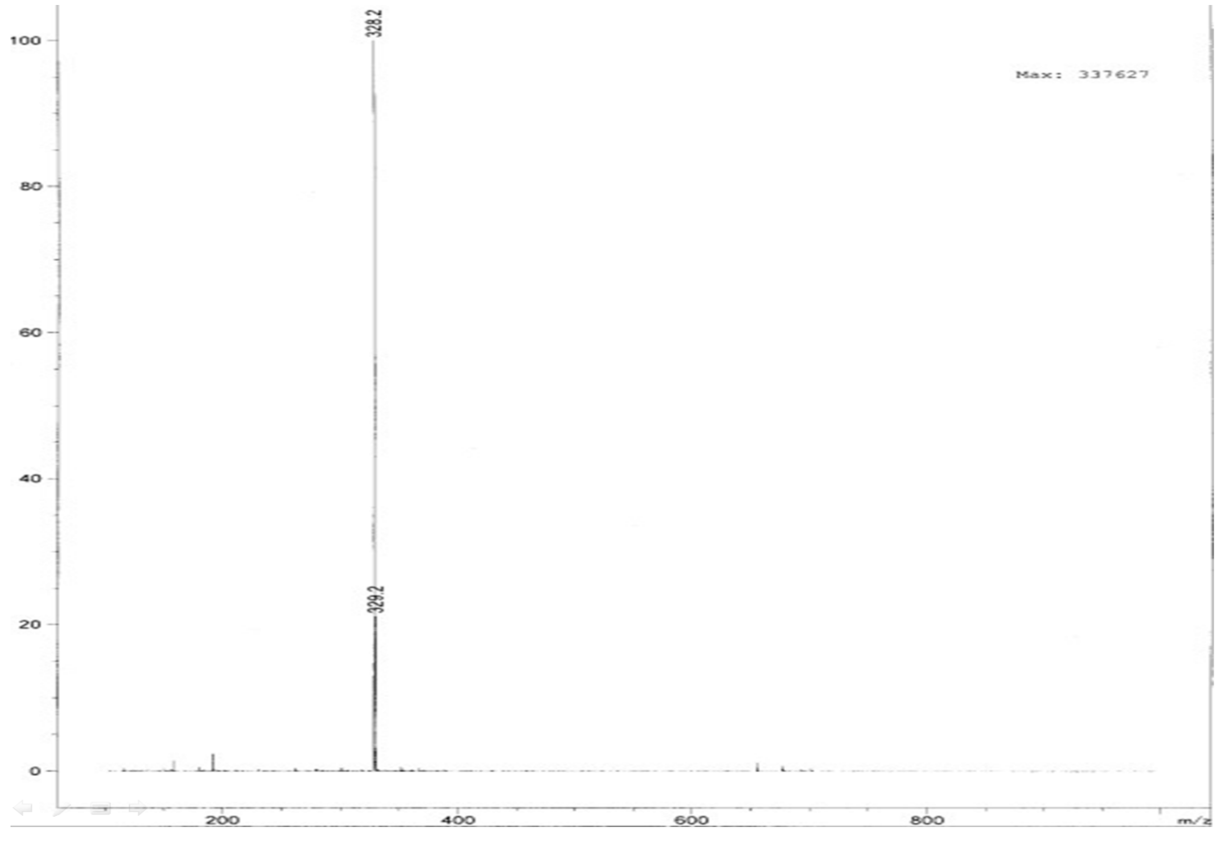

MS spectra of 6a

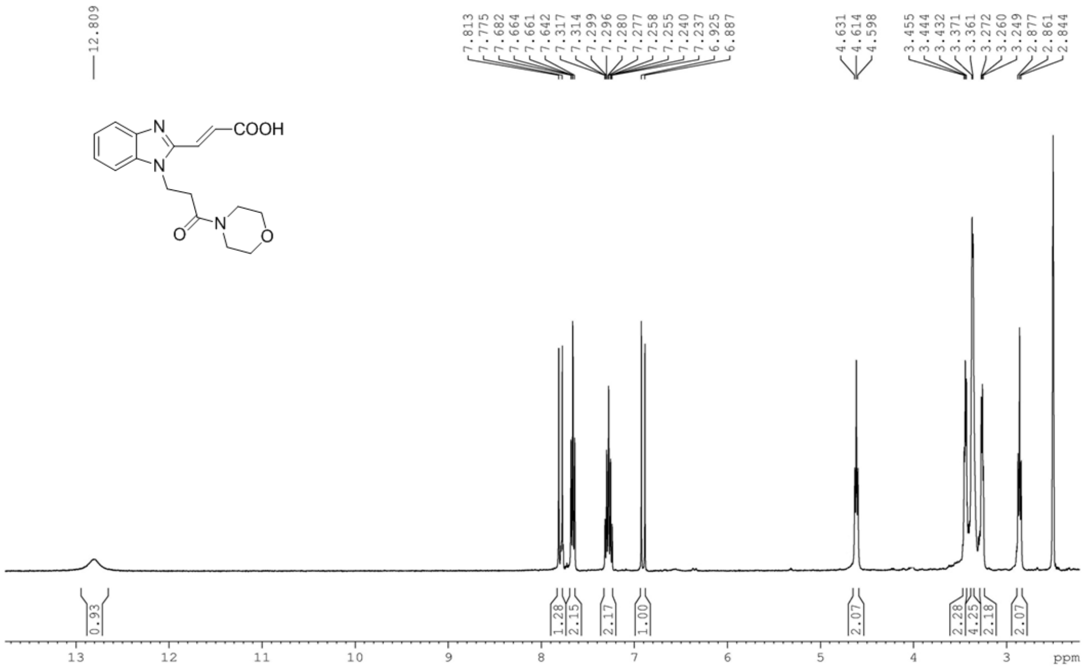

¹H-NMR spectra of 6b

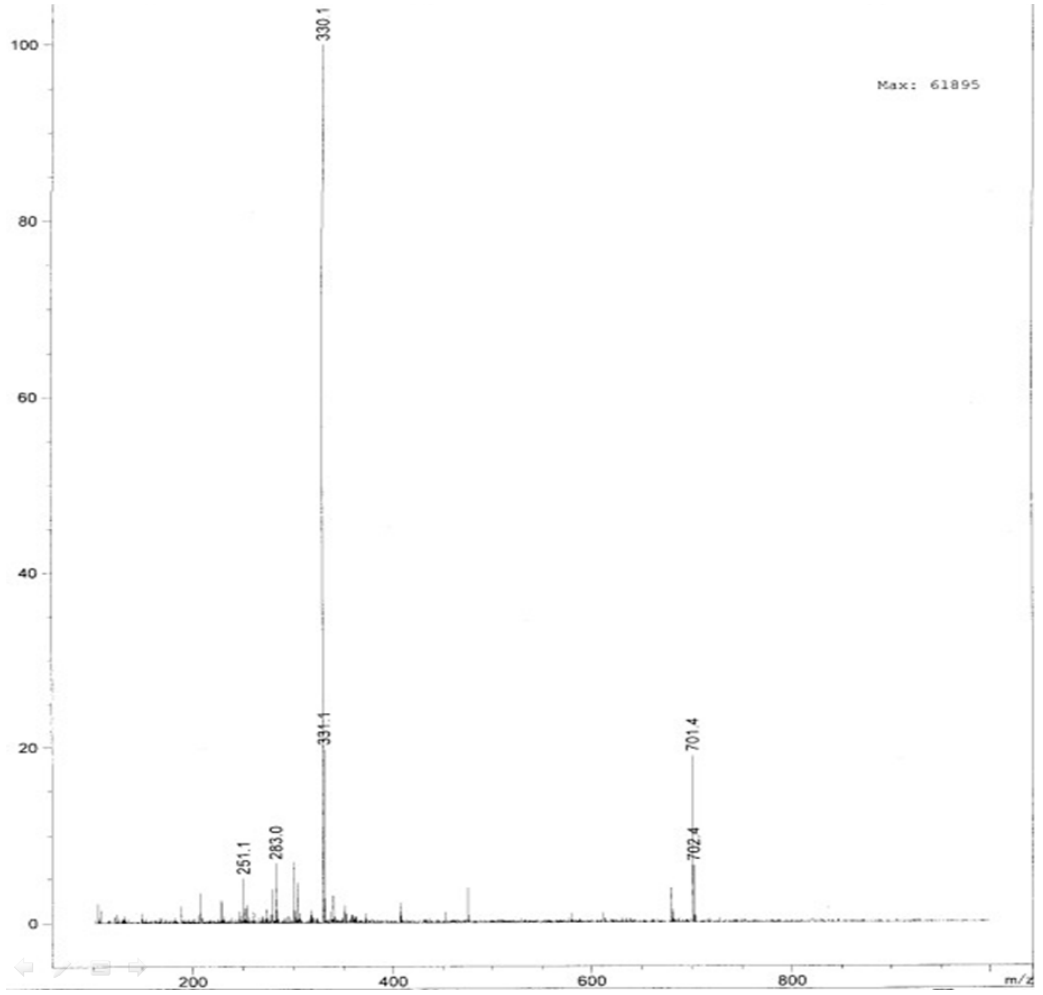

MS spectra of 6b

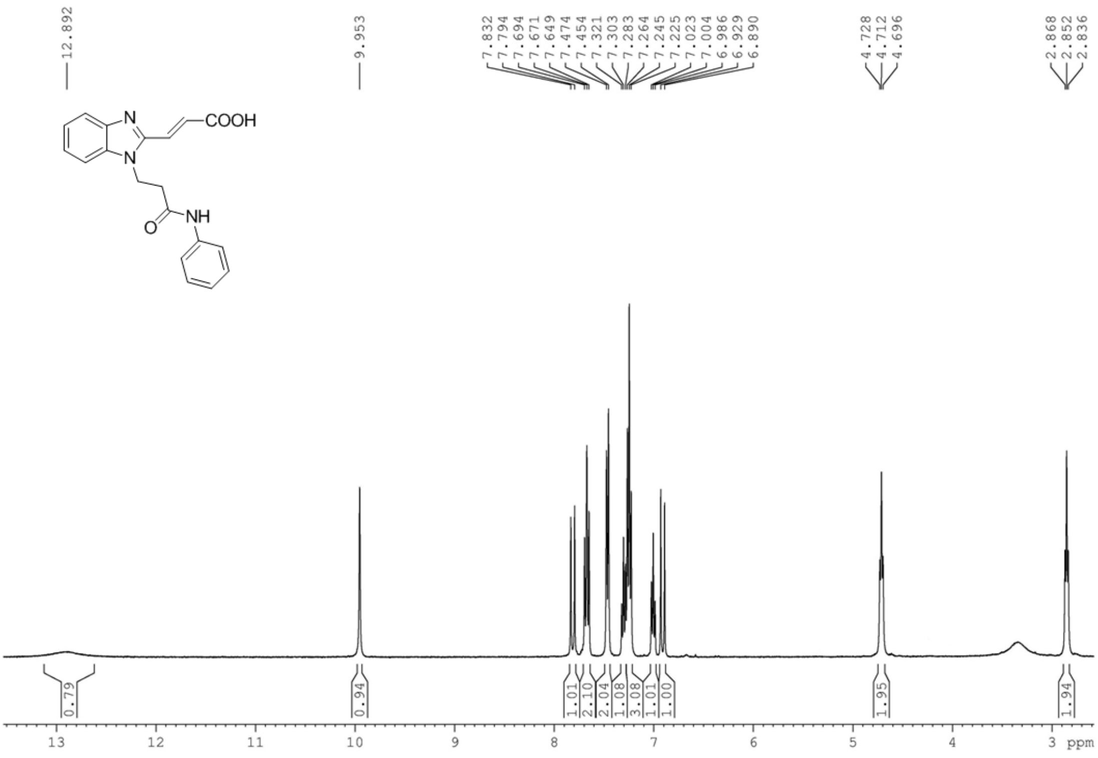

^{1}H-NMR spectra of 6c

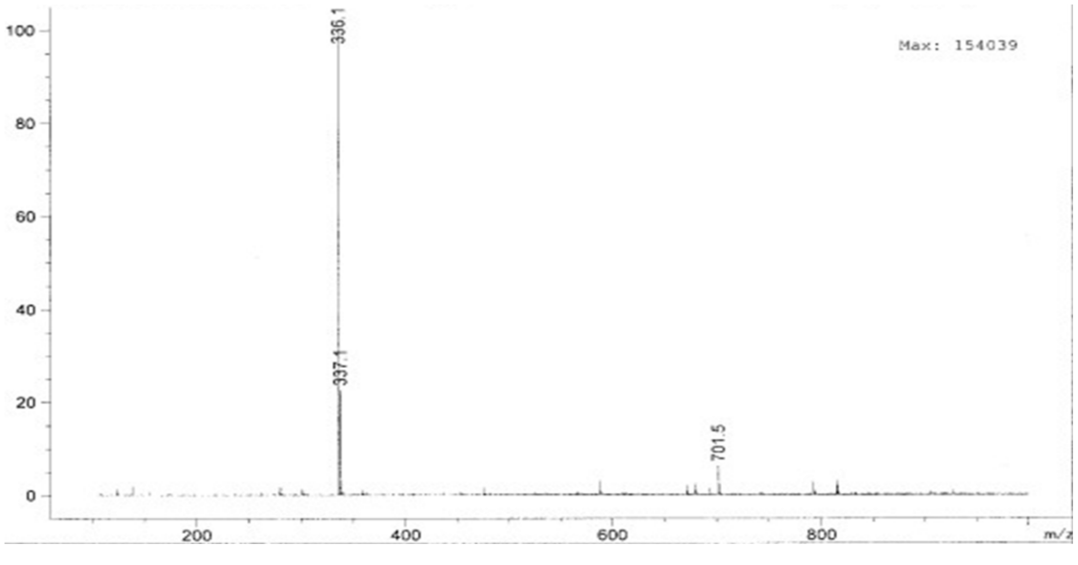

MS spectra of 6c

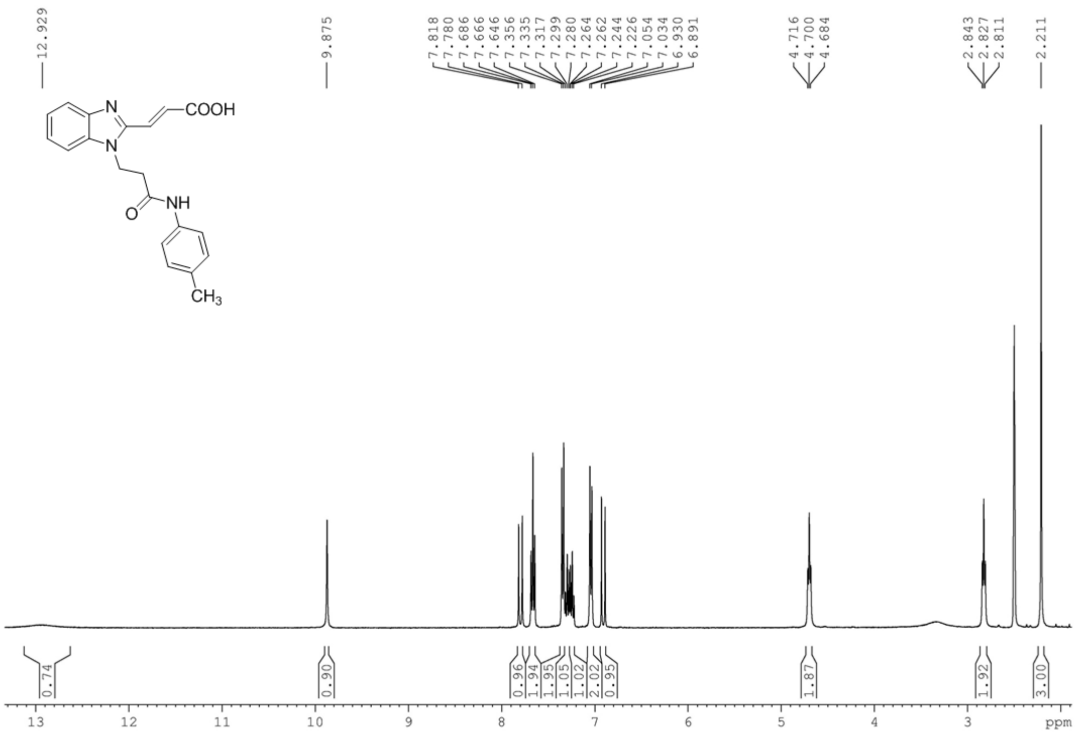

^{1}H-NMR spectra of 6d

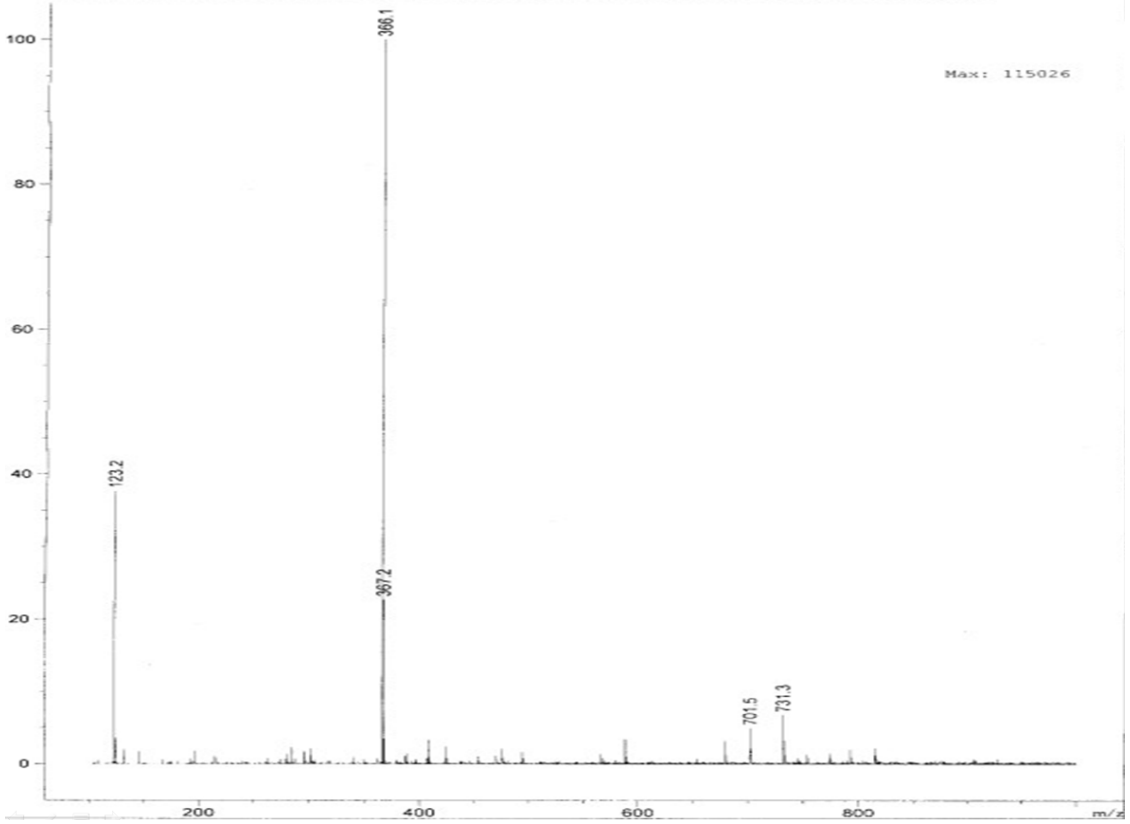

MS spectra of 6d

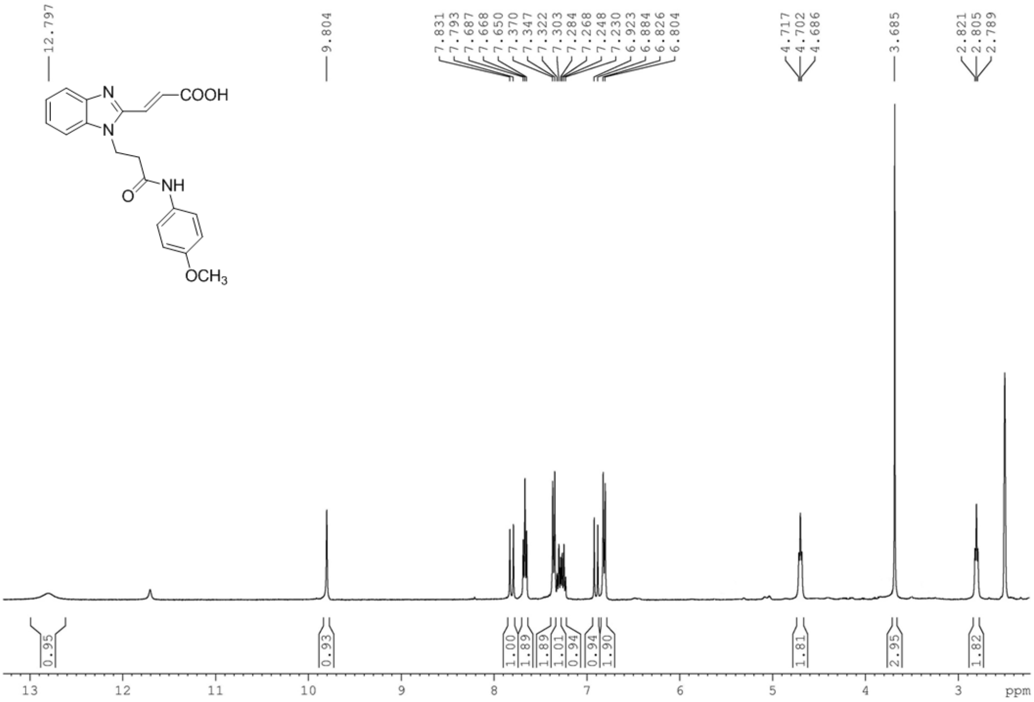

^{1}H-NMR spectra of 6e

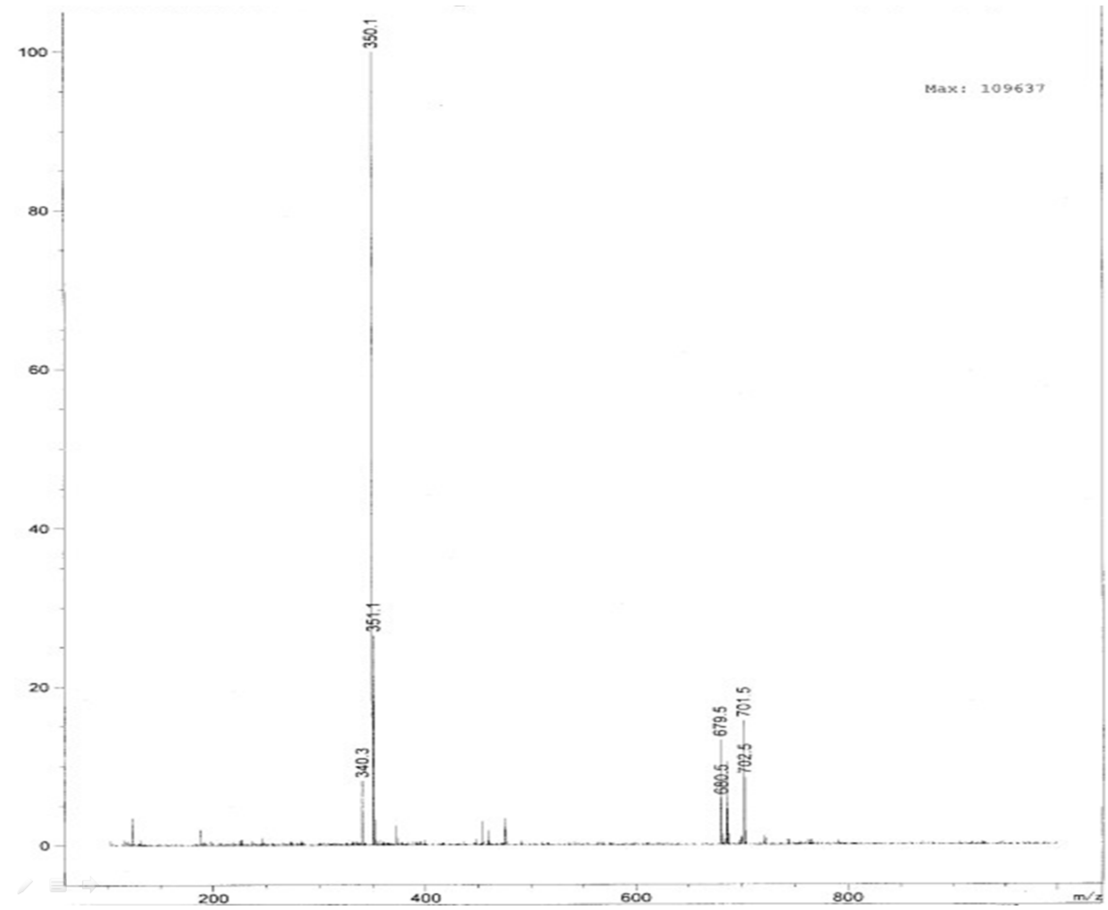

MS spectra of 6e

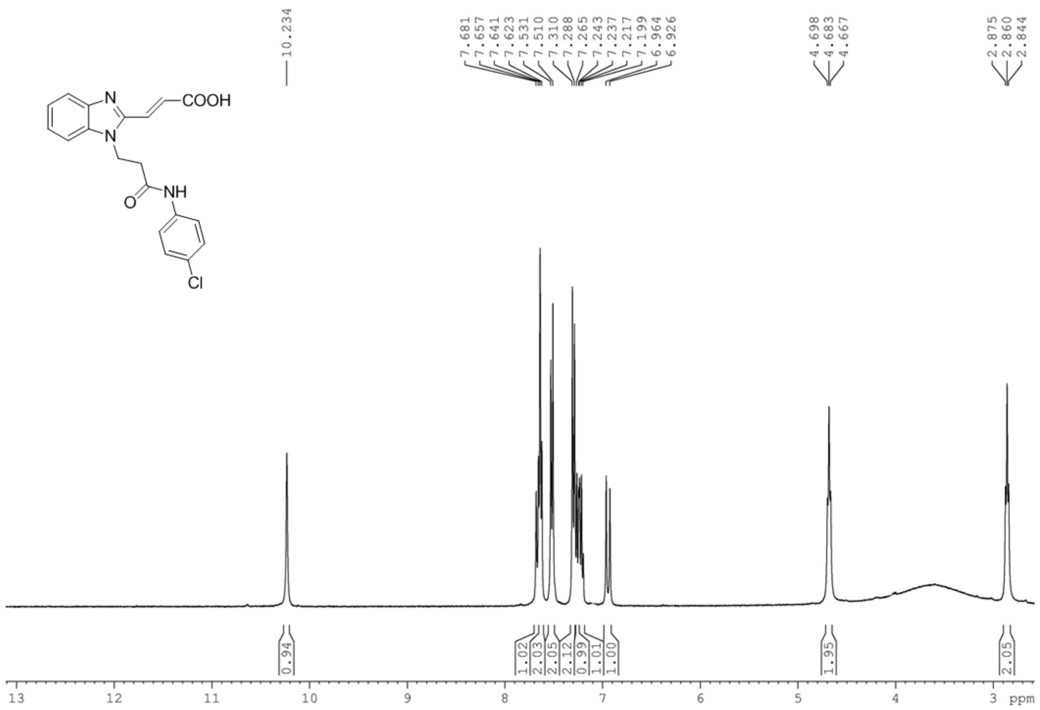

¹H-NMR spectra of 6f

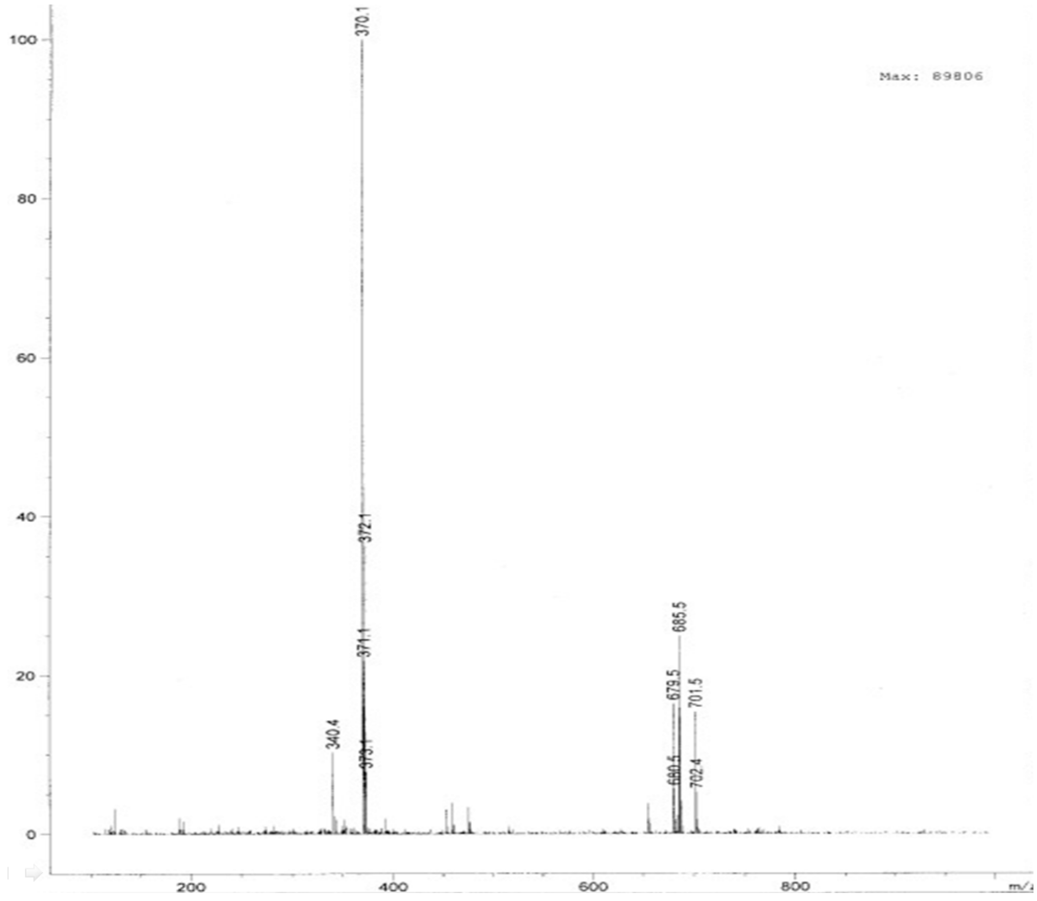

MS spectra of 6f

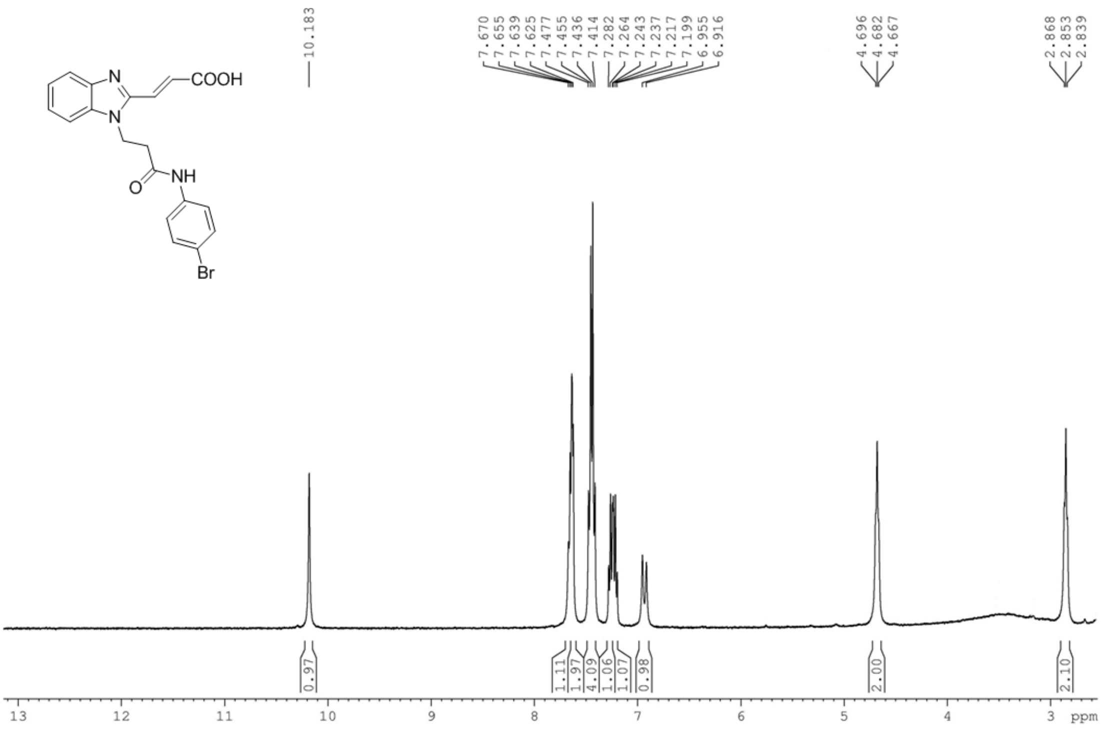

^{1}H-NMR spectra of 6g

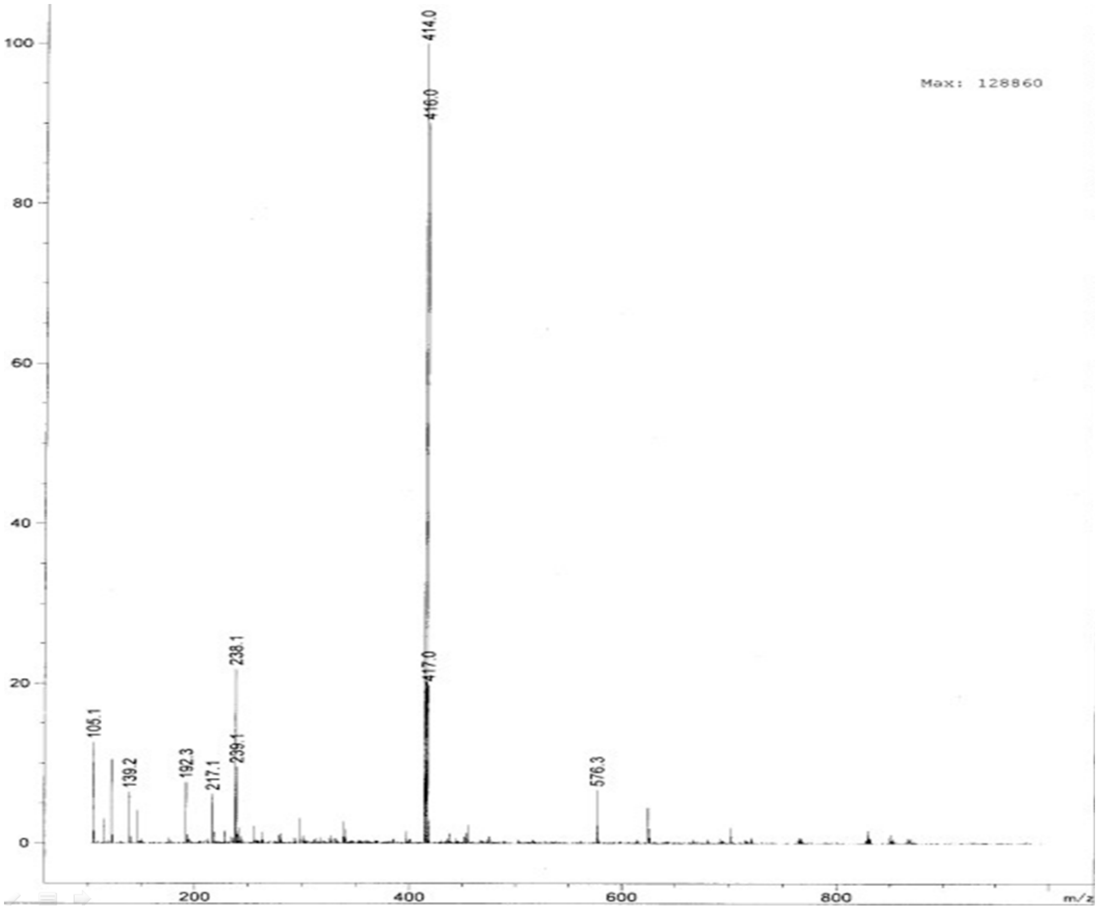

MS spectra of 6g

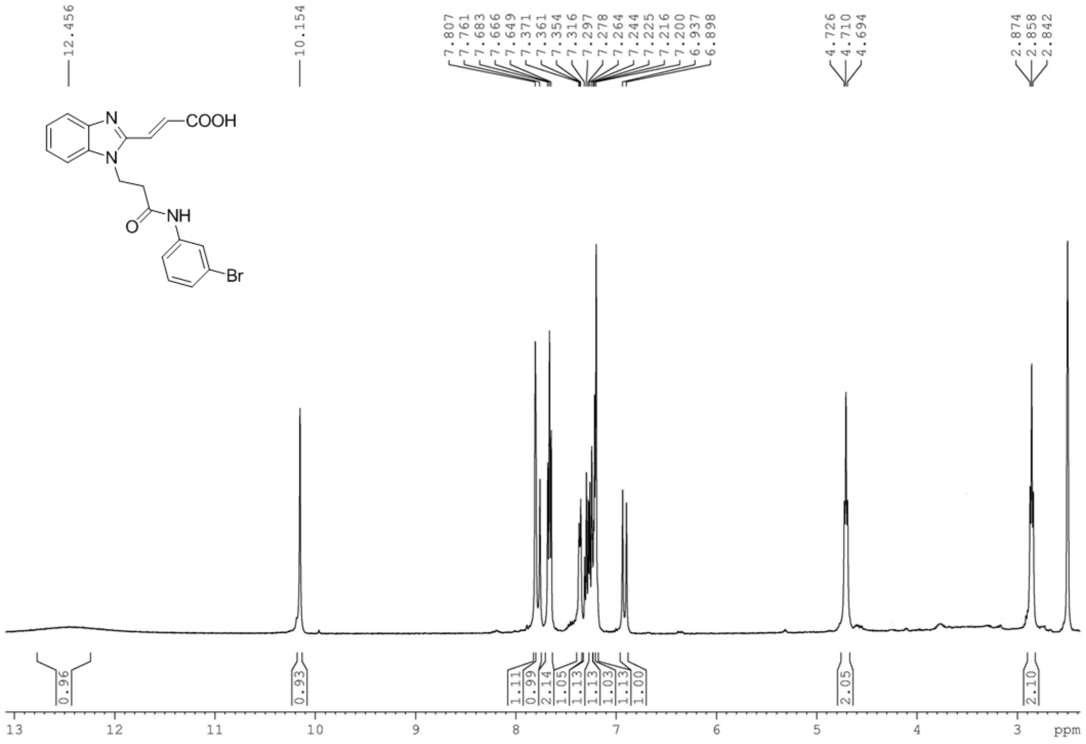

^{1}H-NMR spectra of 6h

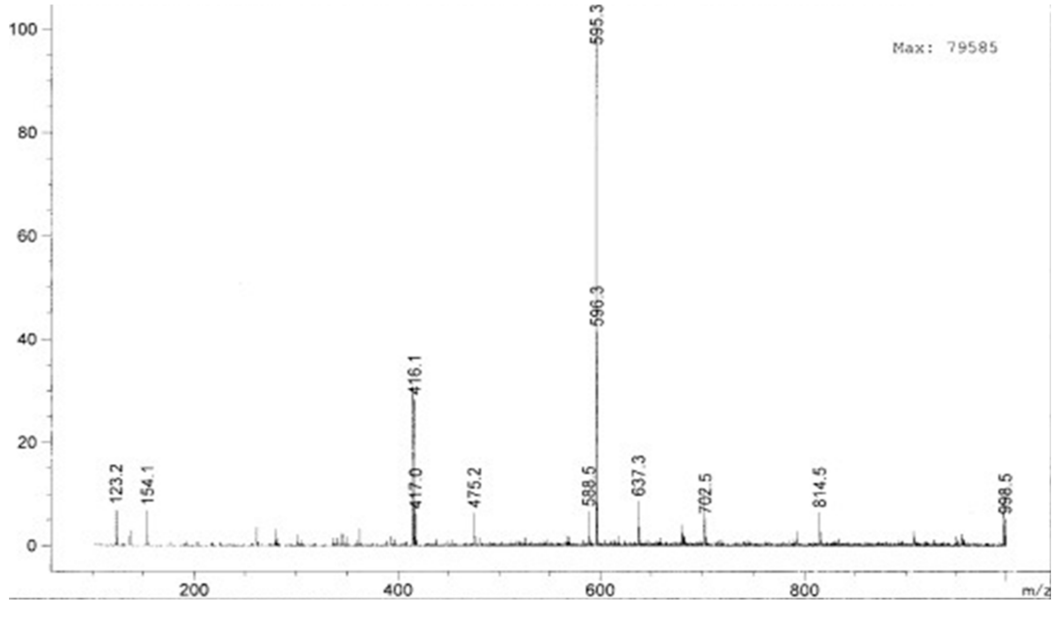

MS spectra of 6h

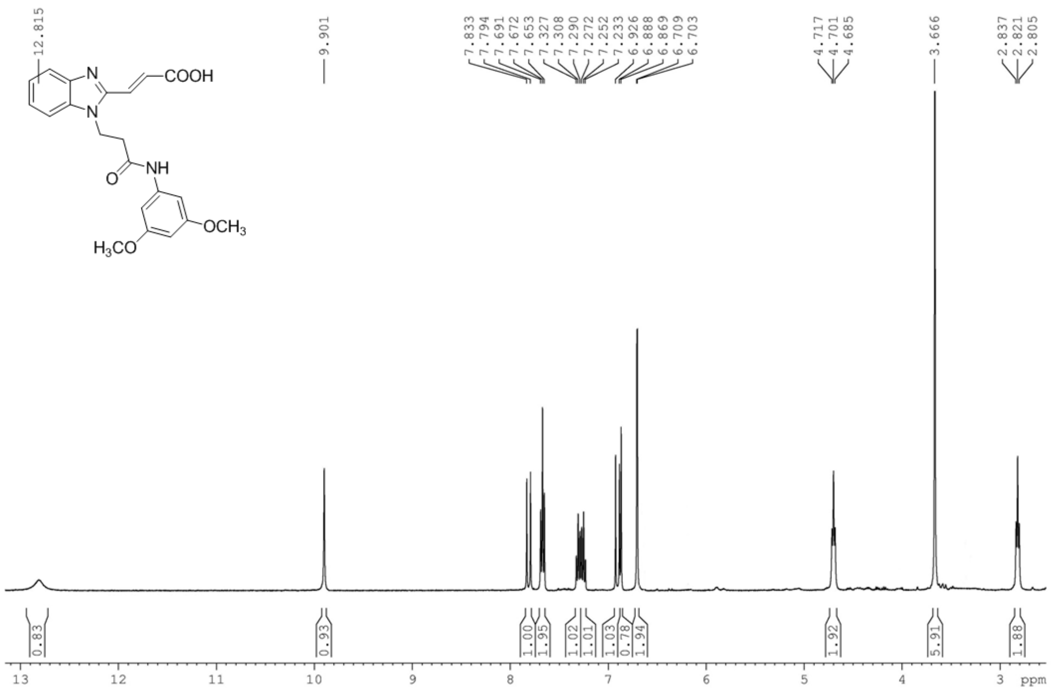

¹H-NMR spectra of 6i

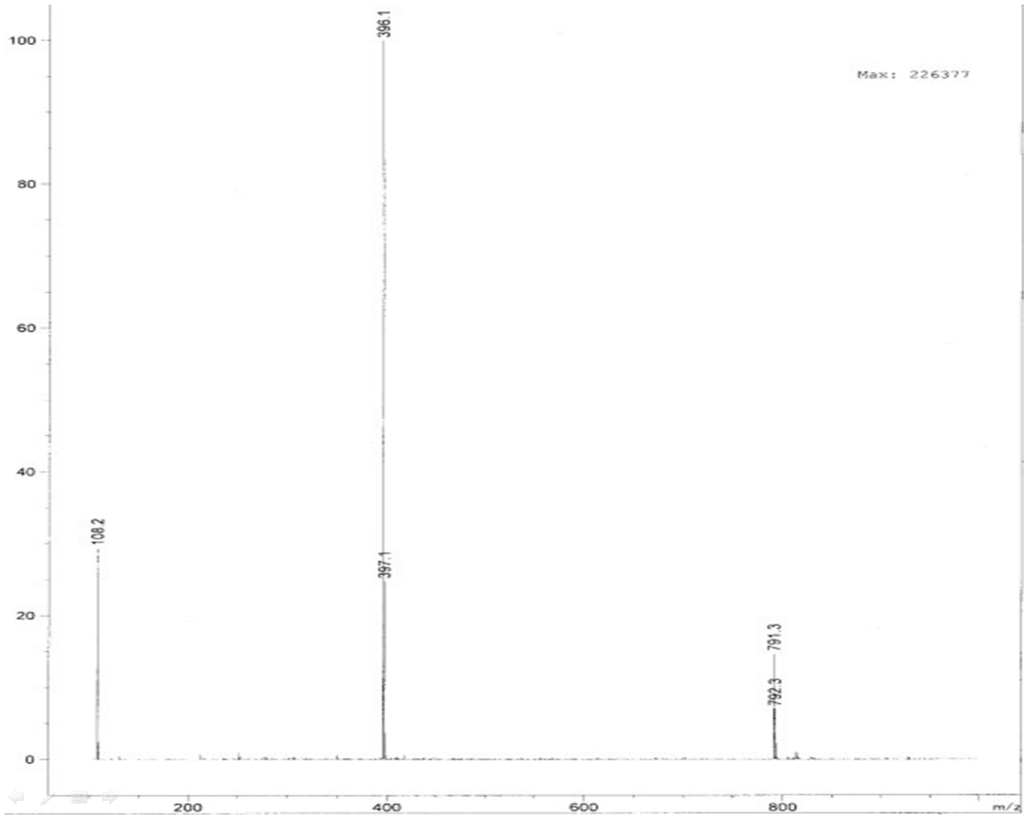

MS spectra of 6i

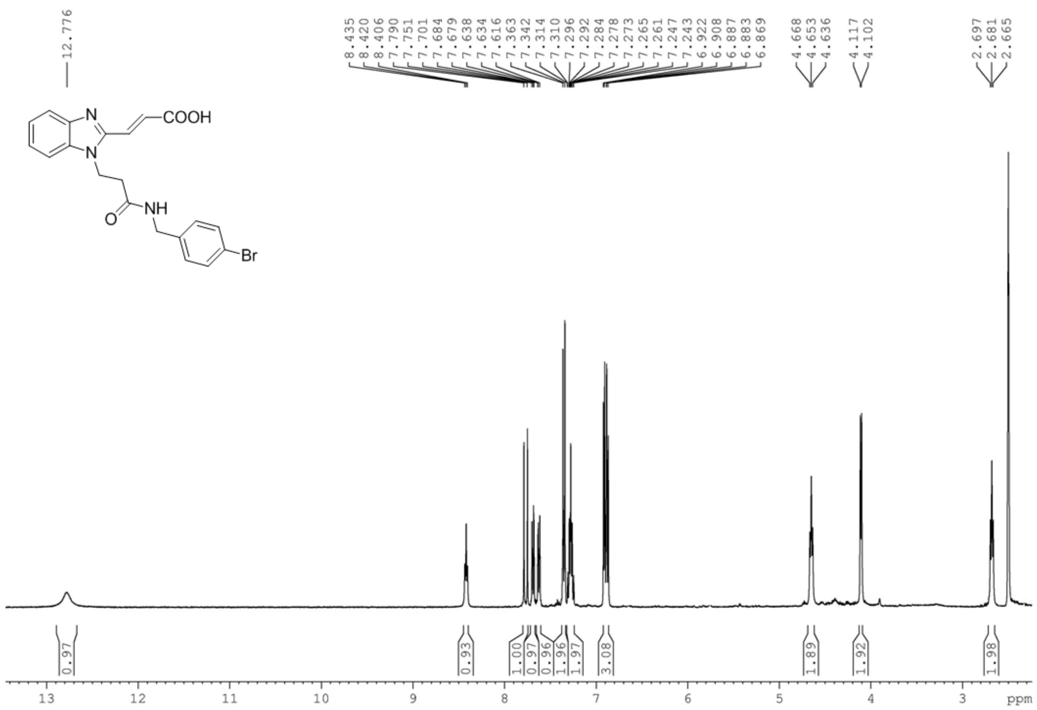

¹H-NMR spectra of 6j

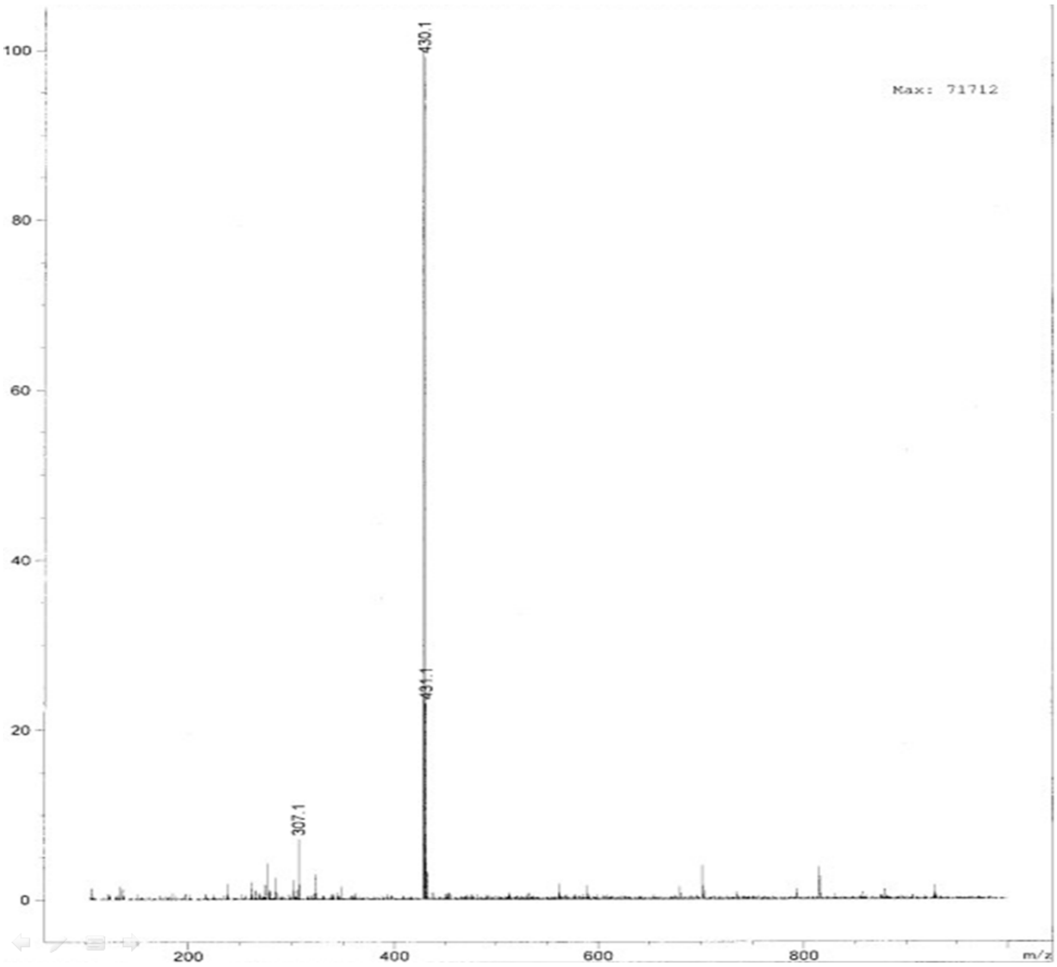

MS spectra of 6j

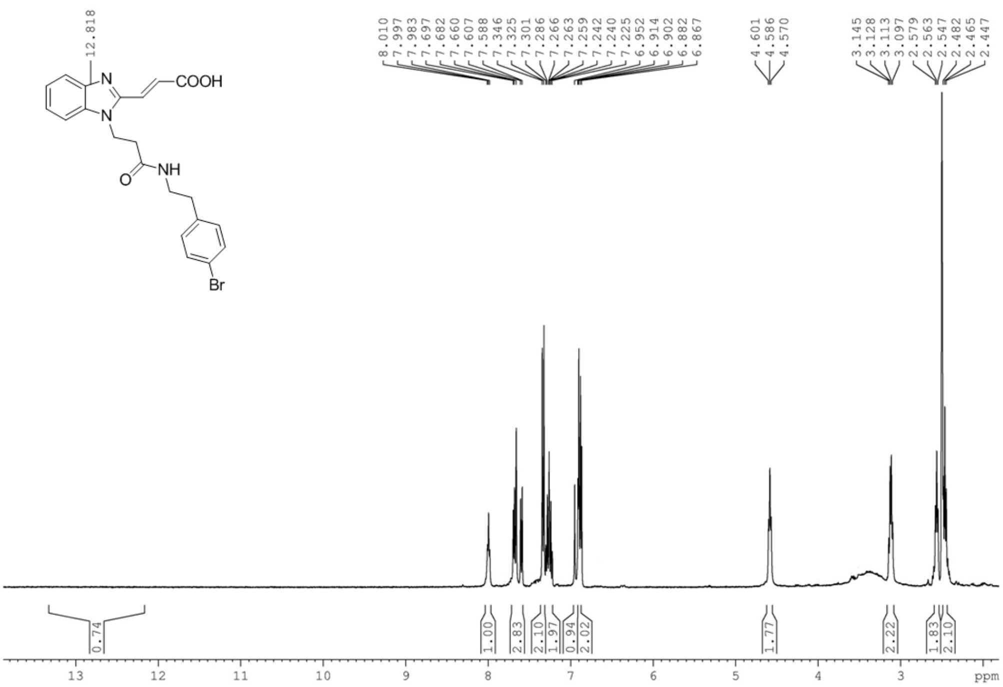

¹H-NMR spectra of 6k

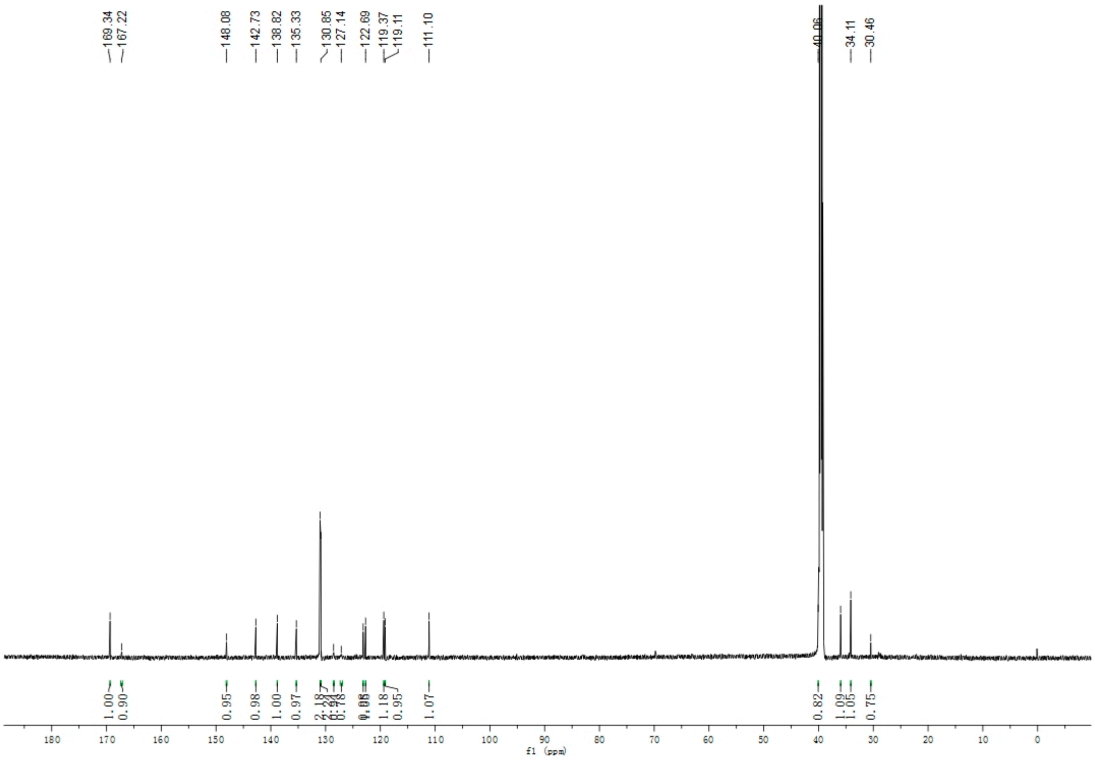

¹³C-NMR spectra of 6k

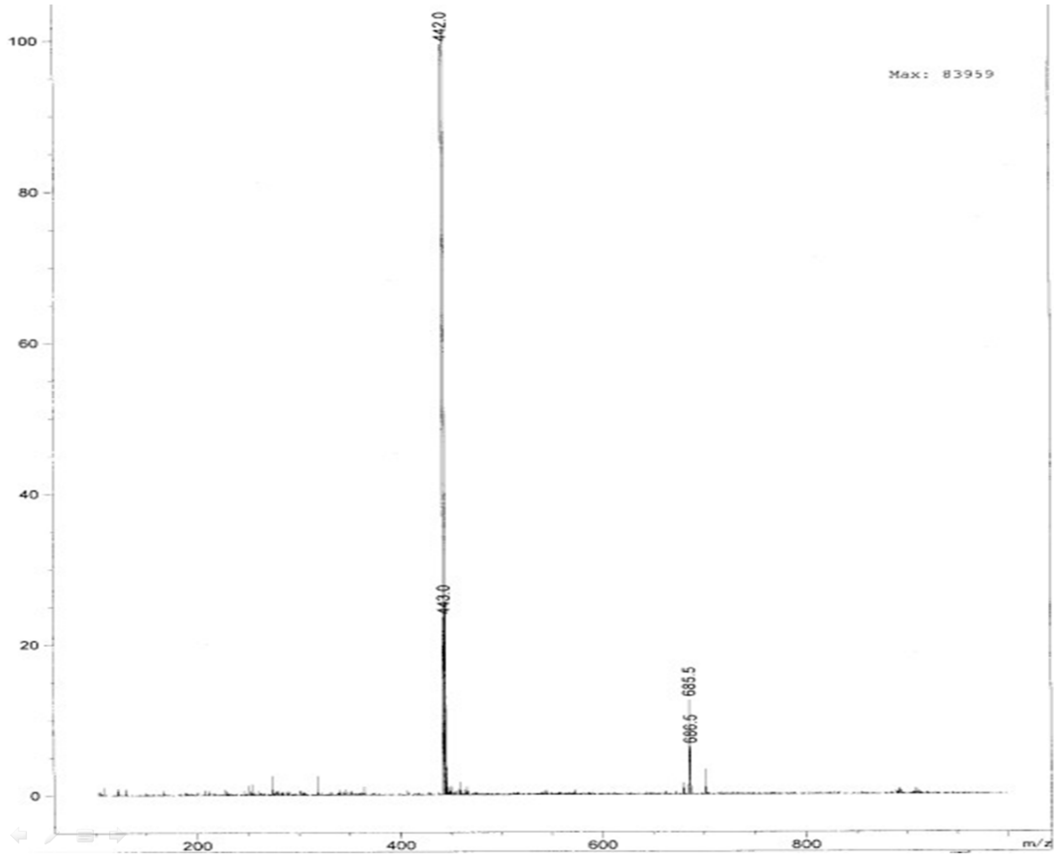

MS spectra of 6k

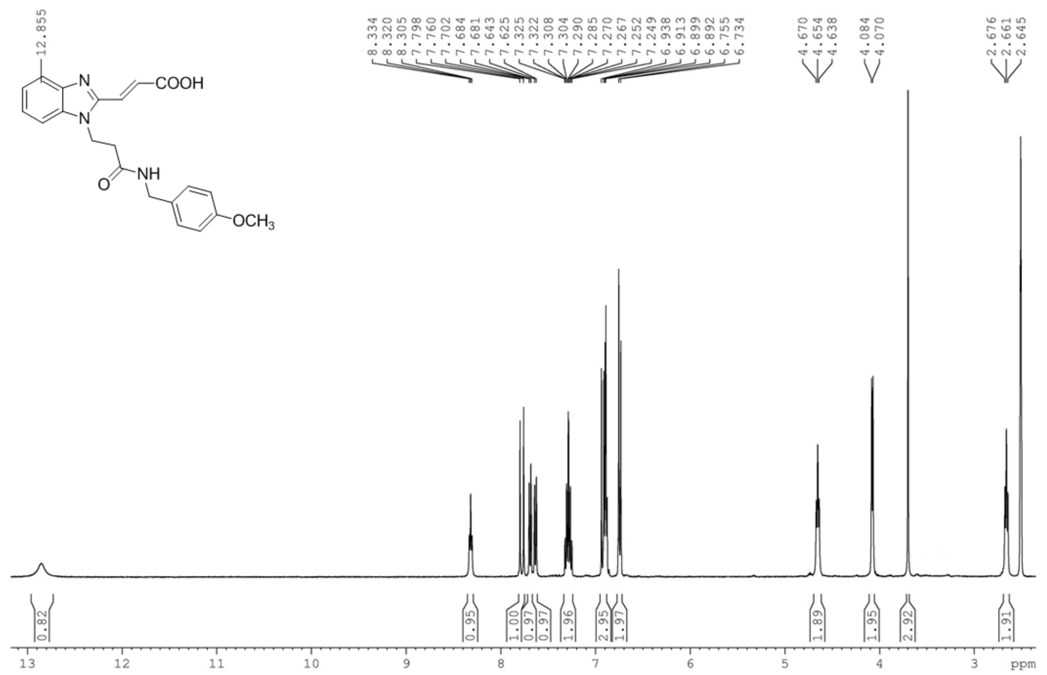

¹H-NMR spectra of 6l

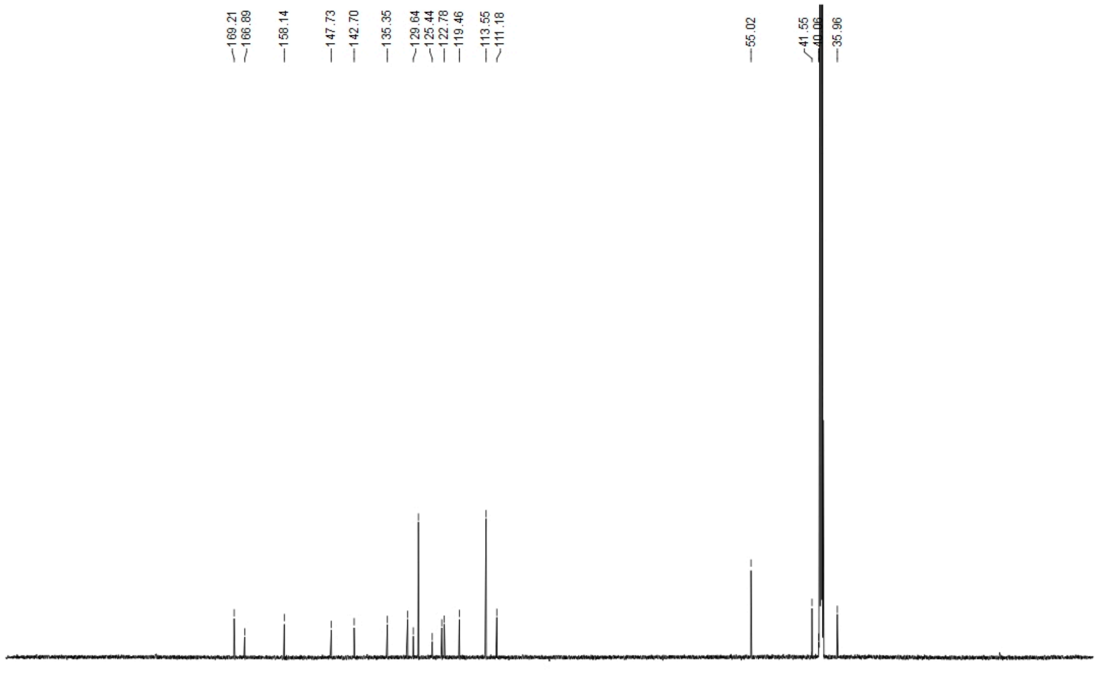

¹³C-NMR spectra of 6l

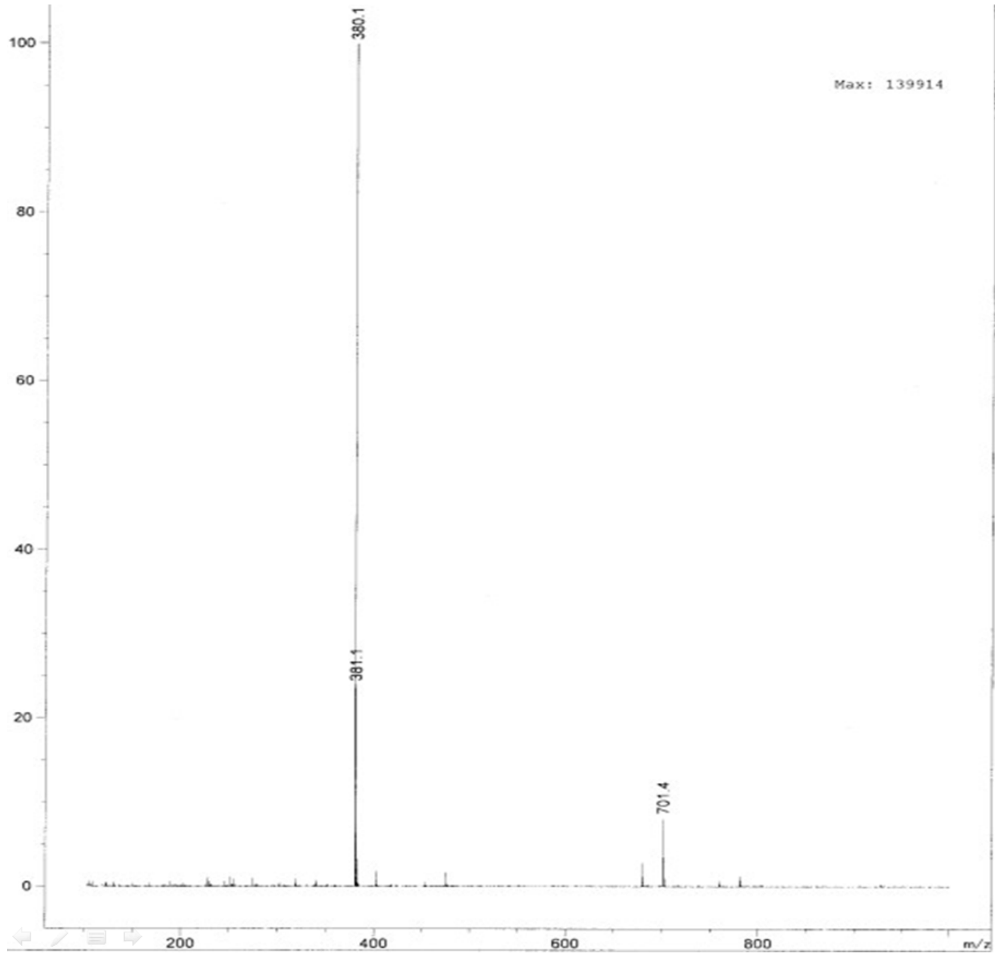

MS spectra of 6l

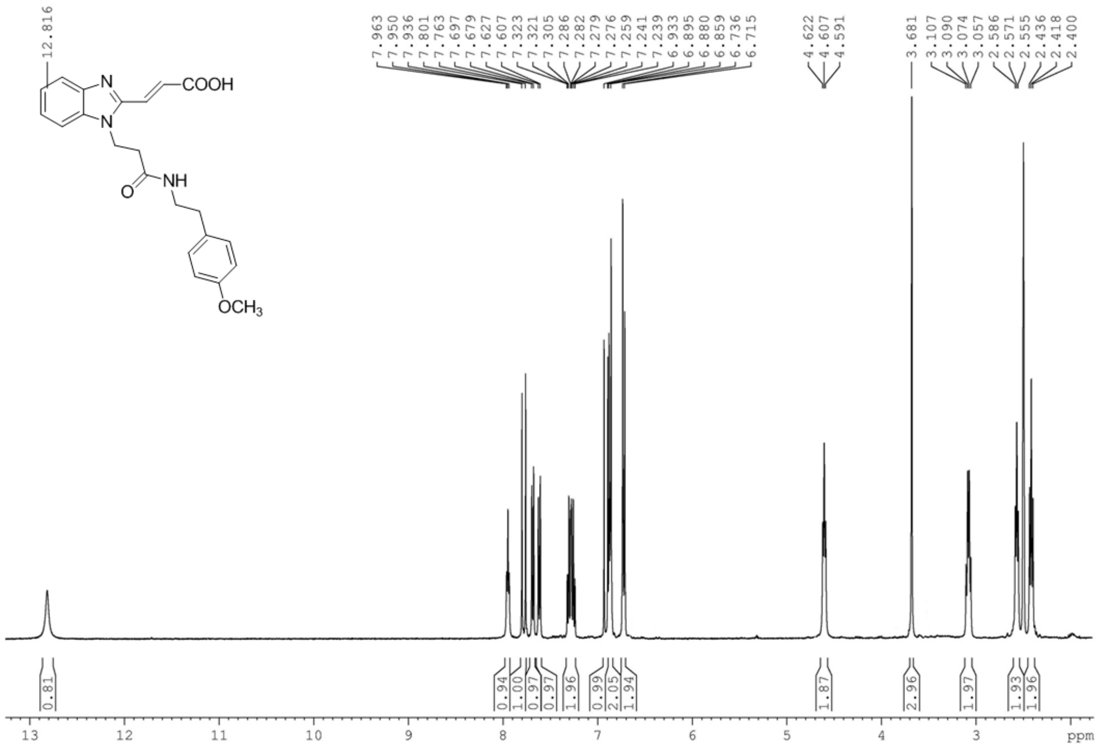

^{1}H-NMR spectra of 6m

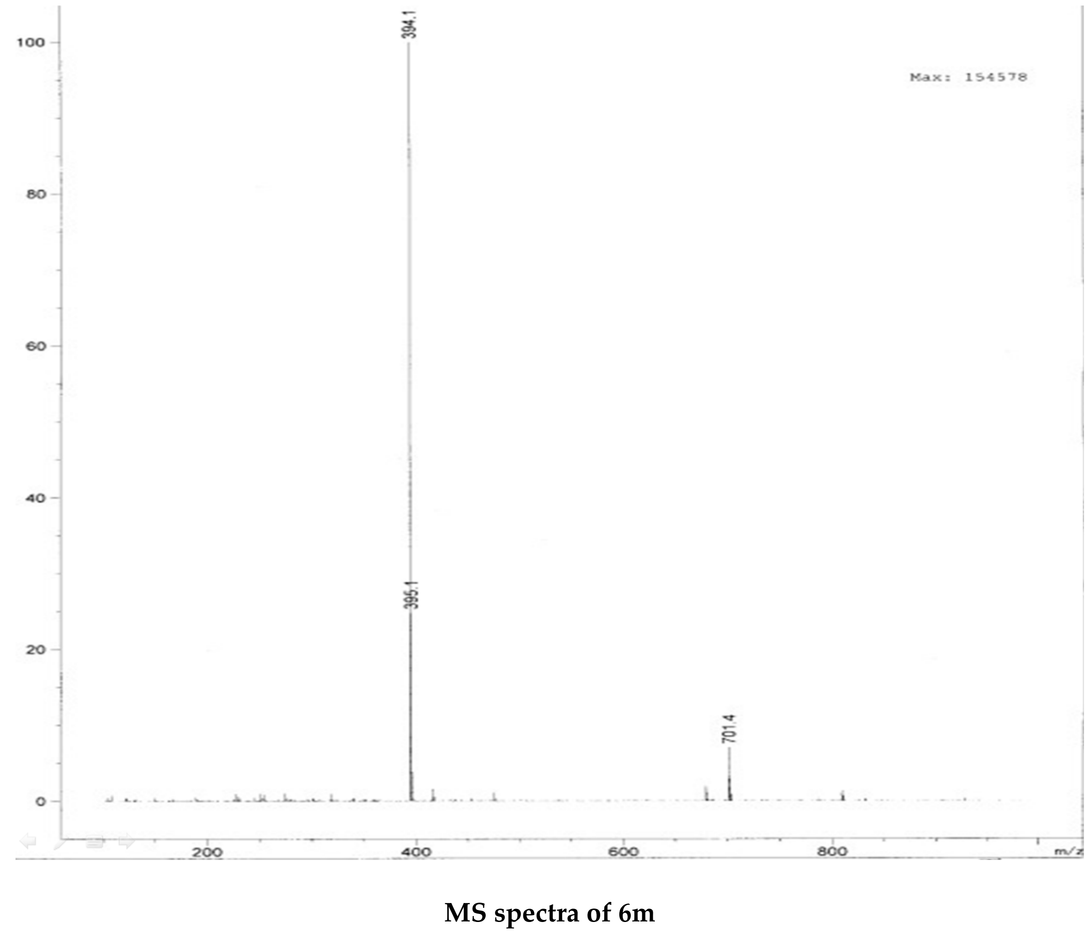

MS spectra of 6m

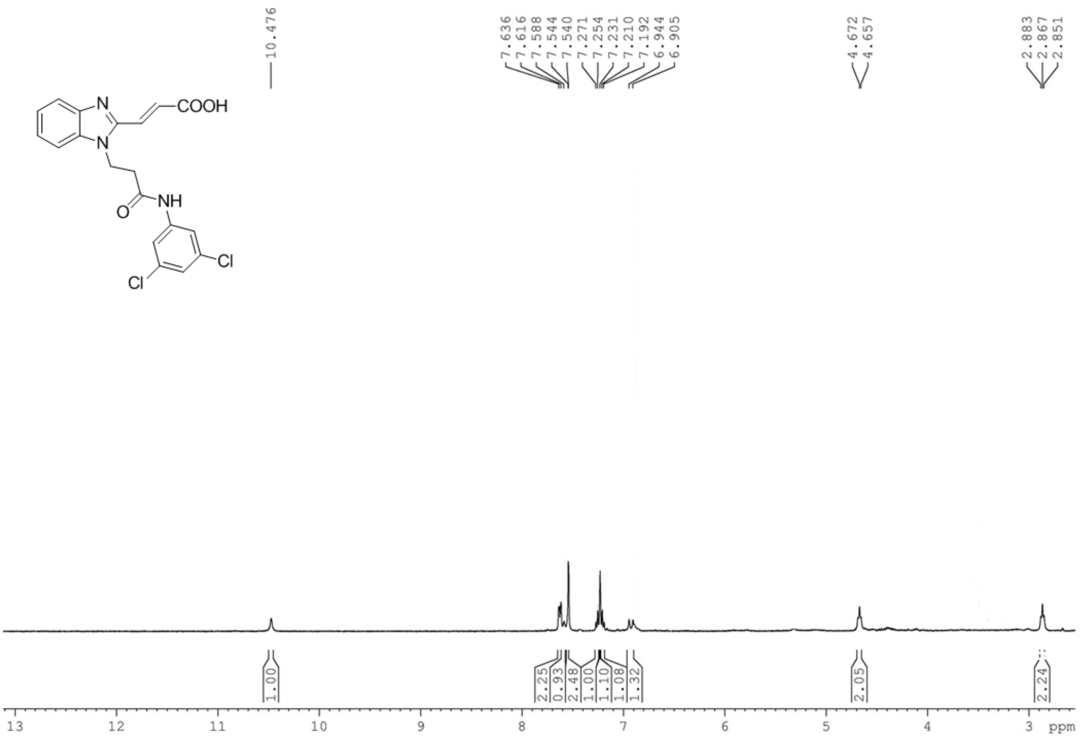

^{1}H-NMR spectra of 6n

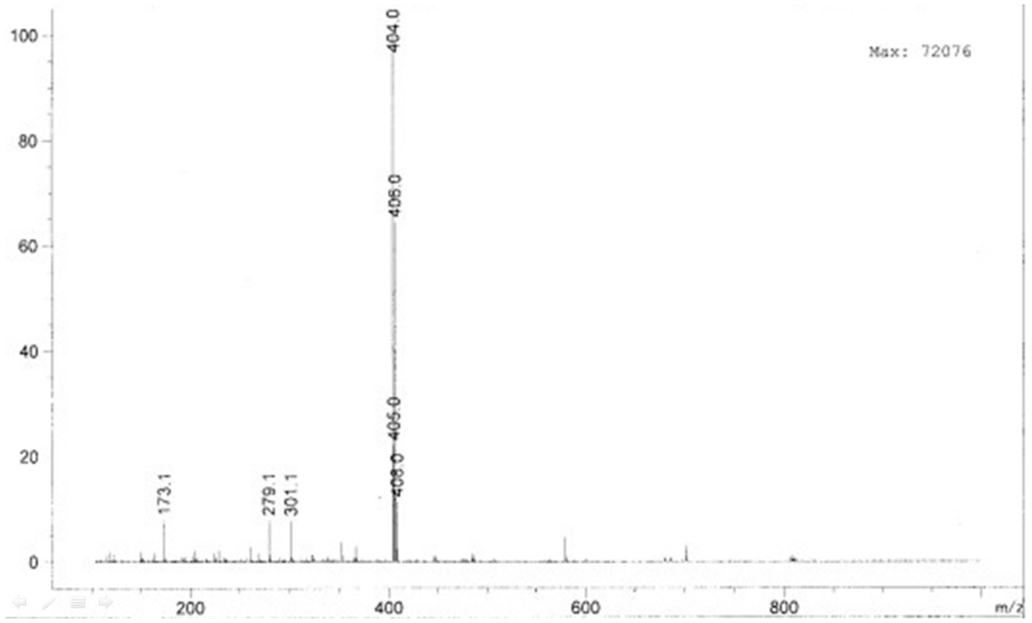

MS spectra of 6n

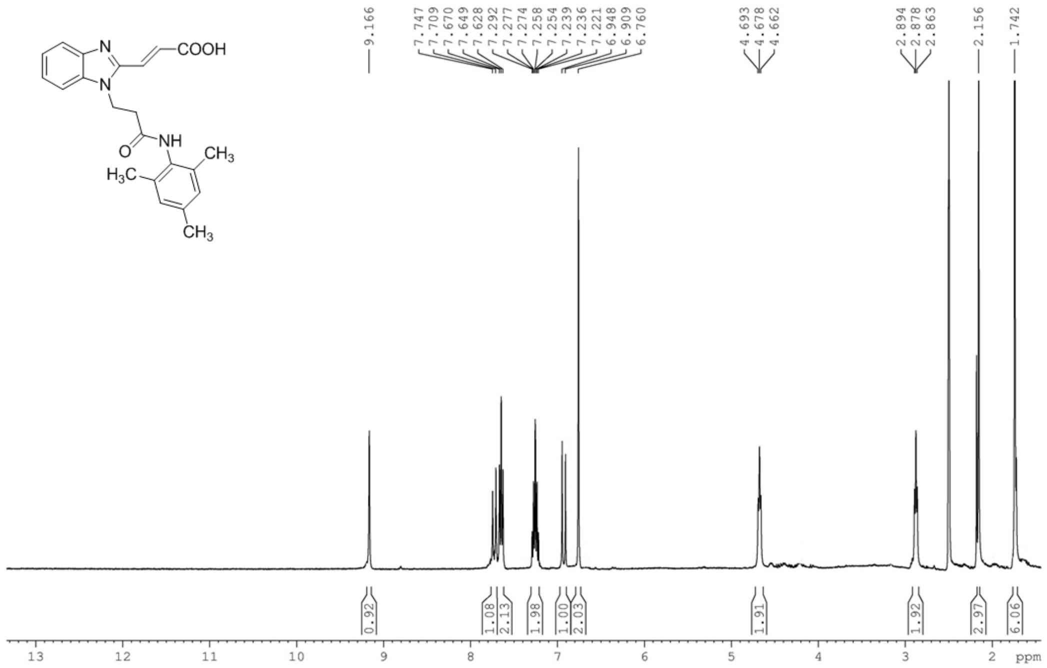

¹H-NMR spectra of 6o

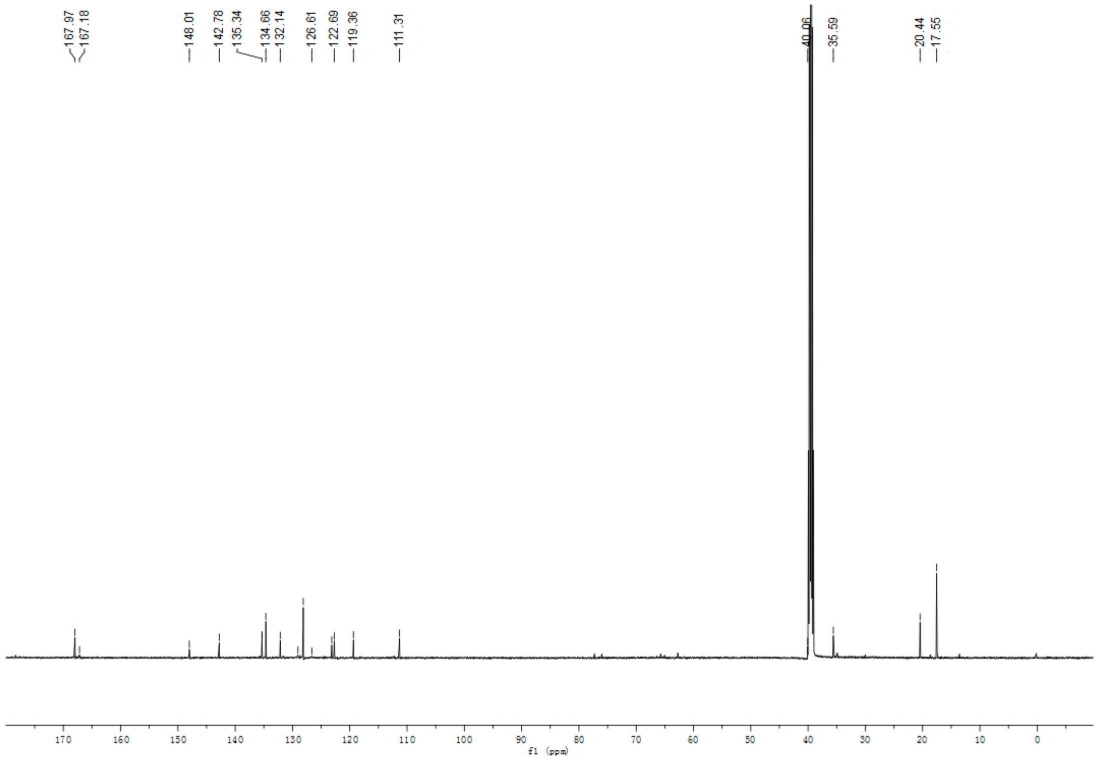

¹³C-NMR spectra of 6o

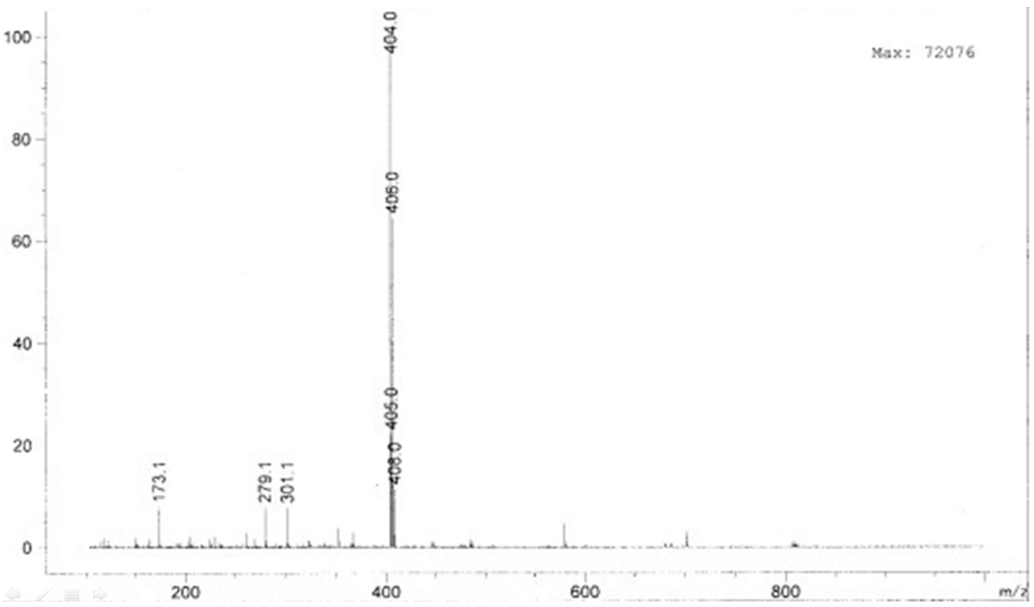

MS spectra of 6o

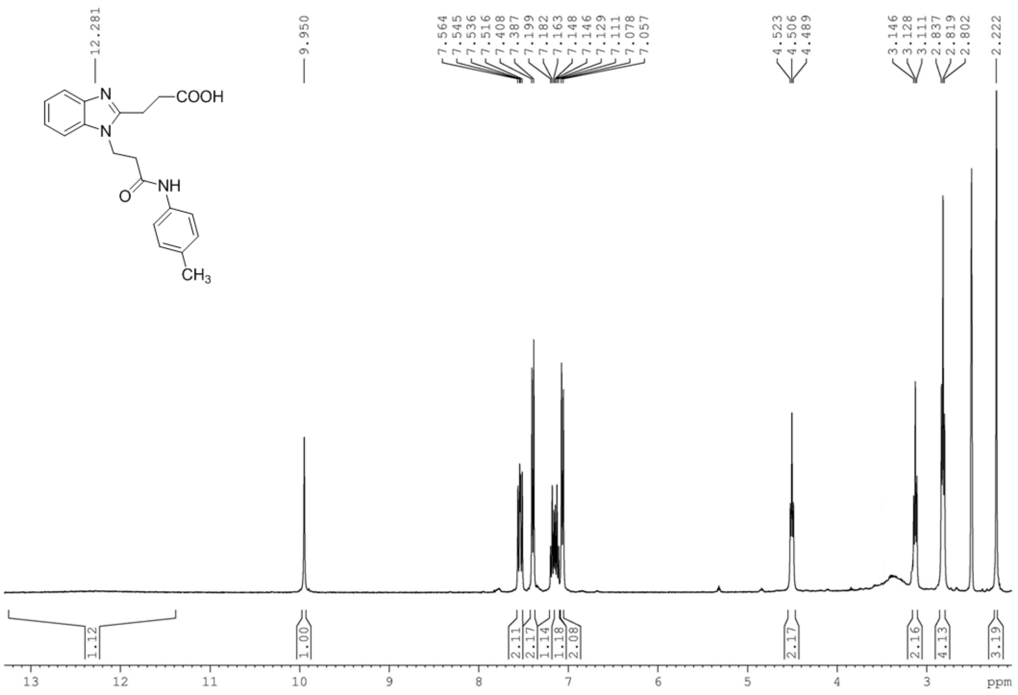

¹H-NMR spectra of 7a

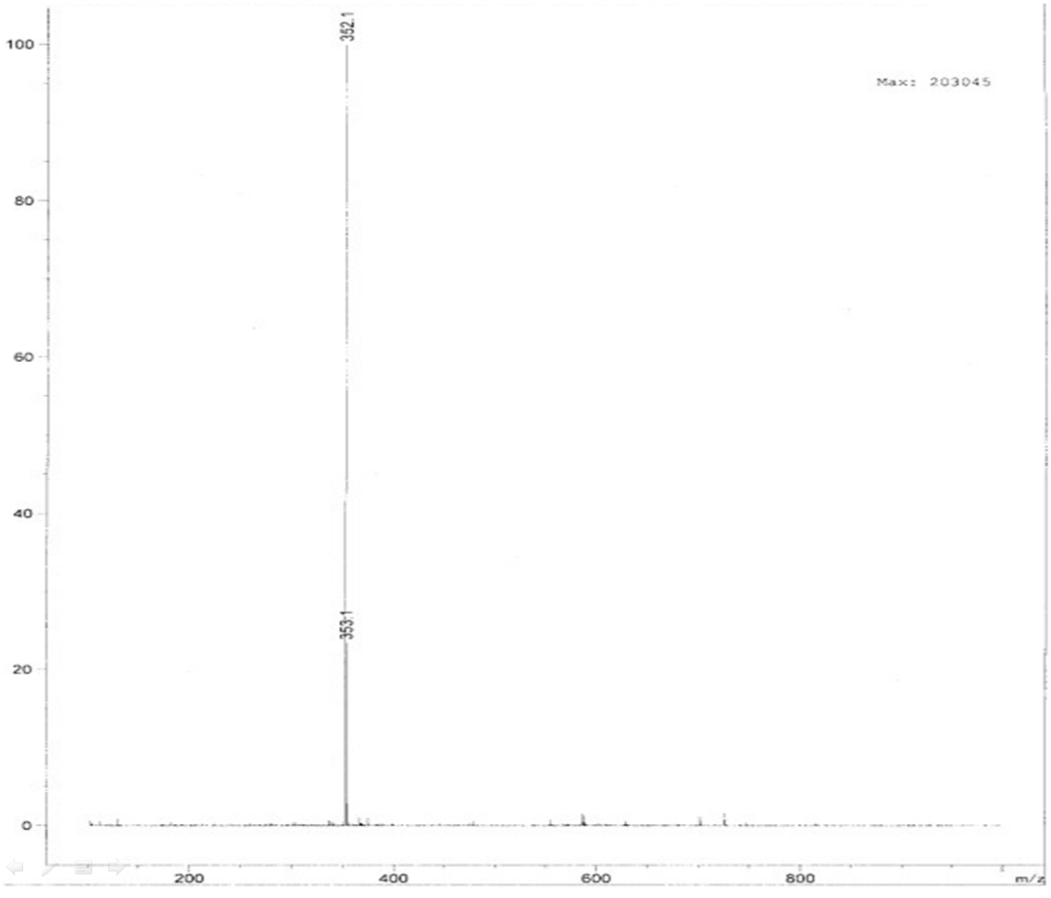

MS spectra of 7a

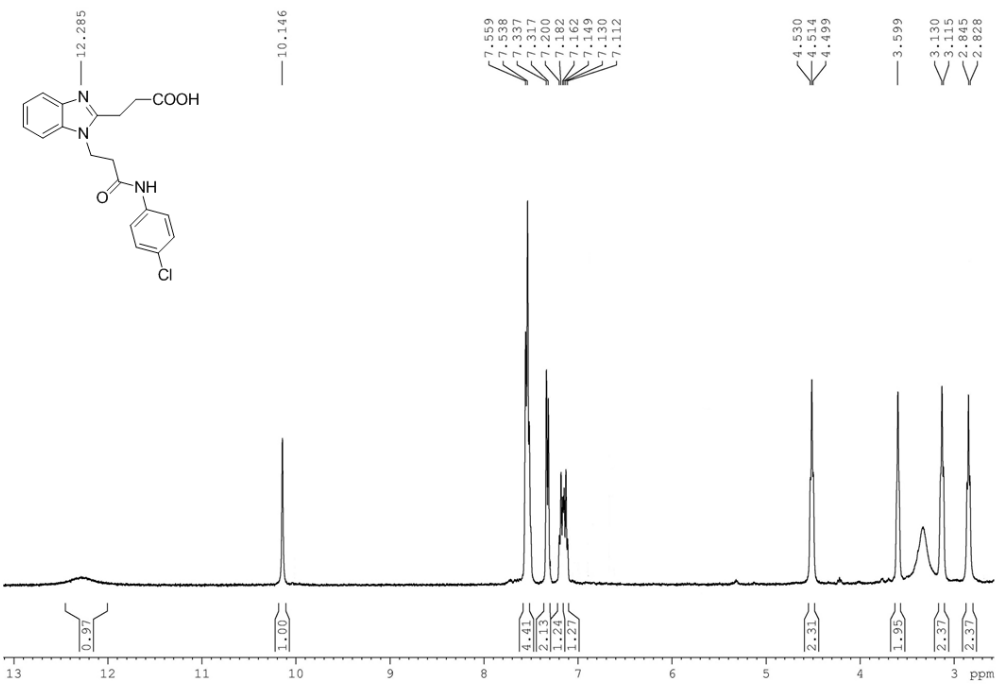

¹H-NMR spectra of 7b

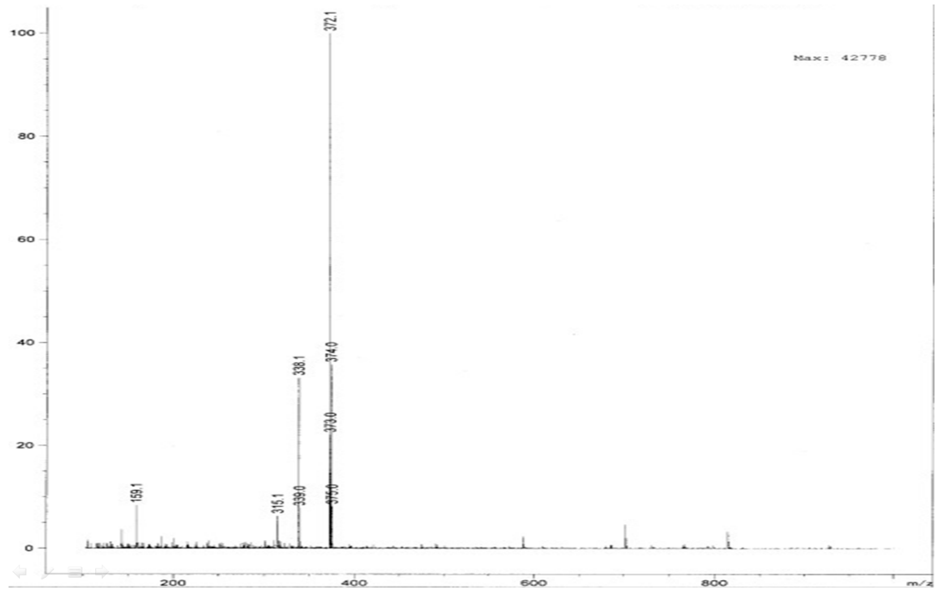

MS spectra of 7b

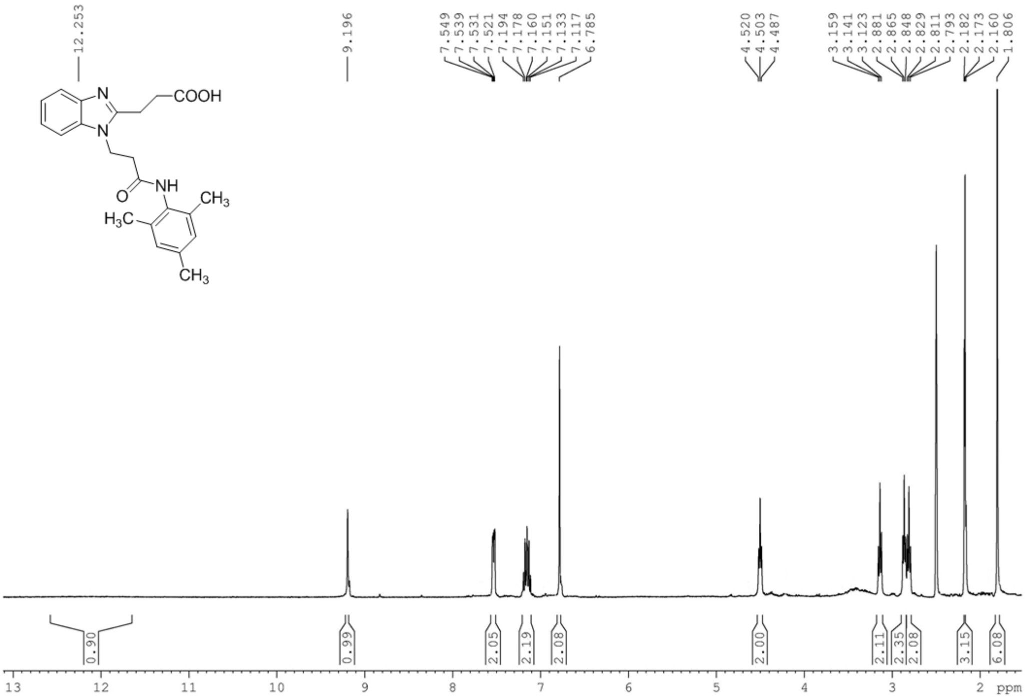

¹H-NMR spectra of 7c

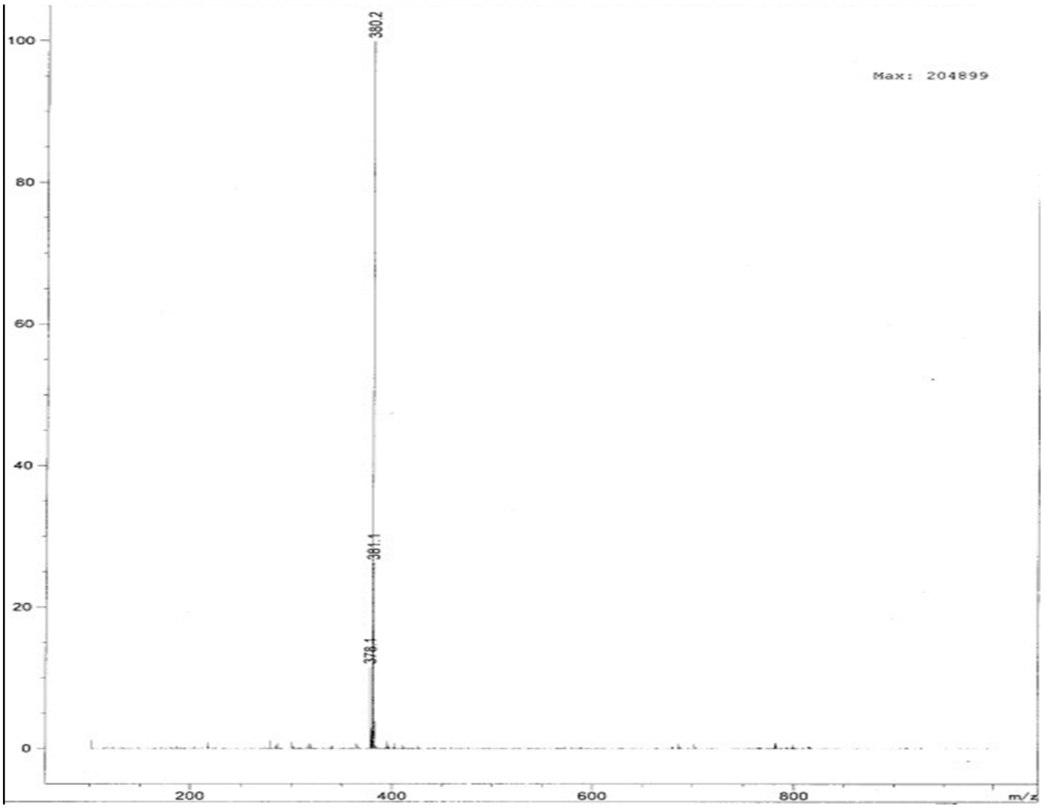

MS spectra of 7c

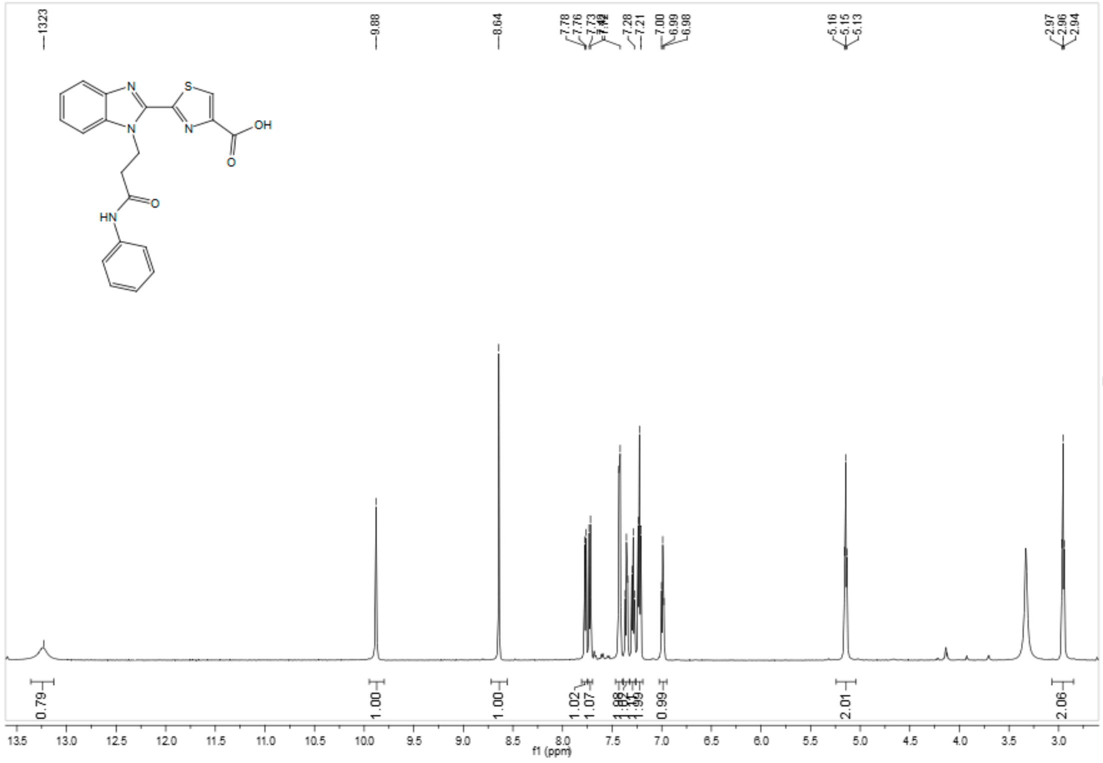

^{1}H-NMR spectra of 13a

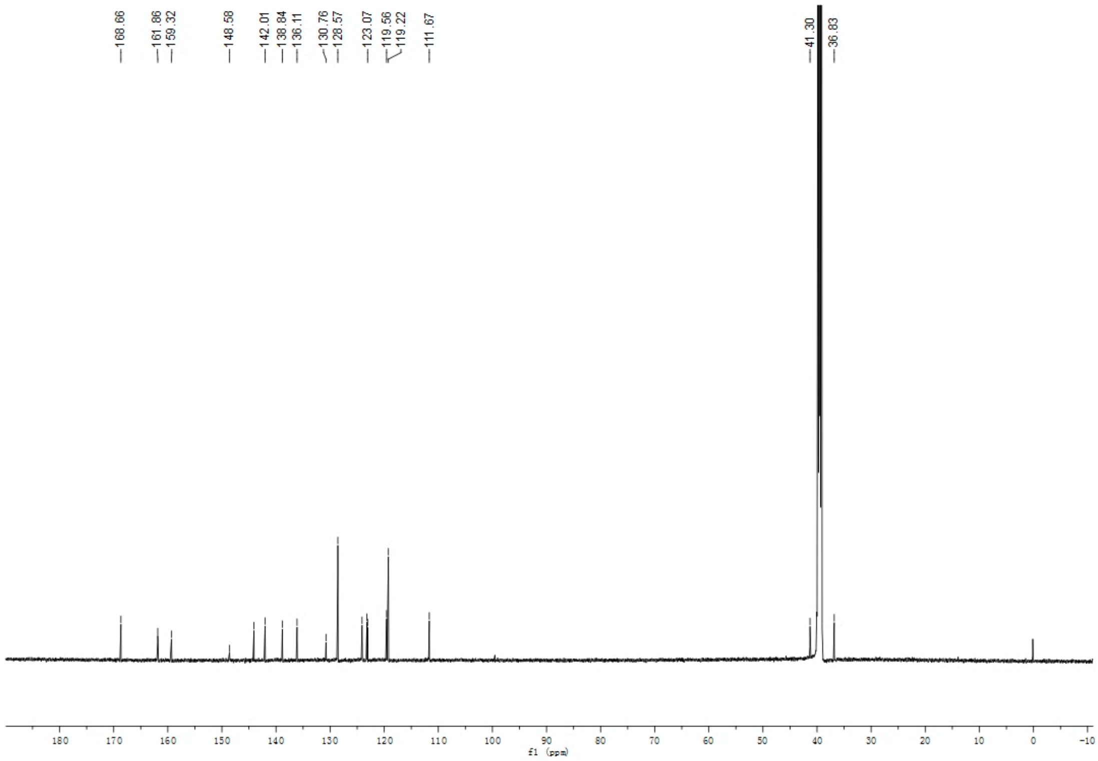

^{13}C-NMR spectra of 13a

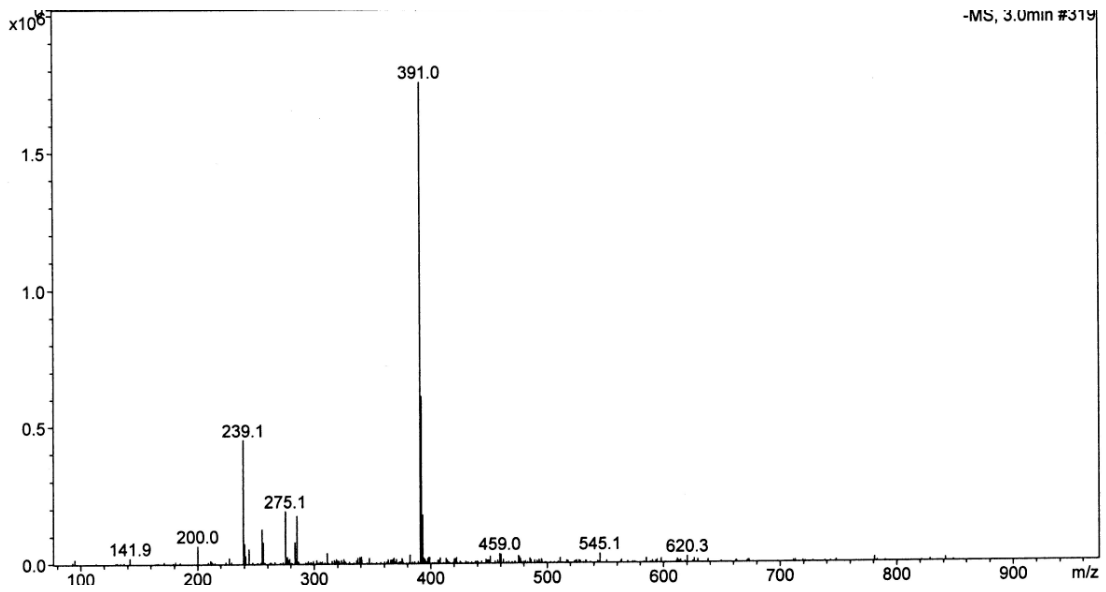

MS spectra of 13a

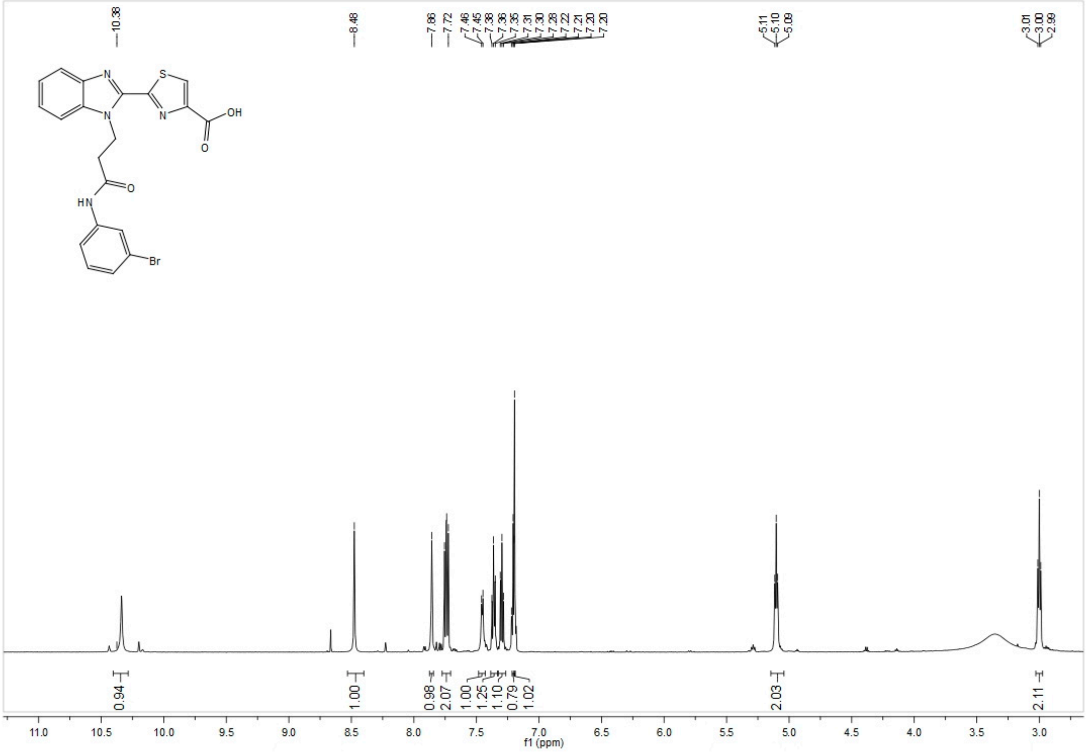

^{1}H-NMR spectra of 13b

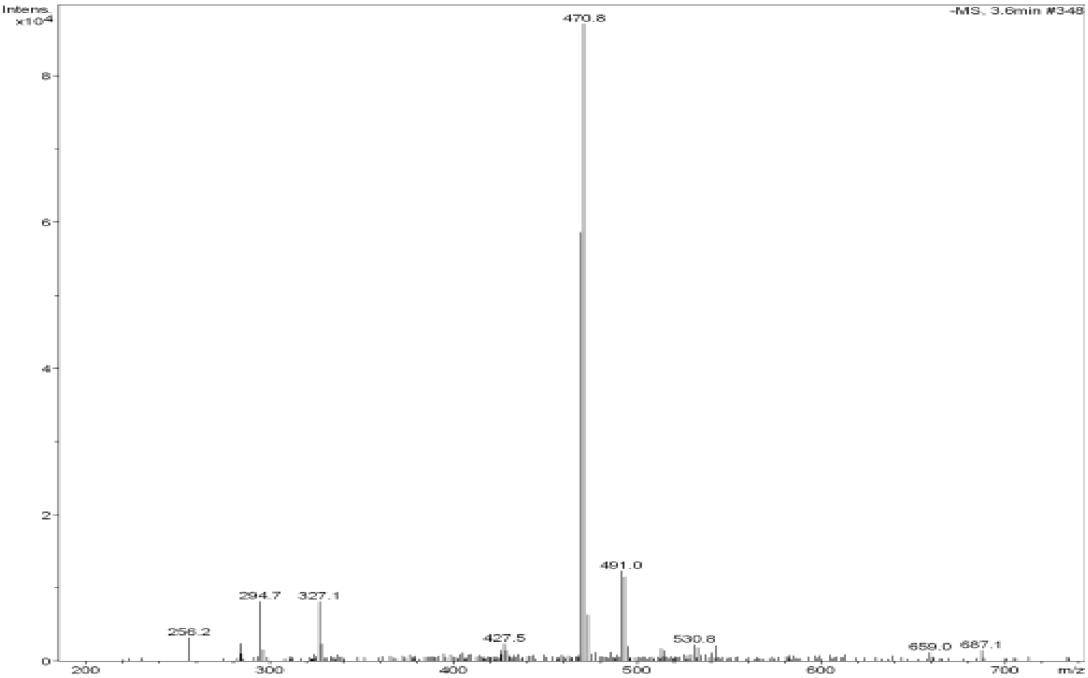

MS spectra of 13b

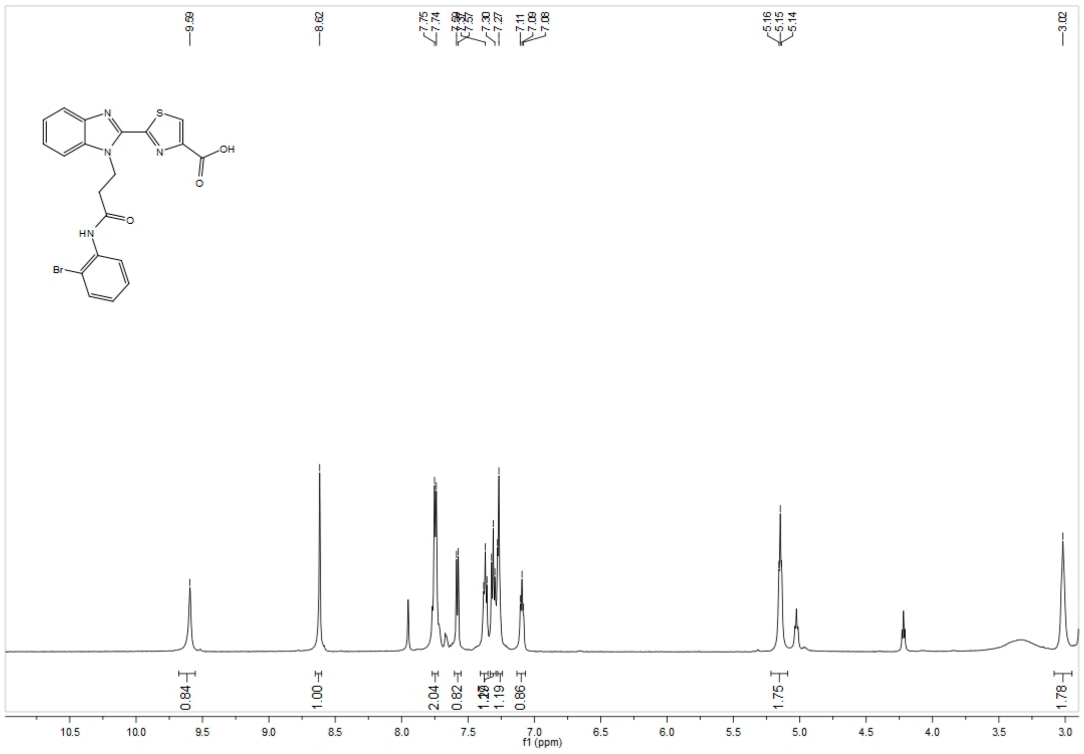

¹H-NMR spectra of 13c

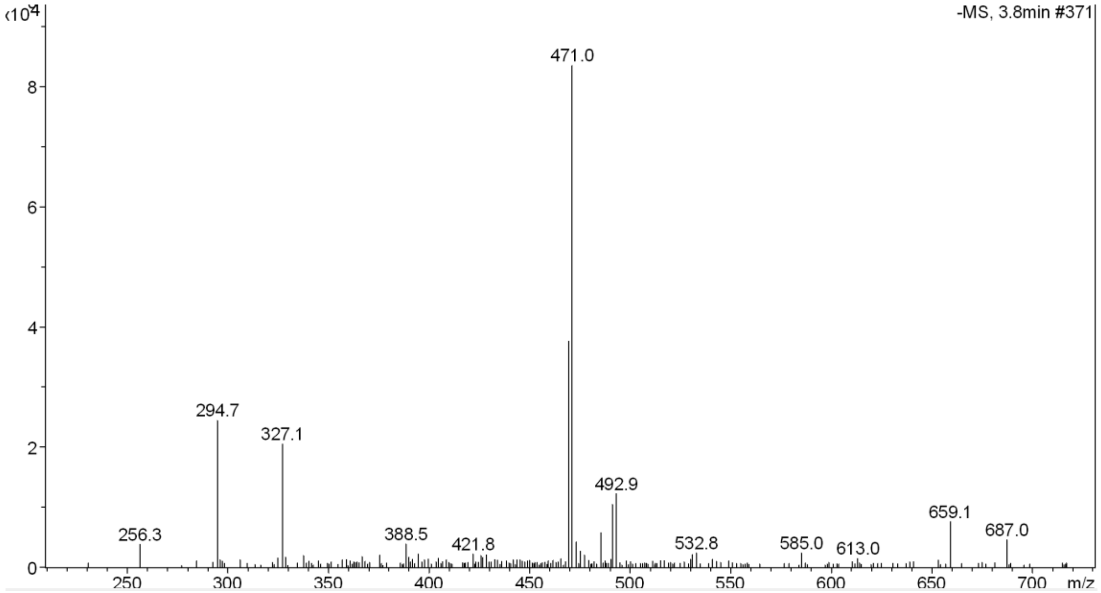

MS spectra of 13c

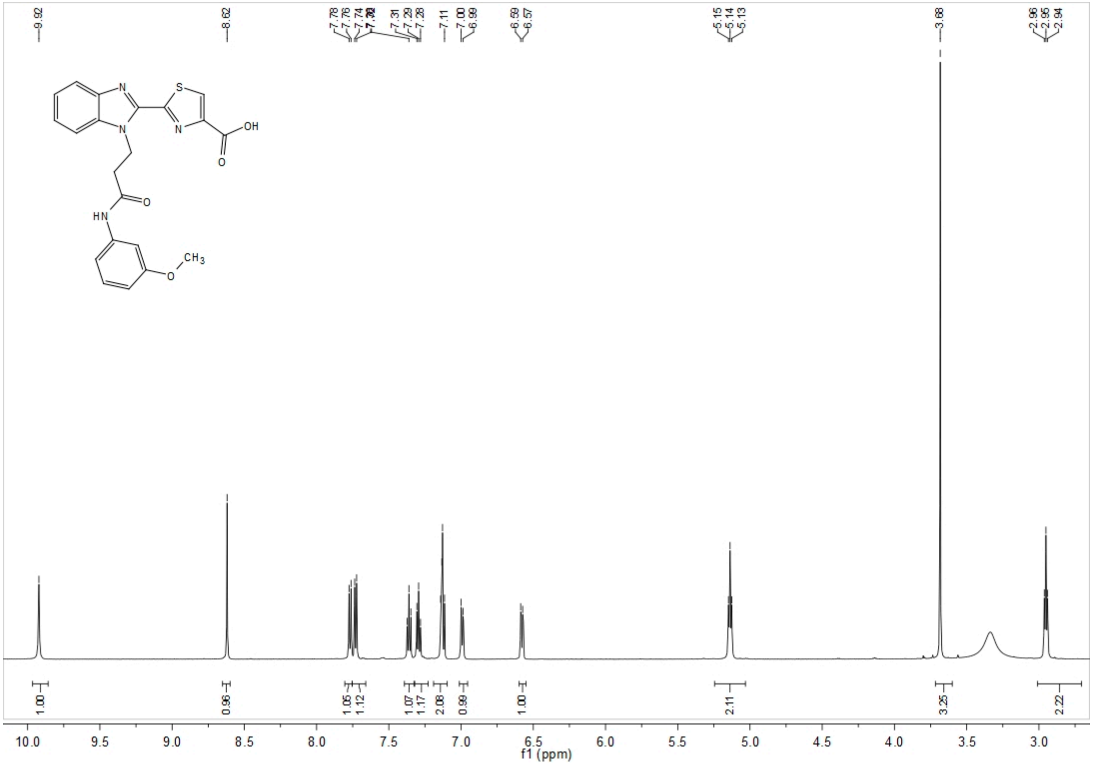

^{1}H-NMR spectra of 13d

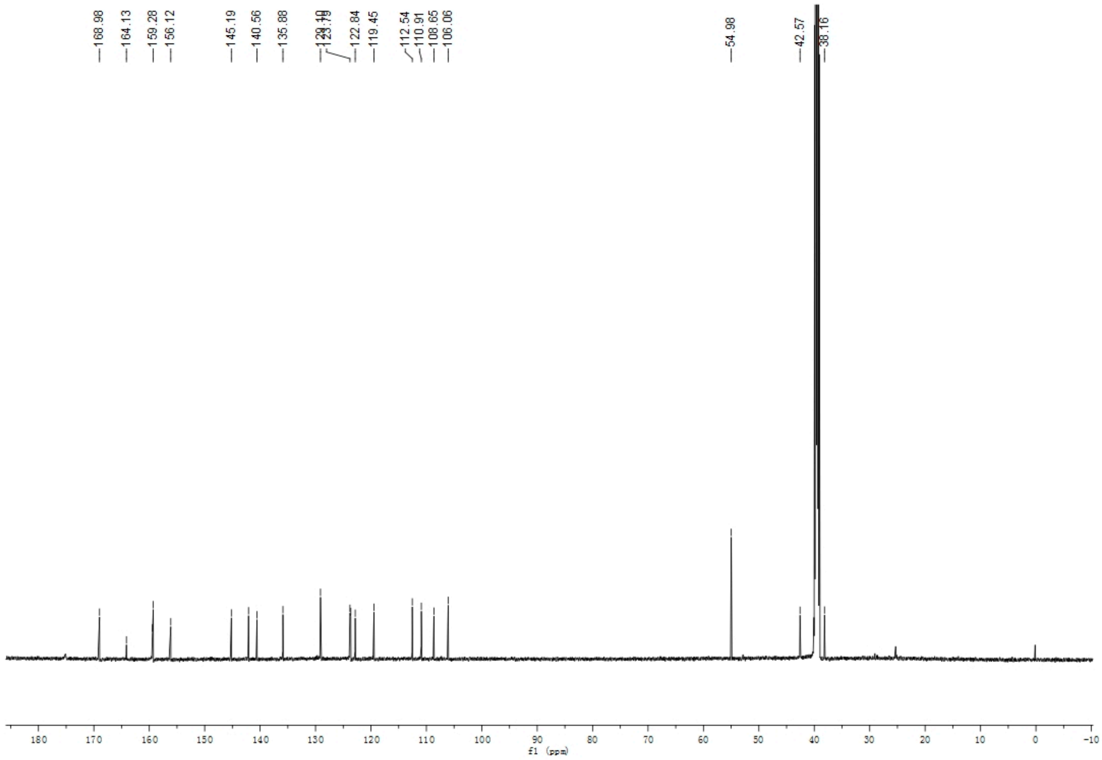

^{13}C-NMR spectra of 13d

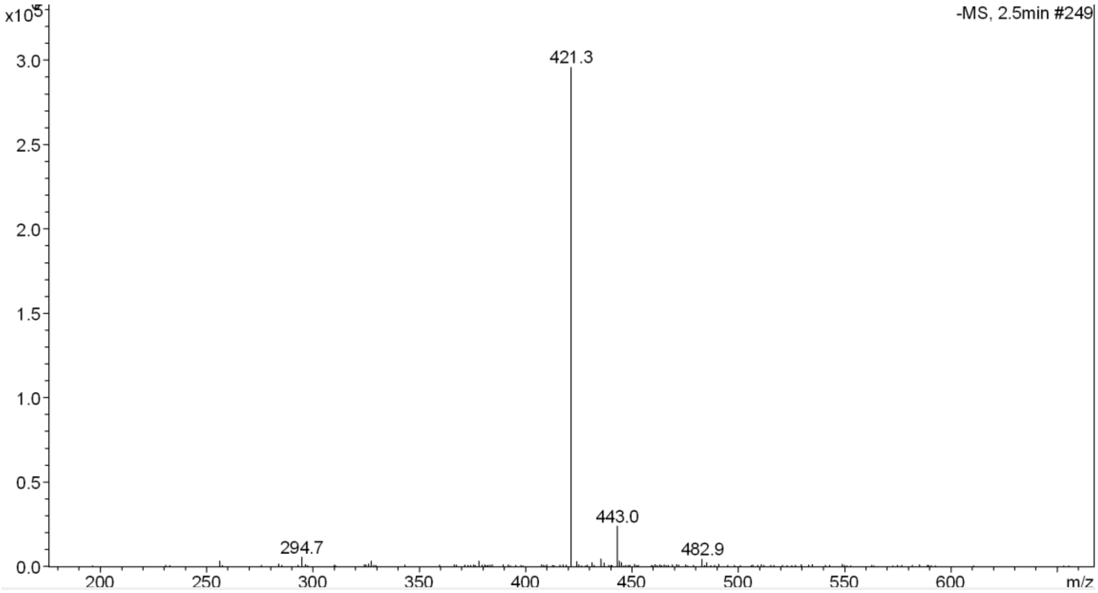

MS spectra of 13d

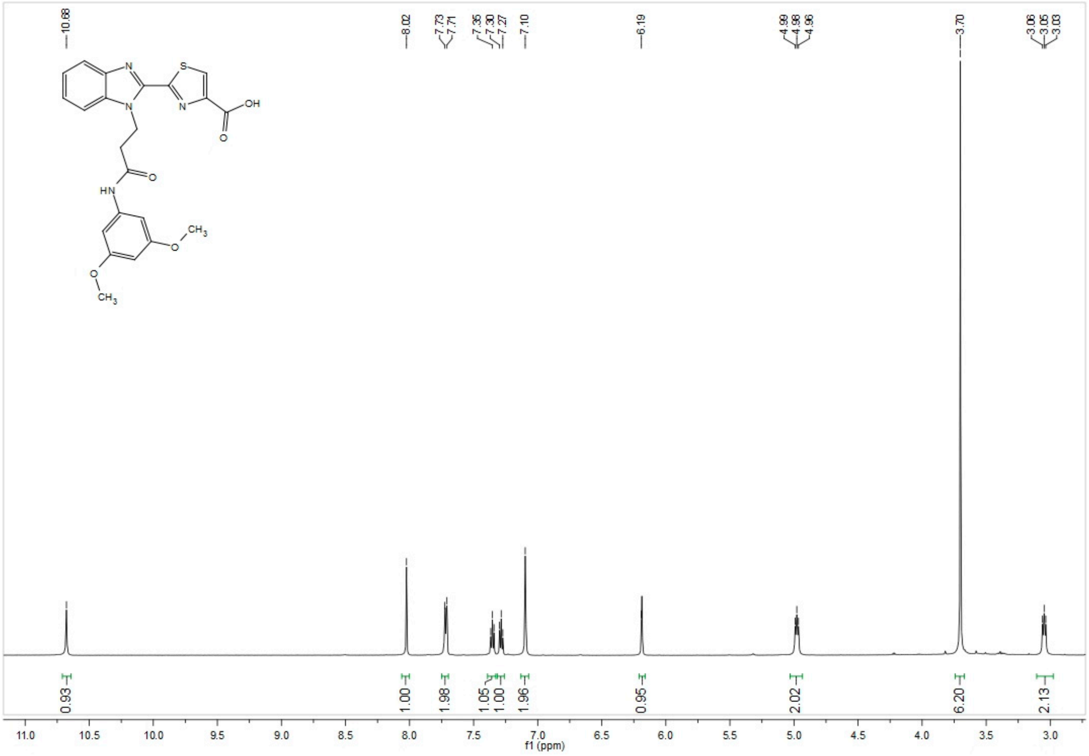

^{1}H-NMR spectra of 13e

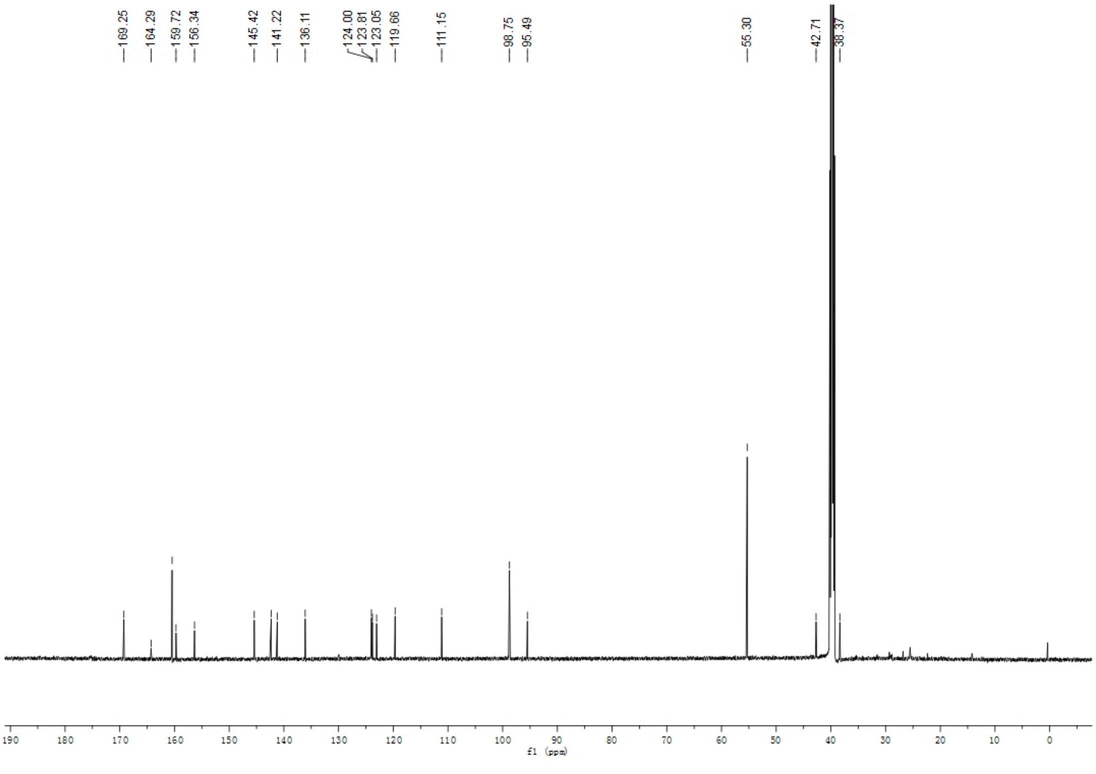

^{13}C-NMR spectra of 13e

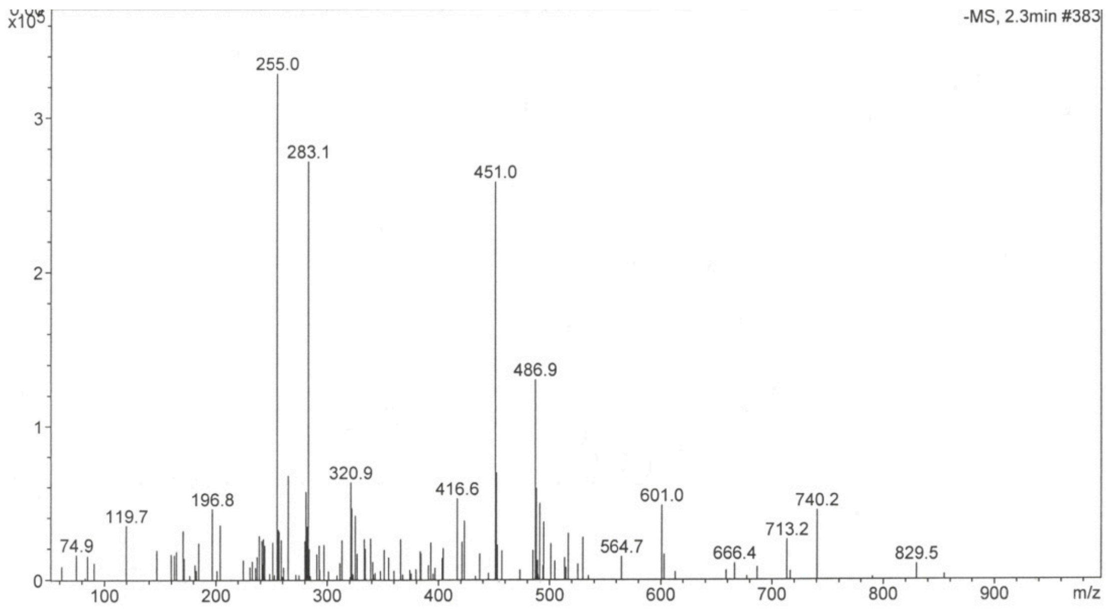

MS spectra of 13e

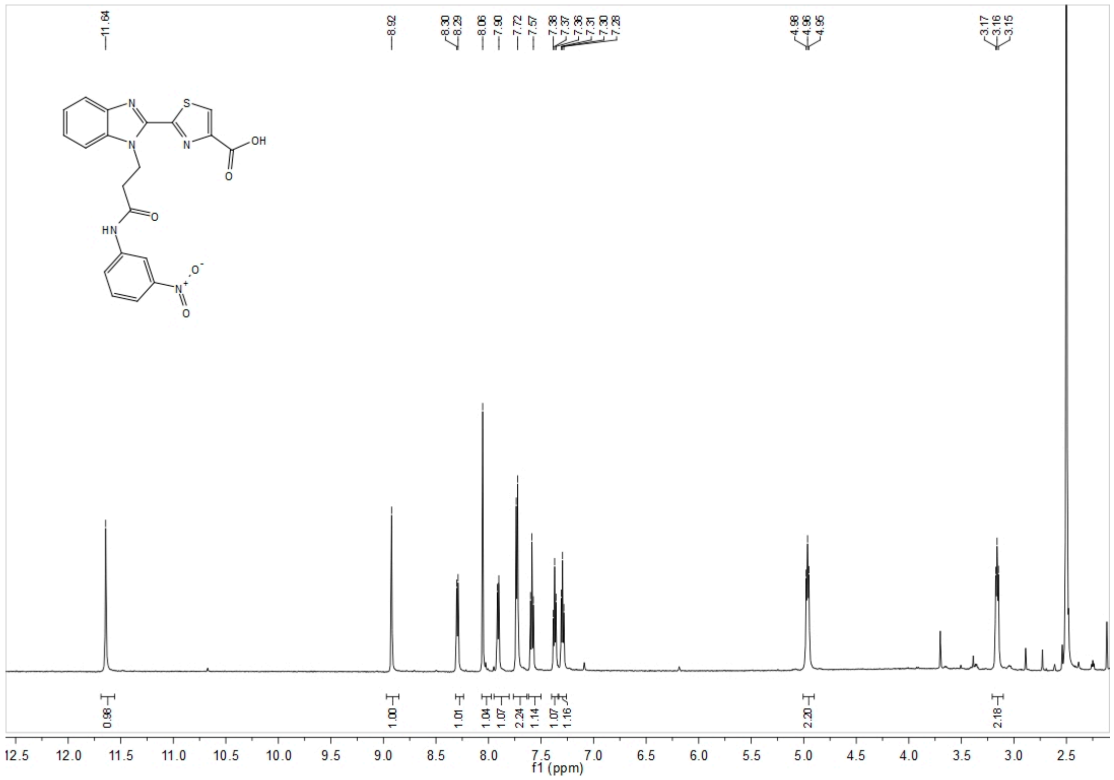

^{1}H-NMR spectra of 13f

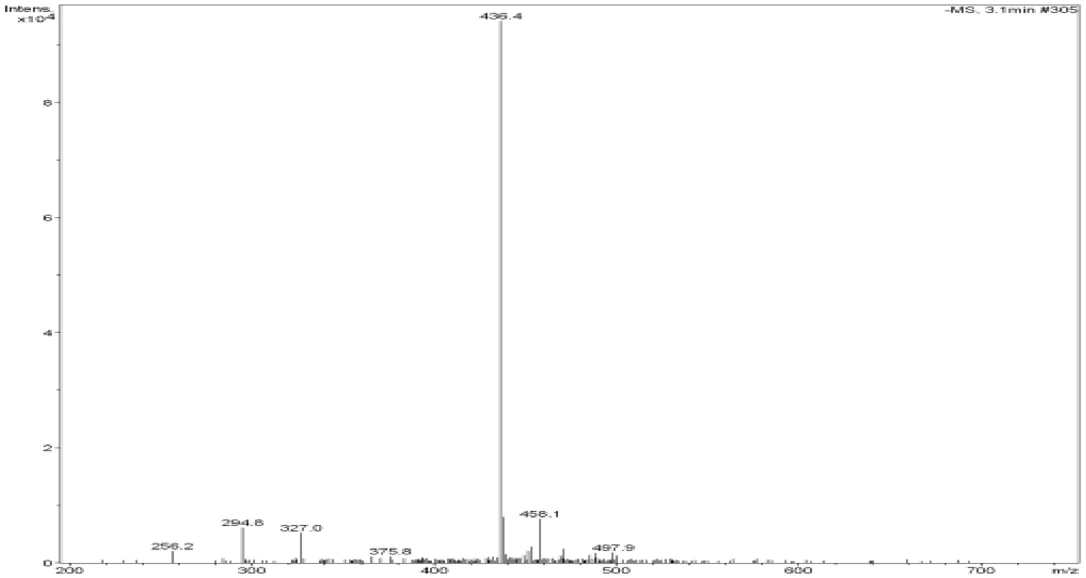

MS spectra of 13f

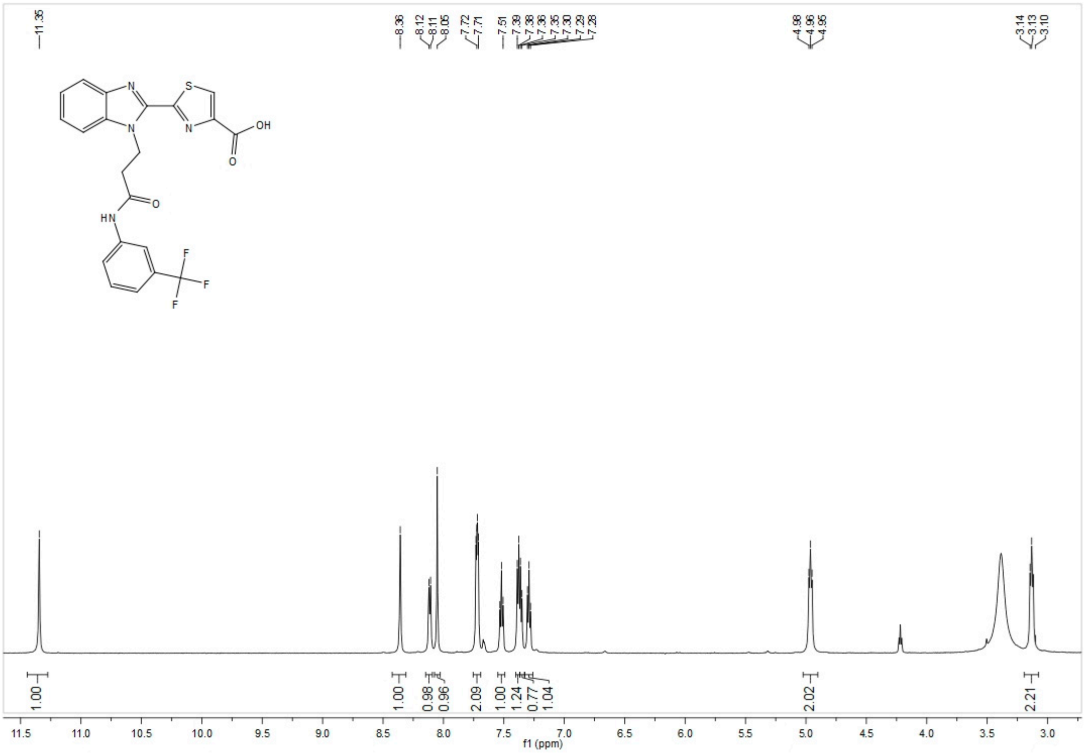

^{1}H-NMR spectra of 13g

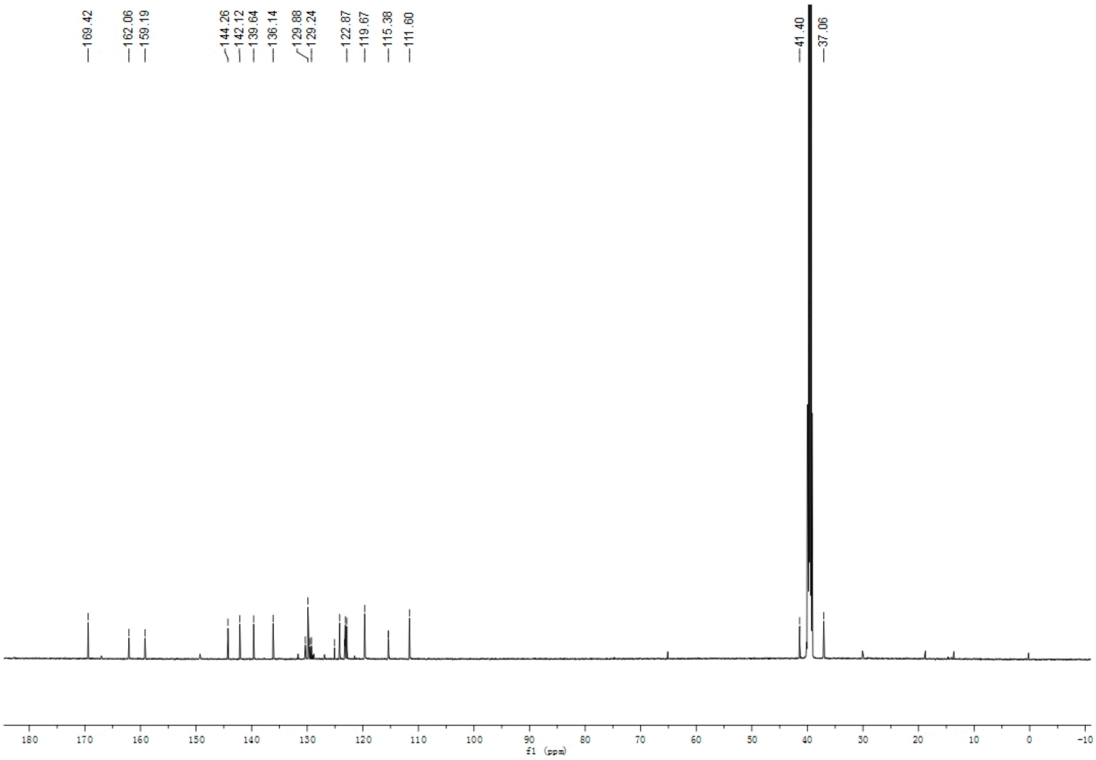

^{13}C-NMR spectra of 13g

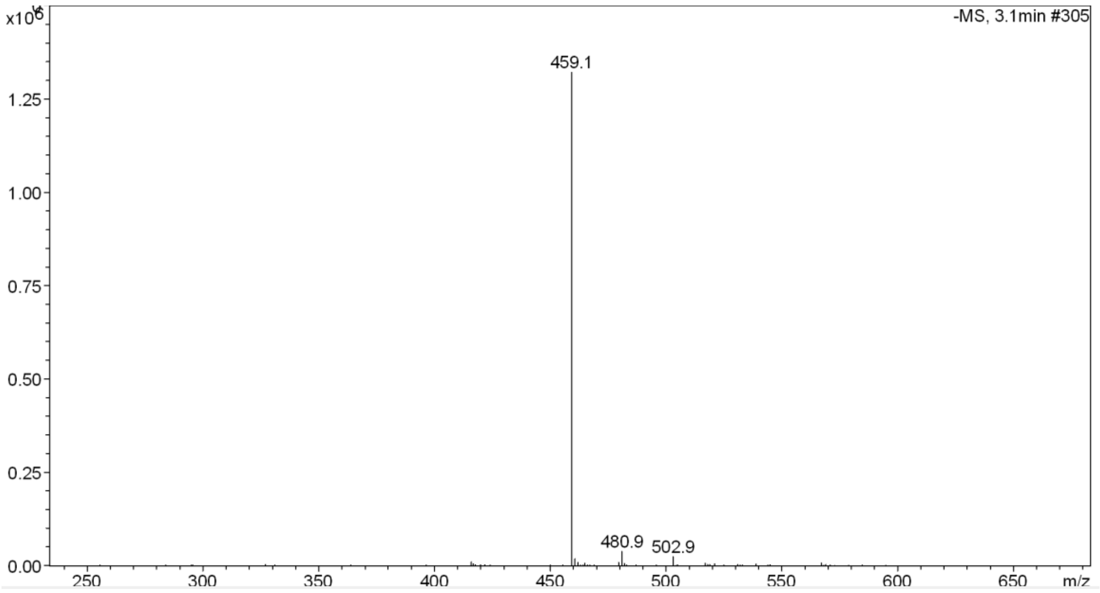

MS spectra of 13g

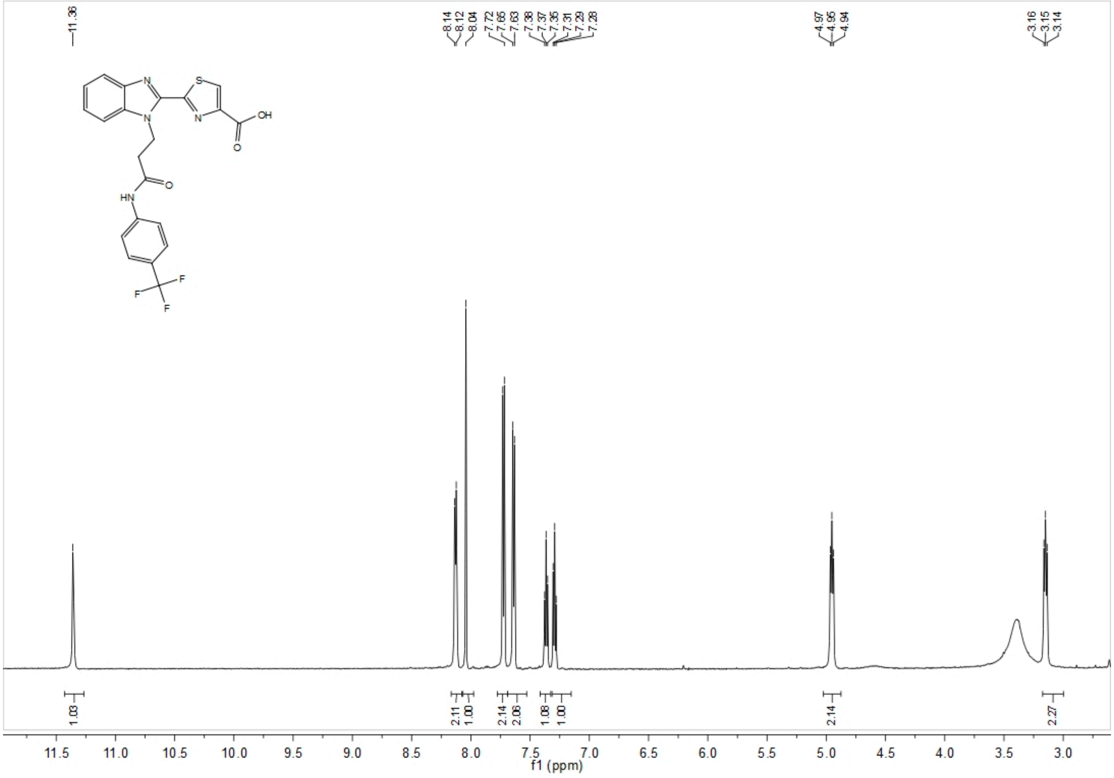

^{1}H-NMR spectra of 13h

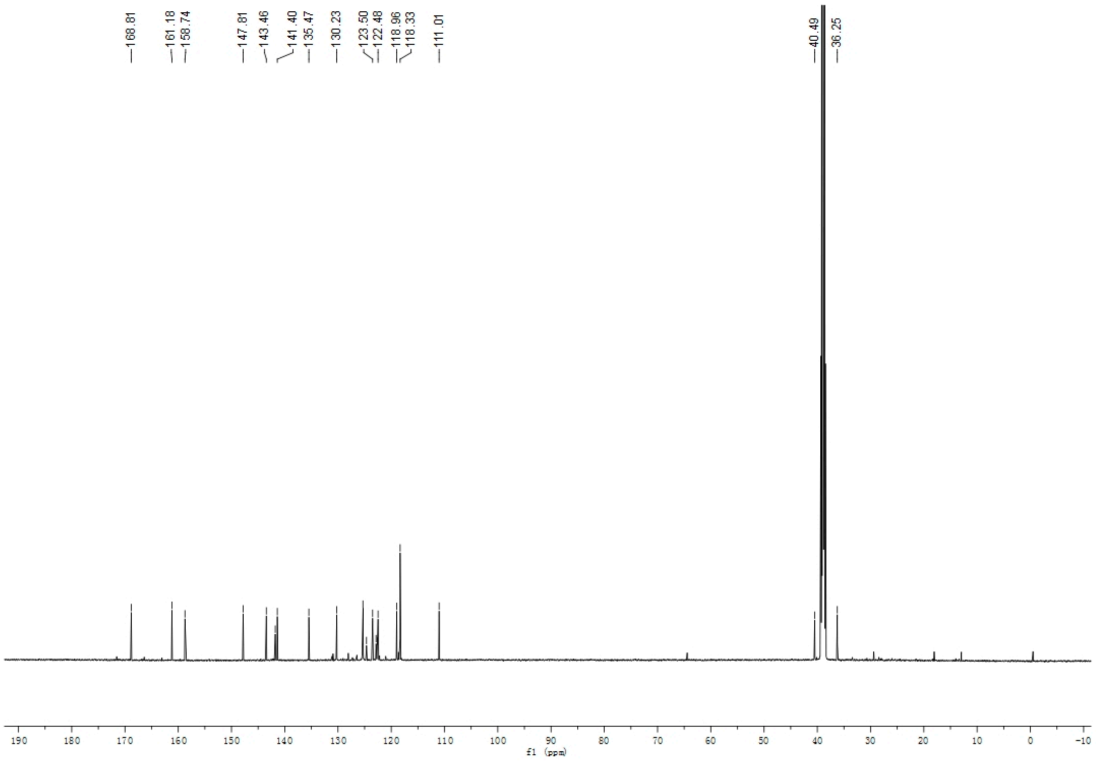

^{13}C-NMR spectra of 13h

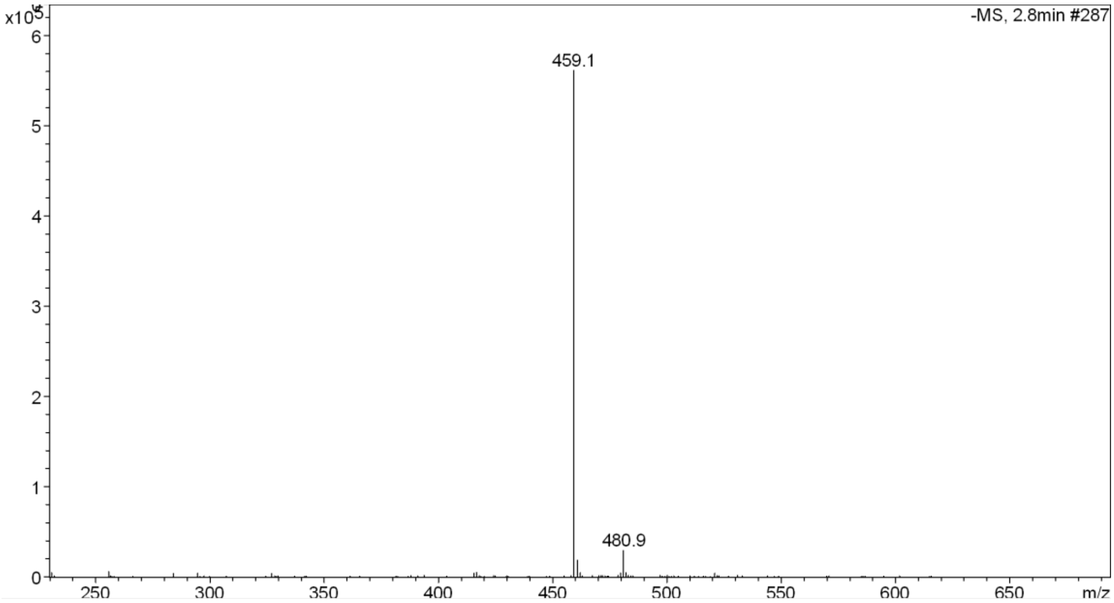

MS spectra of 13h

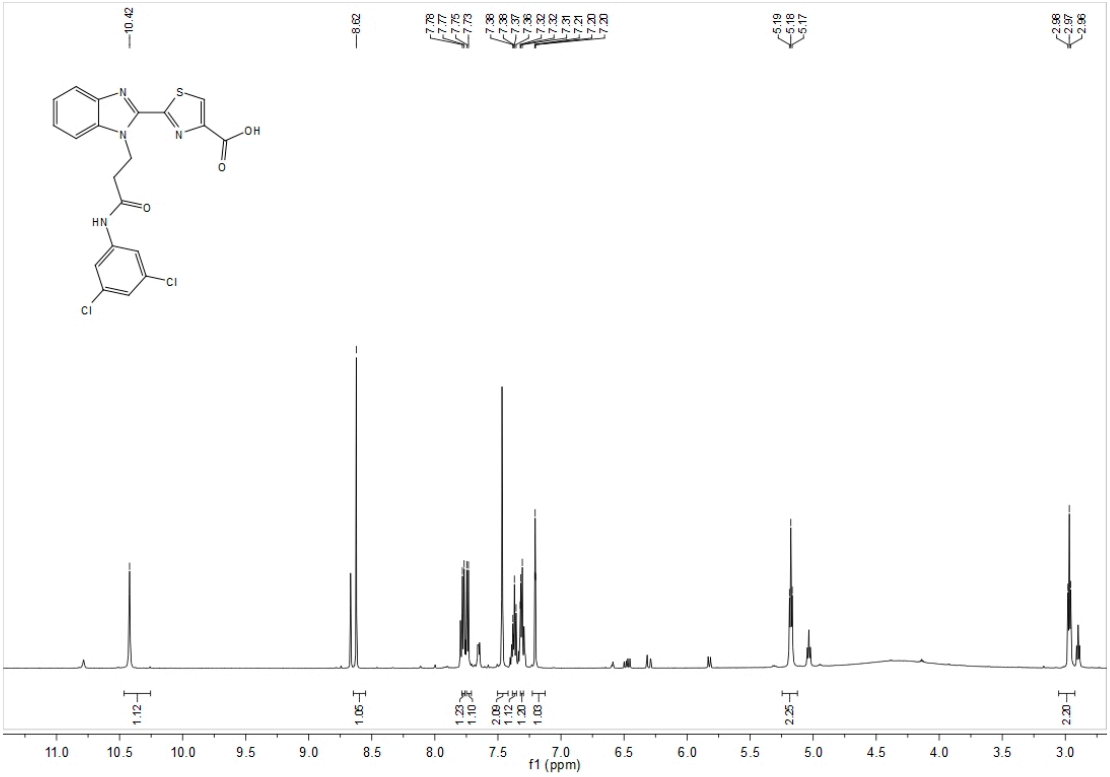

^{1}H-NMR spectra of 13i

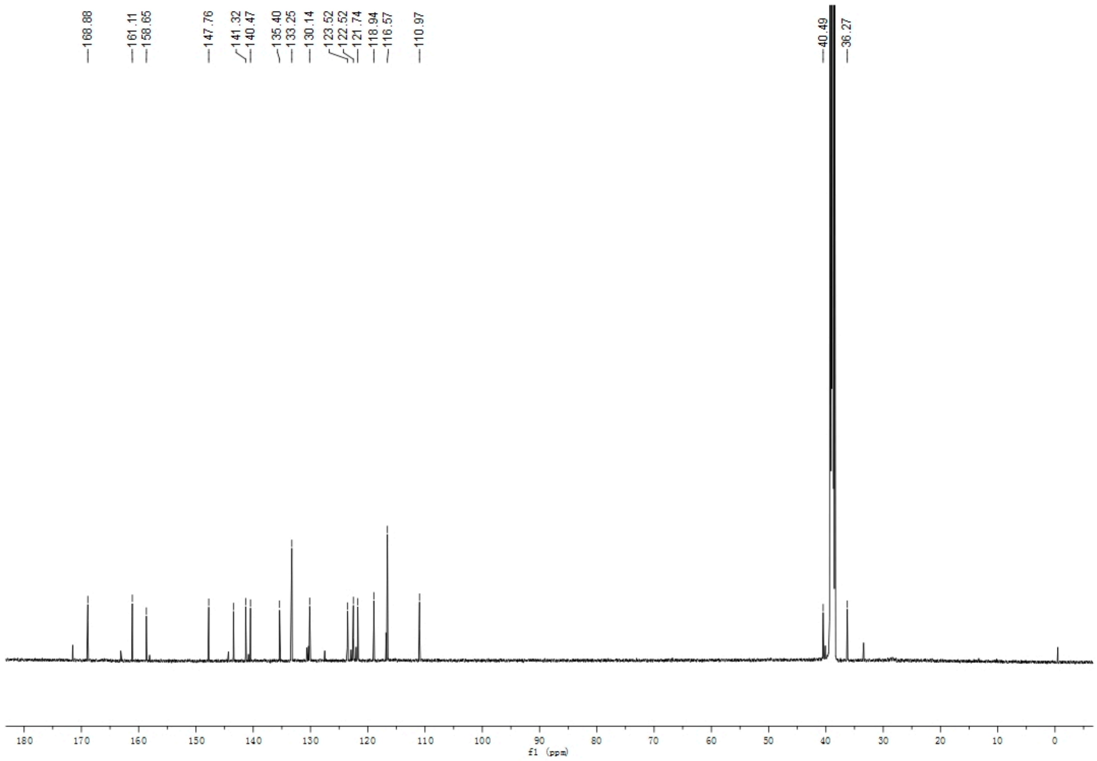

^{13}C-NMR spectra of 13i

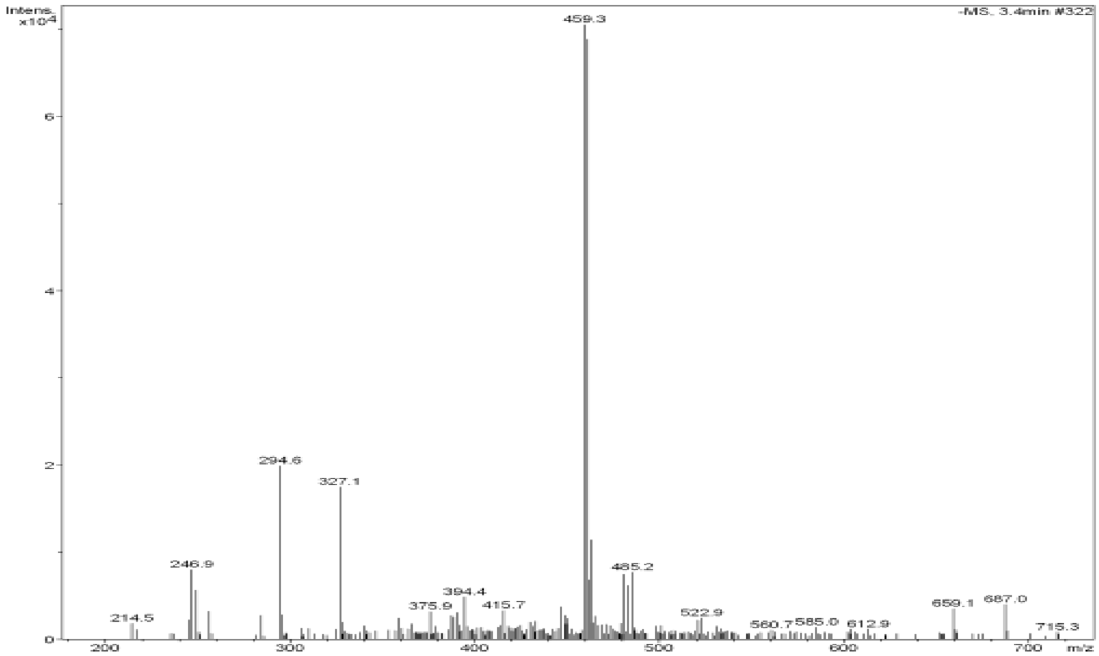

MS spectra of 13i

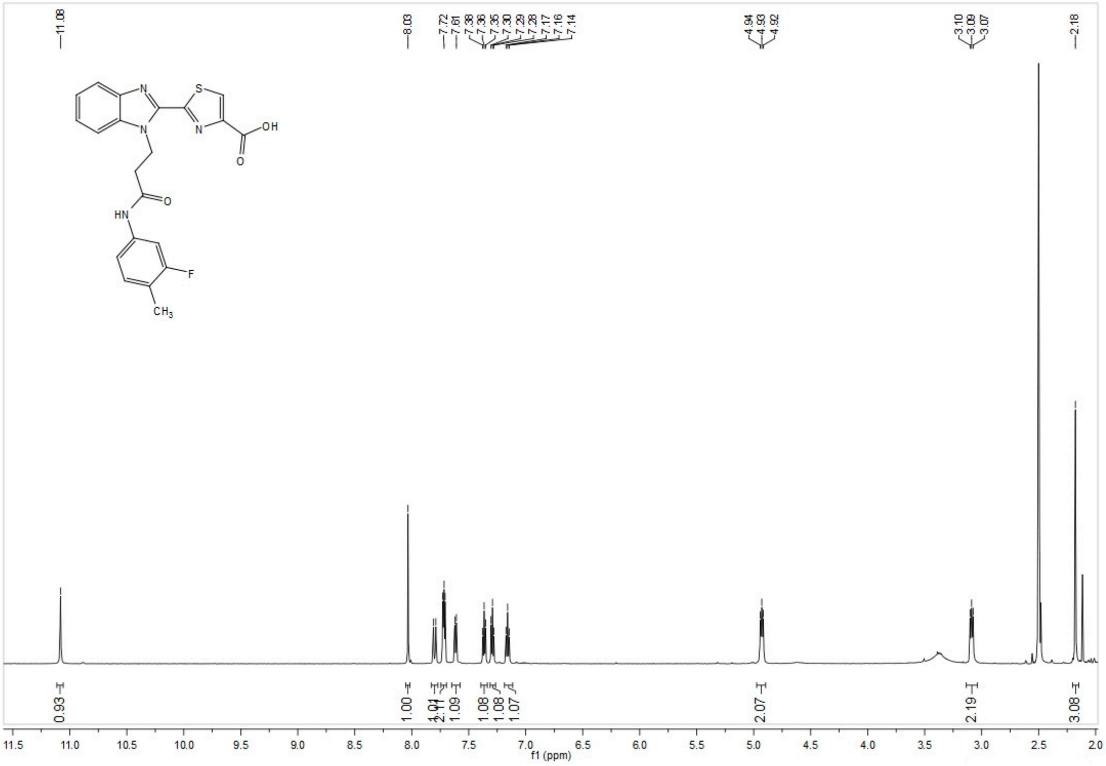

^{1}H-NMR spectra of 13j

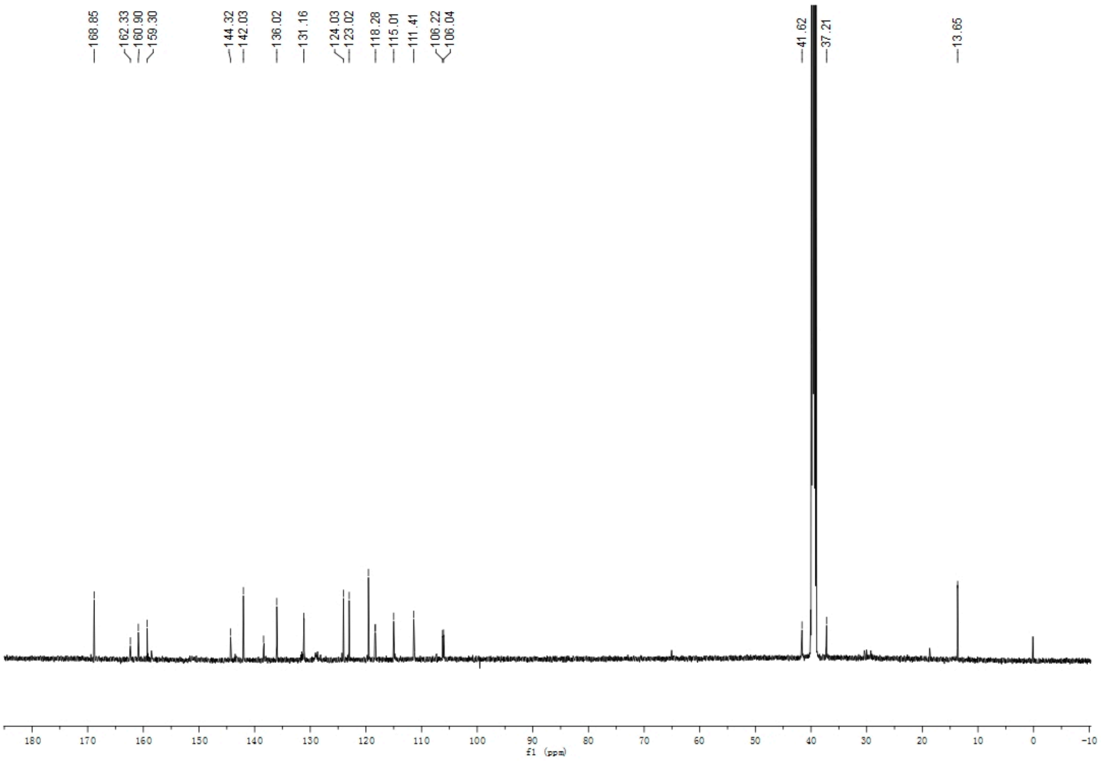

^{13}C-NMR spectra of 13j

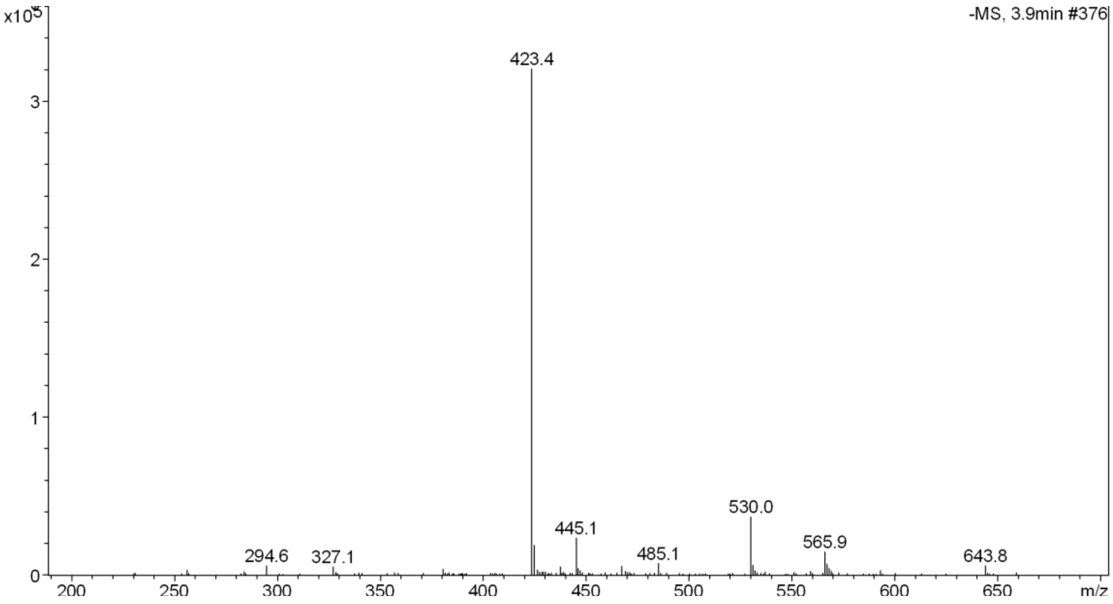

MS spectra of 13j
